# How an ancient, salt-tolerant fruit crop, *Ficus carica* L., copes with salinity: a transcriptome analysis

**DOI:** 10.1038/s41598-019-39114-4

**Published:** 2019-02-22

**Authors:** Alberto Vangelisti, Liceth Solorzano Zambrano, Giovanni Caruso, Desiré Macheda, Rodolfo Bernardi, Gabriele Usai, Flavia Mascagni, Tommaso Giordani, Riccardo Gucci, Andrea Cavallini, Lucia Natali

**Affiliations:** Department of Agriculture, Food, and Environment, University of Pisa, Via del Borghetto 80, I-56124 Pisa, Italy

## Abstract

Although *Ficus carica* L. (fig) is one of the most resistant fruit tree species to salinity, no comprehensive studies are currently available on its molecular responses to salinity. Here we report a transcriptome analysis of *F*. *carica* cv. Dottato exposed to 100 mM sodium chloride for 7 weeks, where RNA-seq analysis was performed on leaf samples at 24 and 48 days after the beginning of salinization; a genome-derived fig transcriptome was used as a reference. At day 24, 224 transcripts were significantly up-regulated and 585 were down-regulated, while at day 48, 409 genes were activated and 285 genes were repressed. Relatively small transcriptome changes were observed after 24 days of salt treatment, showing that fig plants initially tolerate salt stress. However, after an early down-regulation of some cell functions, major transcriptome changes were observed after 48 days of salinity. Seven weeks of 100 mM NaCl dramatically changed the repertoire of expressed genes, leading to activation or reactivation of many cell functions. We also identified salt-regulated genes, some of which had not been previously reported to be involved in plant salinity responses. These genes could be potential targets for the selection of favourable genotypes, through breeding or biotechnology, to improve salt tolerance in fig or other crops.

## Introduction

One of the most important problems for current agriculture is the salinity of irrigated soils. It is estimated that over 6% of the world’s total surface area and about 20% of irrigated lands are affected by salinity^1^ and more than 75 countries are facing salinity problems^2^. In addition, low precipitation, irrigation with brackish water, and inadequate farming practices are causing an expansion of saline areas by about 10% per year. As a result, it has been estimated that over 50% of cultivated land will be salt-affected by the year 2050^3^. In the Mediterranean area soil water availability decreases during the summer because of the rise in temperature and concomitant lack of precipitation. Summer drought increases salinity^4^, since the high evapotranspirative demand and insufficient leaching of ions, favours the accumulation of salts in the soil; a situation further exacerbated by the use of brackish water for irrigation.

The physiological and morphological strategies whereby plants cope with saline stress vary across species. However, in most crops high saline concentrations cause osmotic and ionic stresses, both at the cellular and whole plant level. Salt decreases water and nutrient absorption, reduces CO_2_ availability due to diffusional and photochemical limitations, and modifies carbohydrate partitioning and metabolism^5,6^. Photosynthetic responses to salinity include stomatal closure^7^ and biochemical limitations that decrease mesophyll conductance, strongly limiting CO_2_ diffusion into the chloroplasts^5,8^. Mesophyll limitations further decrease photosynthesis^9,10^ and accelerate senescence in mature leaves. Changes in stomatal conductance and transpiration are common responses in species of medium tolerance to salinity as they limit salt accumulation into the leaves^5,11^.

Excluding Na+ and Cl− at the root level is often the main mechanism whereby plants prevent the accumulation of toxic ions in shoots, leaves, and meristems^12,13^. Cells in the xylem parenchyma, cortex and pericycle can all be involved in the exclusion mechanism, but the Casparian strip in the endodermis effectively blocks the transport of Na+ into the aerial organs, thus controlling the distribution of ions within the plant^12^.

When salts accumulate in plant organs they lower the osmotic potential^13,14^. In addition, changes in carbon (C) and nitrogen (N) partitioning under salinity stress lead to increased concentrations of carbohydrates, amin oacids and other metabolites, that can actively contribute to osmoregulation and protection^5,13,15^. In most tree species, root growth is inhibited as salt reduces water uptake and inhibits the absorption of K^+^, Ca^2+^ and NO_3_^−^ by roots^13^. These primary stresses induce the generation of reactive-oxygen-species (ROS) in the plant^16,17^, may cause hormonal changes^18^ and result in alterations in carbohydrate metabolism^19^. The consequences of these metabolic modifications are a decrease in cell division and the acceleration of cell death^20^. Osmotic stress also reduces the expansion of radical tips, growth and expansion of new leaves, and induces stomatal closure^21^.In halophytes and other tolerant species salts can be extruded out of the leaf tissue via specialized structures such as glands and trichomes^12,22^.

At the molecular level, signalling molecules, such as phospholipids, abscisic acid, jasmonate, brassinosteroids and calcium ions (Ca^2+^), regulate stress signalling pathways for maintaining osmotic potential and regulating plant growth and development, through the induction of changes in gene expression. Genes have been described in these pathways, including those involved in: signalling^23,24^; regulation of transcription, especially through abscisic acid dependent or independent pathways^25^; production of reactive oxygen species and detoxification^26^; membrane transport and production of osmoprotectors, such as proline^23,27^. Finally, genes encoding various ion channels, carriers and pumps are involved in the response to salt-induced cellular changes in ion homeostasis^28–30^.

Fruit trees usually show a greater sensitivity to salinity than annual crops. Among fruit trees of the temperate zone, fig (*Ficus carica* L.) is reported to be moderately resistant to salinity^31^. Recent work showed that fig leaves remained healthy and green upon root exposure to 100 mM sodium chloride (NaCl) for a few weeks, and their gas exchange parameters still remained relatively high^32^. However, concentrations ≥200 mM NaCl resulted in extensive leaf necrosis, leaf abscission, and a dramatic decrease in leaf photosynthesis^32^. Fig trees are also quite resistant to drought and perform well under conditions of moderate summer deficit, mostly in semi-arid climates of the Mediterranean region, Middle East and Asia. Although rainfed cultivation is commonly practiced, growth and productivity of fig trees respond positively to irrigation^33^.

There is currently no information on the molecular response of fig trees to salinity. Considering the relatively modest genetic improvements of current fig cultivars over natural varieties, a genomic approach may be useful to speed up breeding programmes. Recently, drafts of the genome sequence of *F*. *carica* were published^34,35^. By analyzing the gene encoding portion of the genome, genomic DNA-derived transcriptomes were reported^35–37^. Mori *et al*.^35^ provided some validation of their genomic-derived transcriptome using libraries of cDNA derived from a number of organs. *De novo* transcriptome sequencing has also been reported in a few other studies^38–41^.

Despite the growing interest in this crop, related to its potential use in marginal areas and to the nutraceutical value of its fruits^42^, there is little information that quantifies its short or long term resistance to saline stress. Understanding the molecular basis of fig salt tolerance could be useful in addressing genetic improvement for the selection of highly tolerant genotypes. Moreover, identifying genes involved in salt tolerance in a mid-to-high tolerant tree species is a prerequisite to targetting such genes at the biotechnological level (e.g., by gene editing) in order to increase salt tolerance in other tree species.

The objective of the present study was to identify genes of fig plants affected by 3.5 and 7 weeks of irrigation with 100 mM NaCl and to describe gene regulation of the major metabolic processes. We used the cultivar Dottato, which is widely grown in Italy and appreciated for its fruit quality.

## Results

### Shoot growth, leaf chlorophyll, and leaf water relations

**Figure 1:**
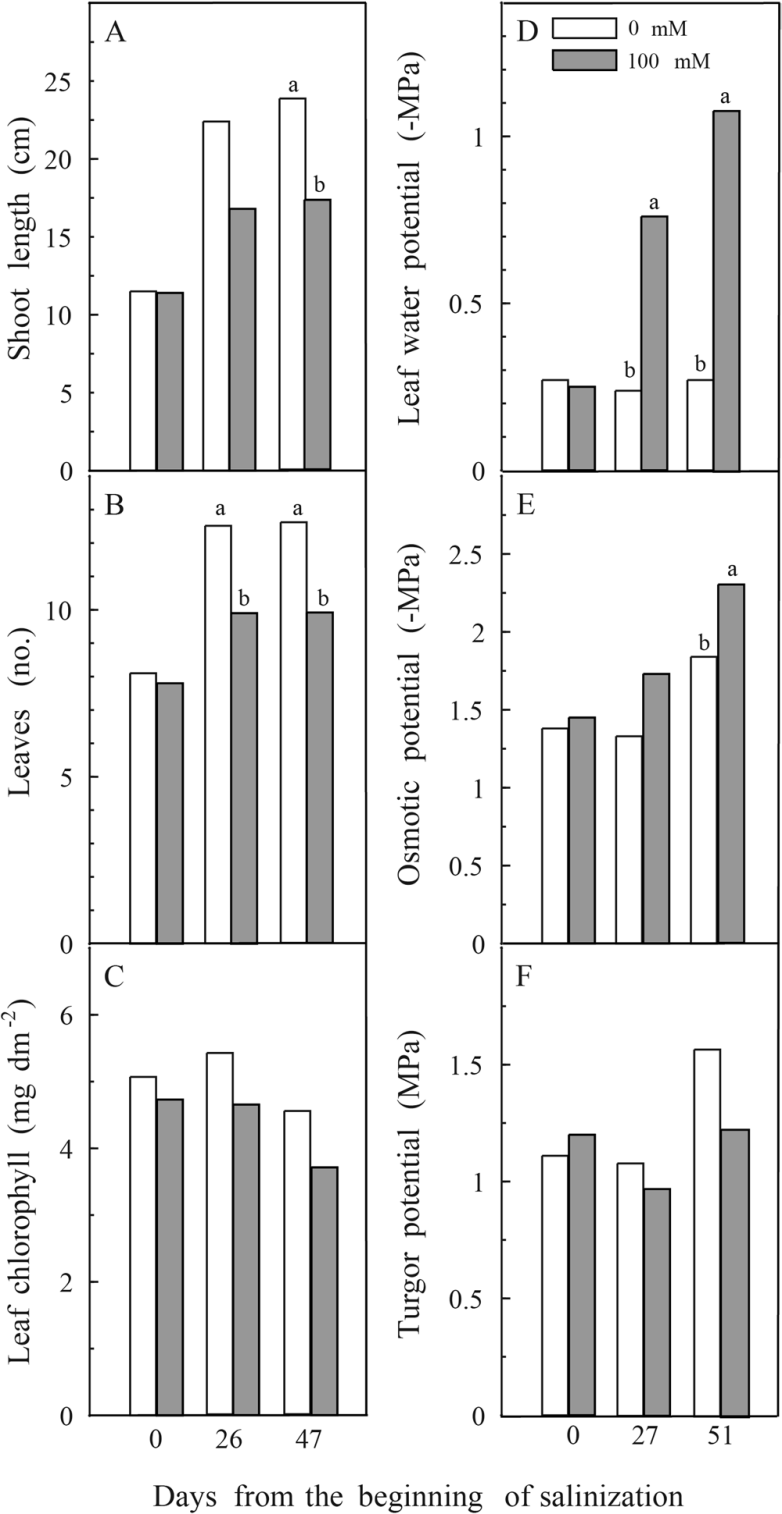
Length of the growing axis (**A**), number of leaves (**B**), leaf chlorophyll content (**C**), leaf water potential (**D**), osmotic potential (**E**), and turgor pressure (**F**) of 0 and 100 mM NaCl treated *Ficus carica* cv. Dottato. Values are means of 12 (**A**–**C**) and 4 (**E**–**G**) replicate plants. Different letters indicate significant differences (*p* < 0.05) between treatments after analysis of variance within each date of measurement.

Salt-treated plants had already shown a slowing down of shoot growth and leaf expansion by the fourth week of treatment. These parameters showed no further increase 47 d after the beginning of salinization, whereas control plants continued to grow (Fig. 1A). There was an average of 13 leaves per control plant by 47 d, whereas plants exposed to 100 mM NaCl had only 10 leaves on average. The leaf chlorophyll content was not significantly different between treatments even at 47 d (Fig. 1B,C). No leaves of either treatment abscised or showed symptoms of damage during the experimental period.

Leaf water potential had decreased significantly by the first sampling date in salt-treated plants and the difference to control plants had become even greater by the end of the experiment (Fig. 1D). The leaf osmotic potential of 100 mM NaCl-treated plants was significantly lower than that of control plants at 47 d (Fig. 1E). As a result of the decrease in osmotic potential salinized plants maintained a turgor pressure of around 1 MPa or higher throughout the experiment (Fig. 1F).

### cDNA sequencing

Twelve cDNA libraries (2 culture conditions, 0 and 100 mMNaCl, for 2-time periods for 3 individuals) were prepared and 248,791,792 sequence reads, each of 125 nt in length, were generated. The total number of tags per library was 1,359–6,163 million (Table S1), a tag density sufficient for quantitative analysis of gene expression^43^. Removal of low-quality reads resulted in 238,539,614 trimmed reads, 100 nt in length, corresponding to a complete dataset of about 53 Gb of sequence data (Table S1). Reads were mapped to the putative transcriptome of *F*. *carica*, cv. Horaishi^35^. The percentage of mapping reads for each sample was 71.59–75.88% (Table S1).

### Global analysis on differentially expressed genes

We evaluated the expression of 36,138 putative coding sequences included in the *F*.*carica* genome assembly^35^. The analysis was limited to genes with RPKM > 1 in at least one of the 3 individuals in at least 1 treatment. In this way we selected 20,759 significantly expressed genes.

**Figure 2:**
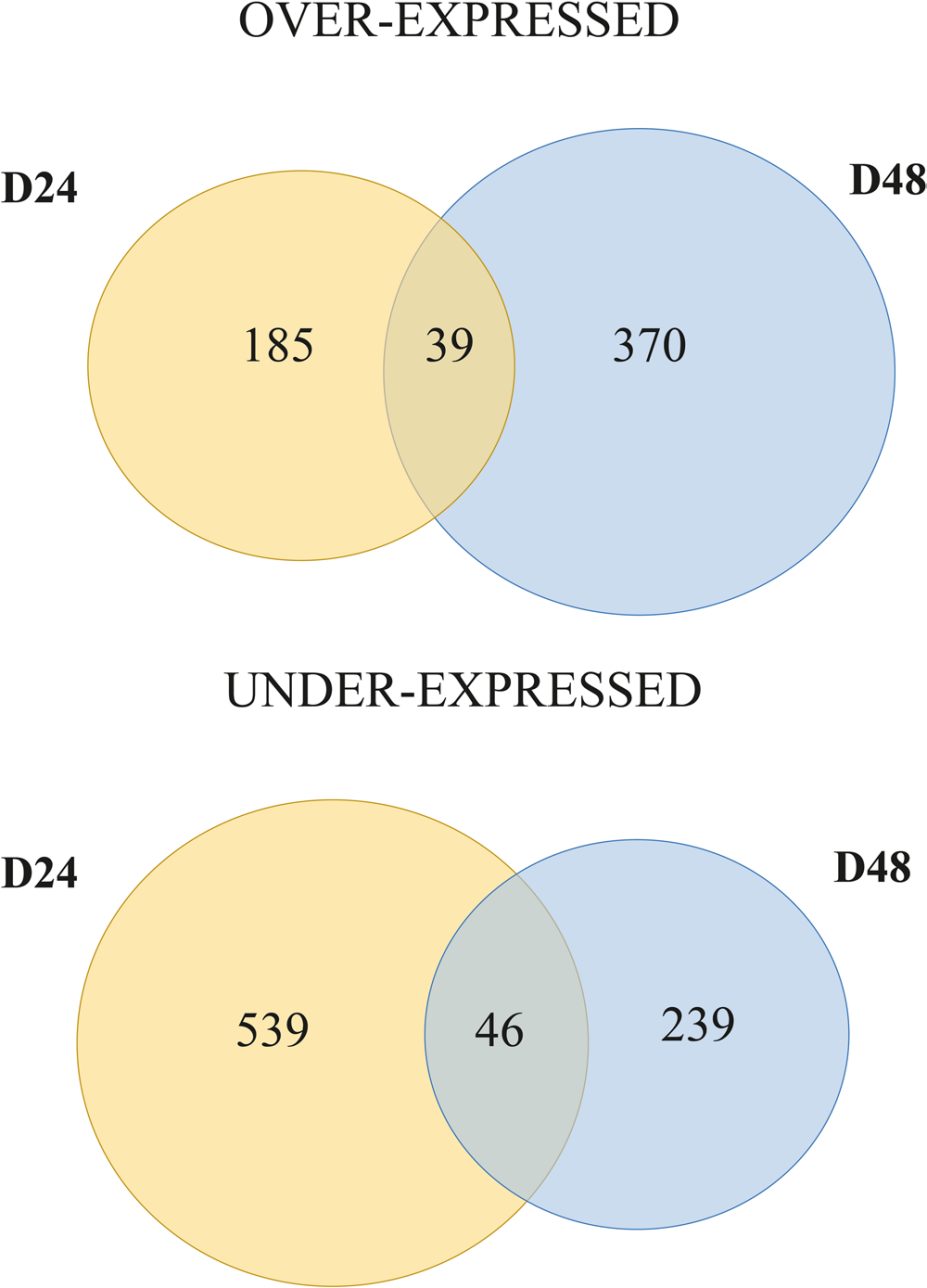
Venn diagrams of over-expressed (above) and under-expressed (below) genes in leaves of *F*. *carica* after salt treatment, compared to leaves of control plants. D24 = pairwise comparison between control and salinity exposed plantsafter 24 days, D48 = pairwise comparison between control and salinity exposed plants after 48 days.

Figure 2 reports the number of genes that were significantly over-expressed or under-expressed in pairwise comparison between leaves of control and salt-exposed plants at 24 d (D24) and 48 d (D48). Overall, we detected 224 over-expressed and 585 under-expressed genes at 24 d, and 409 over-expressed and 285 under-expressed genes at 48 d. Quite a large number of over-expressed genes were specifically induced by 48 d (D48) of 100 mM NaCl treatment compared to untreated plants, while the majority of DEGs at 24 d (D24) were down regulated. The number of genes differentially expressed at both time points (24 d and 48 d) was quite low (85; Fig. 2).

### Differential expressed genes at 24d and 48 d

At 24 d, 224 transcripts were significantly up-regulated and 585 were down-regulated in saline treated plants compared to the control (Fig. 2). Amongst the 100 most up- and down-regulated genes we found some involved in ROS signalling (*Tubby-like protein 8*, *Nodulation signalling pathway*, *RBK2*), transcription regulation (*terminal-flower1-like*), transduction (*LURP1*, *RPM1*) and translation regulation (*26S proteasome*; Table S2).

Significantly enriched GO terms occurred only in the down-regulated gene set (Fig. S2). At 24 d, the most abundant enriched down-regulated GO terms were “metabolic process” (GO:0008152) and “cellular process” (GO:0009987), possibly reflecting initial cell metabolic switch-off caused by salt stress. No GO terms were enriched concerning over-regulated genes.

At 48 d, 409 genes were significantly up-regulated and 285 genes were significantly down-regulated by saline treatment of plants (Fig. 2). Among these up-regulated genes, we identified those involved in amino acids metabolism (*P5CS*), regulation of translation (*HSF*), transport (*ERD-6*, *SLAH3*) and hydrolase activity (*Expansin-like* protein; Table S2).

By 48 d, significantly enriched GO terms occurred only in the up-regulated gene set (Fig. S3), whereas no GO terms were enriched concerning down-regulated genes. The most frequent enriched up-regulated GO terms were “metabolic process” (GO:0008152) and “catalytic activity” (GO:0003824), suggesting a late metabolic switch-on related to prolonged salt treatment. Among enriched GO terms, at 48 d, we found “Proline biosynthetic process” (GO:0006561), “Glutamate-5-semialdehyde dehydrogenase activity” (GO:0004350), “Transmembrane transport” (GO:0055085), “Phosphotransferase activity” (GO:0016774), “Carboxyl group as acceptor” (GO:0016774), and “Saccharopine dehydrogenase activity” (GO:0004753).

In order to validate the *in silico* analysis, qRT-PCR analysis was performed on 5 DEGs. Four of the DEGs were up- or down-regulated at 24 d of salt treatment and 2 were differentially expressed after 48 d of salt treatment (one of these was up-regulated at both 24 d and 48 d, Table S3). Comparing salt-stress versus control samples by ANOVA showed significant values (*p* < 0.05) for the 4 DEGs tested at 24 d and one of 2 DEGs at 48 d. The second DEG at 48 d was over-expressed with 0.05 < *p* < 0.1 (Table S3). Overall, qRT-PCR of these 5 DEGs was comparable to the analysis for these genes with RNA-seq.

### GO comparison and gene modulation between 24 d and 48 d

Based on GO-slim annotations, up- and down-regulated genes were classified into 3 main ontological categories (cellular component, biological process, and molecular function), keeping separated significantly over-expressed and down-regulated genes, and comparing 24 d to 48 d. The GO terms of over-expressed genes are reported in Fig. S4. Overall, a similar number of GO terms were identified at 24 d and 48 d, with 93 and 98 GO terms, respectively. Considering over-expressed genes within the biological process category, the most frequent category in 24 d and 48 d plants was “Metabolic Process”; within cellular components, the most frequent term was “Membrane”; and within molecular function, “Nucleotide Binding” (Fig. S4).

Up-regulated GO terms increased their number at 48 d compared to 24 d (Fig. S4), probably reflecting the higher number of over-expressed genes. However, the frequency of some GO terms such as “Membrane”, “Cellular Component Organization”, and especially “RNA-binding” were significantly higher at 24 d than at 48 d.

With regard to the most frequent GO terms for under-expressed genes at 24 d and 48 d (Fig. S5), we retrieved many involved in “Biosynthetic Process” (for the biological process category), “Membrane” (for the cellular component category), and “Protein Binding” (for the molecular function category; Fig. S5). Many under-expressed GO terms were found to be less represented at 48 d compared to 24 d (Fig. S5). Nevertheless, terms such as “Photosynthesis”and “Thylakoid” were significantly down-regulated at 48 d.

The 85 DEGs observed at both 24 d and 48 d are reported in Table S4. For most of these, the up-regulation level was similar between 24 d and 48 d. Only 2 transcripts, encoding an uncharacterized protein and a *subtilisin-like protease*, showed a log fold change (FC) increase >1, and only 1 transcript, encoding a *B3 domain-containing transcription factor VRN1*, had a log FC decrease <−1 (Table S4).

Concerning genes down-regulated at both 24 d and 48 d, their log FC values were in most cases similar. Some genes showed a logFC > 1 between 24 d and 48 d, such as those encoding *RING finger* and *CHY zinc finger domain-containing* proteins, *pectinesterase*, *transcription factor bHLH93*, *DnaJ homolog subfamily C*, *(RS)-norcoclaurine 6-O-methyltransferase*, *AP2/ERF and B3 domain-containing transcription factor RAV1*, *plasma membrane intrinsic protein* 1, *GAST-like gene and* chaperone protein *dnaJ 11* (Table S4). Only 1 transcript encoding for *Polcalcin Jun o 2* showed a decreasing logFC < −1 between 24 d and 48 d (Table S4).

### Functional categorization of salt stress DEGs

Functional categorization was analysed using MapMan software^44^, comparing DEGs at 24 d with those at 48 d, in order to identify the processes involved in salt-induced physiological changes.

**Figure 3:**
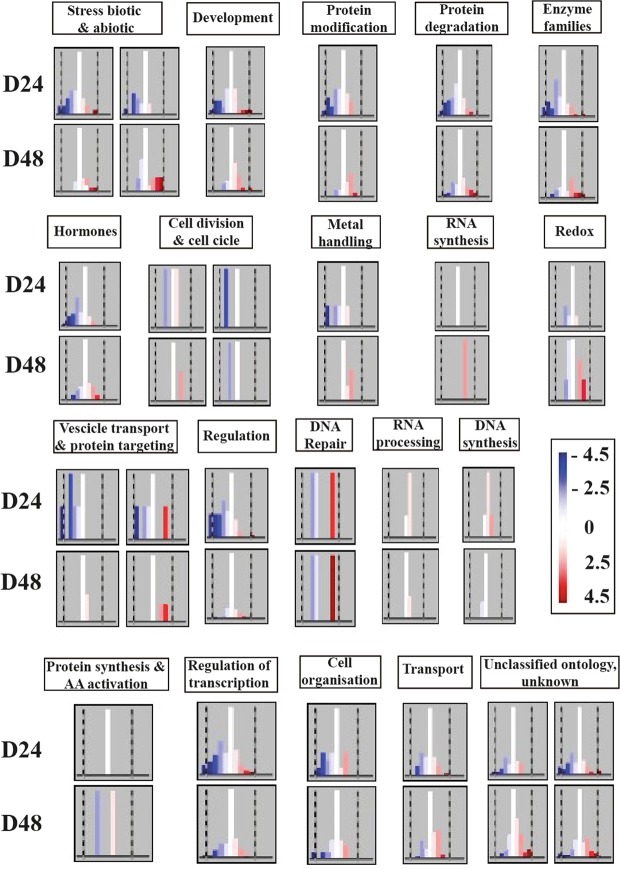
MAPMAN distributions of down- to up-regulated genes involved in different cell functions at 24 d (D24; above) and 48 d (D48; below) of salt treatment. White columns indicate genes which were not differentially expressed at that stage but were differentially expressed at the other stage. The scale ranges from dark blue (log FC < −4.5) to dark red (log FC > 4.5).

Many cell functions showed down-regulation at 24 d, with many repressed genes. This down regulation reverted by 48 d, when the number of down-regulated genes had strongly decreased, and up-regulated transcripts had increased. This was the case of genes involved in biotic/abiotic stress, development, protein modification/degradation, enzyme families, hormone biosyntheses, cell division, metal handling, transport/targeting, and regulation (Fig. 3).

Other cell functions were unaffected overall at 24 d and only activated by 48 d, such as reduction-oxidation reactions (Redox) and, especially RNA synthesis. These functions seemed to be specifically related to long-term adaptation to salt stress. Among genes involved in these functions and strongly up-regulated at 48 d, we found genes encoding a putative *plant-specific RNA polymerase IV*, a *copper/zinc superoxide dismutase copper chaperone*, and a *2-oxoglutarate-dependent dioxygenase* (Fig. 3). RNA processing and DNA synthesis categories were slightly activated at 24 d but were almost unaffected at 48 d. This suggested these cell functions were probably induced in the first stages of salt stress.

Many DEGs associated with metabolism functions showed changes in their expression between 24 d and 48 d (Fig. S6). Especially interesting was the overall increase in expression of genes involved in the pathways related to secondary metabolism, such as those for phenylpropanoids and phenolics (e.g., encoding *HXXXD-type acyl-transferase family* protein, *cytochrome P450 98A2* and *cytochrome P450 71D10*), flavonoids (e.g., encoding *UDP-glycosyltransferase superfamily protein 2-oxoglutarate* and *Fe(II)-dependent oxygenase superfamily protein*), and waxes (e.g., encoding *ECERIFERUM 1*; Fig. S6).

**Figure 4:**
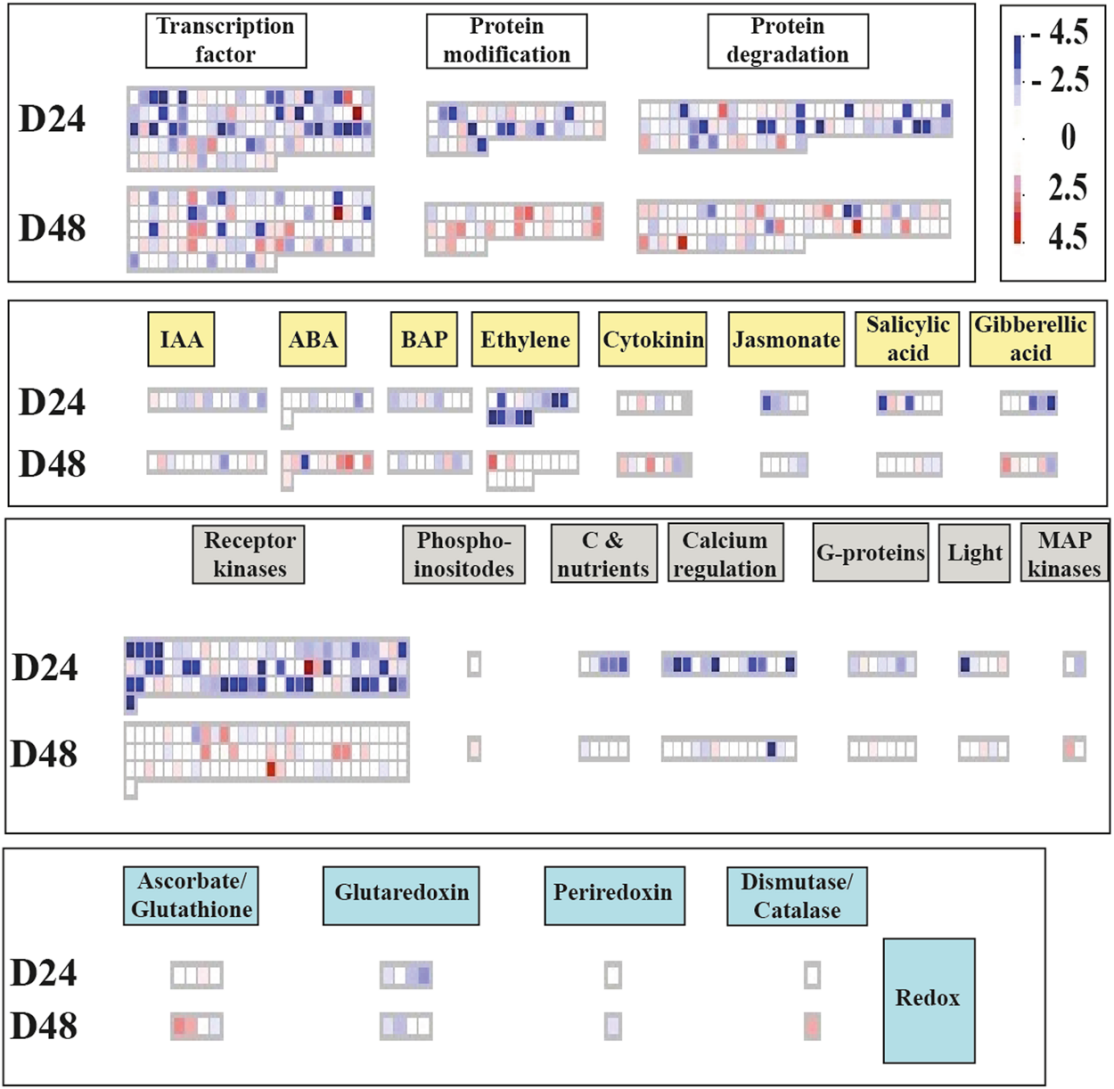
MAPMAN sketch of regulation pathways comparing up- and down-regulated genes (small squares) under salt treatments at 24 d (D24; above) and 48 d (D48; below). The differential expression scale ranges from dark blue (log FC < −4.5) to dark red (log FC > 4.5). Grey circles indicate that the genes involved in that function were not found in salt-regulated DEGs.

An overall picture of regulation pathways and related DEGs is shown in Fig. 4. A general down-regulation of genes involved in functions such as transcription factors, protein modification, protein degradation, receptor kinases and calcium regulation, occurred at 24 d. All these classes showed a recovery during the second part of experiment (24 through 48 d after salinization), and in many cases showed an up-regulation of transcripts.

Among genes of hormone production and reception, those related to ethylene, jasmonate and gibberellic acid were also down-regulated at 24 d and recovered, or were up-regulated, by 48 d (Fig. 4). In the Redox system, up-regulation of the ascorbate/glutathione cycle and over-expression of a gene belonging to the dismutase/catalase family were observed at 48 d (Fig. 4).

**Figure 5:**
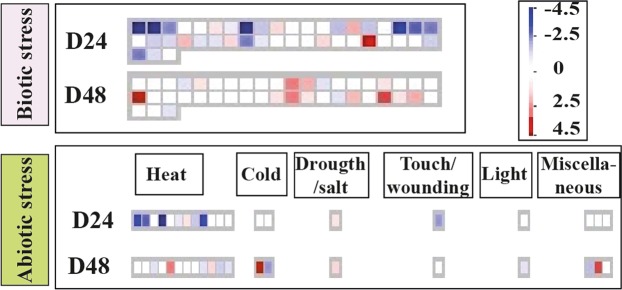
MAPMAN sketch of biotic and abiotic stress pathways comparing up- and down-regulated genes (small squares) under salt treatments at 24 d (D24; above) and 48 d (D48; below). The differential expression scale ranges from dark blue (log FC < −4.5) to dark red (log FC > 4.5).

The MapMan pathways related to biotic stress (Fig. 5) showed many genes that were down-regulated at 24 d and recovered their expression at 48 d. Many genes unaffected at 24 d were activated by the late phases of the experiment (e.g., encoding *late embryogenesis abundant protein*, *hydroxyproline-rich glycoproteins*, *cysteine-rich secretory proteins* and *disease resistance-responsive proteins*).

With regard to the abiotic/biotic stress pathways (Fig. 5), only 2 genes were moderately activated at 24 d, a double *Clp-N motif-containing P-loop nucleoside triphosphate hydrolase* gene (involved in heat stress response) and an *early-responsive to dehydration stress* gene (involved in drought/salt response). This low number of activated genes was consistent with the concept that fig trees tolerate salinity well. Indeed, at 24 d the 100 mM NaCl treated fig plants did not show signs of distress.

At 48 d, only 4 abiotic stress genes were lightly or moderately activated, encoding a chaperone DnaJ-domain superfamily protein and heat stress transcription factor (involved in heat response), a *CAP160* protein (described as involved in cold response), an *early-responsive to dehydration stress* protein (of the drought/salt pathway) and a *cysteine-rich secretory protein*, *antigen 5* (belonging to a miscellaneous pathway).

## Discussion

*F*. *carica* L. is moderately resistant to salinity with a range for safe growth between 0 and 100 mM NaCl^31,32^. After 7 weeks of 100 mM NaCl treatment leaves did not show any apparent symptom of toxicity and, although the expansion of new leaves and shoot growth were inhibited, plants appeared in good health and leaves retained a chlorophyll content similar to control leaves (Fig. 1). Leaf chlorophyll content was affected only at salinity levels higher than 100 mM NaCl (data not shown), as already reported^45^. It has been suggested that chlorophyll degradation is caused by oxidative stress damage at high saline concentrations^46^ and the inadequacy of antioxidant molecules and light energy dissipation mechanisms^46–48^.

Our current experiments confirmed that fig plants could withstand exposure to 100 mM NaCl for several weeks^32^ and that their resistance was comparable to that exhibited by olive plants^14^. In addition, we showed that changes in osmotic potential paralleled those in leaf water potential, which allowed turgor pressure to remain high and similar to values in control plants (Fig. 1). Osmotic adjustment has been shown to be an effective mechanism of salinity tolerance for many crops^14,21^.

Transcript profiling of 100 mM NaCl-treated plants produced novel results. The global gene expression analysis indicated that, while 24 d treatment with saline resulted in many repressed genes, suggesting down-regulation of cell functions, 48 d treatment showed a dramatic change in the repertoire of genes expressed. Nevertheless, considered that expressed genes could undergo post-transcriptional silencing, production of mRNA does not necessarily imply changes in the level or activity of the cognate protein.

At the 24 d sampling date salt-treated plants showed physiological changes and global cell transcriptome rearrangement. Down-regulated genes were mostly implied in metabolic and cellular processes such as cellular division or hormone biosynthesis. The main effect of early salt treatment was likely the result of a general reduction of transcription, affecting cellular machinery. No GO term was significantly enriched in the 24 d over-expressed gene set, and RNA processing was one of the few cell functions affected by early salt treatment. Therefore, we propose that plants responded to initial salt exposure with morphological and biochemical changes that did not require dramatic changes in gene expression. Genes over-expressed at 24 d include those encoding *tubby-like protein 8* and *nodulation signalling pathway 1*, which are implicated in reactive oxygen species (ROS) signalling^49,50^. A receptor-like protein also over-expressed was the cytosolic serine/threonine-protein kinase *RBK2*, previously described as regulated in cadmium phytoremediation^51^. It can be hypothesized that, at 24 d, activation of receptors and signalling-related proteins would in turn modulate cascades, such as indicated by the observed over-expression of ABA dependent (e.g., myb-related protein *MYBAS1* and *bHLH10-like*) and ABA independent (e.g., *NF-Y*) transcription factors, which are known to be involved in salt tolerance^52^. *TERMINAL-FLOWER1-like* gene was another over-expressed transcription factor, reported to be highly expressed in rice salt tolerant genotypes^53^. In addition, at 24 d salinity induced the expression of genes encoding transport proteins such as nitrate transporter, sulfate transporter and putative inorganic phosphate transporter 1–7, similar to a report on abiotic stresses in *A*. *thaliana*^54^. We also observed the over-regulation of genes reported previously as active in plant biotic (e.g., encoding *LURP1* and *disease resistance protein*^55^) and abiotic stresses (e.g., encoding *S-adenosylmethionine-dependent methyltransferase* and *agamous-like MADS-box protein AGL8*^56,57^. Increased transcript levels for a few genes implicated in protein degradation and cell death during salt-stress were identified at 24 d, such as 2 sequences encoding for disease resistance protein *RPM1*, that facilitates increase of cytosolic calcium essential for the oxidative burst and hypersensitive cell death^58^, 26S proteasome regulatory particles, some F-box proteins and *F-box/LRR-repeat* proteins^59–61^. At 24 d of salt treatment a pentatricopeptide repeat transcript involved in splicing during abiotic stress in rice^62^ was also relatively overexpressed.

At 48 d, most cell functions that were apparently down-regulated at 24 d were reactivated, suggesting that plants actively responded to salinity by adaptation. In terms of gene expression, we observed many typical responses to salt stress. For example, several activated genes were involved in the glutamate and proline pathways. Glutamate is involved in the switch of osmolyte strategy from glutamate to proline as the dominant compatible solute during the transition of *Halobacillus halophillus* from moderate to high salinity^63^. Proline is synthesized mainly from glutamate, which is reduced to glutamate-semialdehyde (GSA) by *pyrroline-5-carboxylate synthetase* (*P5CS*)^64^. Proline can act as a signalling molecule to modulate mitochondrial functions, influence cell proliferation or cell death and trigger specific gene expression, which can be essential for plant recovery from stress^65,66^. Considering the proline pathway, we also detected the over-expression of *delta-1-pyrroline-5-carboxylate synthase* (*P5CS*) encoding gene, involved in proline biosynthesis and abiotic resistance^67^. Furthermore, we retrieved DEGs involved in ABA dependent (e.g., *MYB* and *WRKY*) and independent (e.g., *HSF*, *A-6b*) regulation pathways. Expression of genes in proline biosynthesis during abiotic stresses is activated by both these pathways in *A*. *thaliana*^52^.

Salt treatment at 48 d also over-regulated genes involved in the production of other osmoprotectants^68^, such as those encoding raffinose synthase family proteins, which have been linked to high salinity stress in *A*. *thaliana*^54^.

An over-represented GO category at D48 was transmembrane transport systems. This group of genes plays a significant role in plants adjusting to lack of water^69^. Various channels, carriers and pumps work towards achieving ion homeostasis during salt stress^13,25^ and ion transporters are similarly considered to play a vital role in salt tolerance^70^. At D48 we retrieved over-expressed transcripts encoding for transporters (e.g., bidirectional sugar transporter *SWEET16*, sugar transporter *ERD6-like 6*, transmembrane amino acid transporter) and channels (S-type anion channel *SLAH3*), all probably involved in homeostatic regulation.

Saline treatment at 48 d also induced the over-expression of many transcripts encoding signalling components, such as a *leucine-rich repeat receptor-like protein kinase*, *highly ABA-induced PP2C*, *EID1-like* and *F-box protein 3*. Interestingly, most of these genes have redundant functions in ABA signalling and proline accumulation^71–74^. Furthermore, among signalling components, a putative inactive *purple acid phosphatase 27* transcript was over-expressed; this gene was described as under-expressed in response to water deficit in wheat^70^. Salt treatment also determines the production of ROS^13^. In our experiments we observed the over-expression of genes encoding *cellulose synthase-like protein G2* and *pentatricopeptide repeat-containing* proteins, involved in ROS level regulation^75,76^.

With regard to cell wall metabolism, an *expansin-like* encoding gene was up-regulated at 48 d, suggesting cell wall component modifications during salt treatment. Expression of this gene family has been shown to confer resistance to salt-stress^77^.

Many up-regulated genes at 48 d were involved in the response of plants to biotic (e.g., encoding putative disease resistance, *yellow leaf-specific gene 9*, *pathogenesis-related*) and abiotic stress (e.g., encoding *DnaJ homolog subfamily B member*, *CAP 160*, *RING-H2 finger*)^55,78,79^.

Some genes were differentially expressed at both 24 d and 48 d; 39 genes over-expressed and 46 genes under-expressed. The activation or inactivation of such genes could be considered a core molecular response response of a medium salt tolerant tree, because these genes changed their expression by 24 d and seemed to maintain this response until 48 d. Among them, we identified an over-expressed gene encoding a senescence-associated protein. In other species senescence was induced by salt stress in order to reduce photosynthetic rate^80^. Since no sign of leaf abscission was observed in 100 mM salt-treated fig plants over the 48 d period of experiment, it can be hypothesized that over-expression of this gene represents a sort of preliminary activation of salt stress induced senescence.

Salinity also induced up-regulation at both 24 d and 48 d of a *DNA ligase* gene, which had previously been shown to be involved in preservation of seed potency and fast seed germination^81^, and of a *ribonuclease J* (*RNJ*) gene, which plays a vital role in chloroplast development and in embryo cell fate determination^82^.

Considering broad functional categorization of genes regulated by salt stress in the fig leaves allowed us to confirm that several processes were apparently depressed by 3.5 weeks (24 d) after the start of salt treatment, then reactivated by 3.5 weeks later (48 d), indicating that many biochemical changes induced by salinity were related to gene regulation changes after prolonged salt treatment (>24 d; Fig. 3).

It is worth noting that regulation of transcription (Fig. 4) and stress response (Fig. 5), processes, usually associated with the first stages of stress, showed an overall activation only after 3.5 weeks (24 d), but before 7 weeks (48 d) of salt treatment. Also, general metabolic processes were affected by long term salt treatment. It is known that the activation of secondary metabolism is implicated in environmental adaptation and stress tolerance^83^. Among metabolism pathways, the glycolysis pathway is known to be activated by NaCl stress in cucumber seedlings^84^. In our experiments, some genes involved in glycolysis (e.g., encoding *enolase*, *phosphoenolpyruvate carboxylase kinase 1* and *glucose phosphomutase*) were down-regulated; others (e.g., encoding *glycosyl hydrolases family 3*, *extensin 4* and *glycoside hydrolase family 32*) were up-regulated, suggesting a modulation of these molecular families, though it was difficult to determine the net effect. Genes involved in cell wall metabolism, known to be involved in salt stress detection and tolerance^85^, were also identified in our analyses (e.g., encoding *expansin-like B1*, *cellulose synthase like G2* and *cellulose synthase like G3*). In many cases, these genes were down-regulated in the first half of the experiment (24 d), and then they showed expression levels similar to control plants by 48 d. Another interesting class included lipid metabolism-related genes, which are known to be regulated during the onset and development of salt stress in plants^86^. In our experiments, some genes involved in lipids metabolism were moderately down-regulated at 24 d and mildly up-regulated at 48 d. Amino-acid metabolism is also reported to increase during salt stress response^87^. We also retrieved genes up-regulated at 48 d involved in amino acids metabolism including those of the *aspartate-glutamate racemase* family and *lysine-ketoglutarate reductase/saccharopine dehydrogenase*.

Finally, we were able to detect some salt-induced genes not previously associated with a mid-high salt stress response in plants. At 24 d, these included: *NUCLEAR FUSION DEFECTIVE 4-like isoform X1*, that encodes a mitochondrial ribosomal protein^88^, expressed during root nodule development in *Medicago truncatula*^89^; a gene encoding the *SPOUT methyltransferase*, which is involved in methylation of lysine domains and ribosomes^90^; and another encoding a mono-ubiquitin, showing similarities with a *Fanconi anemia group 1 protein*^91^. A few genes not previously associated with salt stress were also found over-expressed in salt treated *F*. *carica* at 48 d. Amongst these, we identified genes encoding an *Atrophin-1*, implicated in developmental processes^92^, and an *E3 ubiquitin-protein ligase listerin* (*Ltn*), involved in the elimination of mRNAs lacking a stop-codon^93^.

In conclusion, this study identified a repertoire of salt-regulated genes and determined new genes which had not previously been associated with a response to salinity. To the best of our knowledge, the present work is the first comprehensive transcriptome analysis of leaves of fig plants exposed to salt stress, and represents the first attempt at deciphering the molecular basis of the medium salt tolerance of this species. Our results provide the background information for further studies aimed at evaluating the effect of brackish water irrigation on field-grown fig trees and their fruit. In this respect, *F*. *carica* qualifies as a potential crop for drained, unfertile soils or coastal areas. In addition, the genes identified in this study may be useful in future studies as targets for gene editing aimed at increasing salt tolerance in fig or in other tree species.

## Materials and Methods

### Plant material and physiological measurements

Two-year old, micropropagated plants of *Ficus carica* (cv. Dottato) were used for the experiments. Forty-eight plants were each grown in 5 L plastic pots filled with a mixture of 6.4% clay, 8.6% silt, and 85% sand, then topped with peat to avoid water percolation along the sides of the pot. Prior to the beginning of the experiments the height and diameter of the lignified stem, the number of fully expanded leaves, and the number of total leaves per plant were measured. Plants were assigned to treatments according to size classes to achieve size uniformity across treatments. All plants were 0.6–0.8 m in height and had at least 6 fully expanded leaves on the day the experiment was started.

Plants were grown outdoors in June and July 2016 and subjected to 4 salinity concentrations (0, 50, 100, and 200 mM NaCl), as previously described^32^ (Fig. S1). Transcript profiling was carried out only on the control (0 mM) and 100 mM NaCl treated plants. The 100 mM NaCl concentration was chosen because in previous experiments we showed that fig leaves remained green and apparently healthy for >7 weeks despite the accumulation of ions in the mesophyll^32^. The saline solutions were obtained by adding different amounts of pure (>99.8%) NaCl (Sigma-Aldrich Co., Denmark) to distilled water. The plants were irrigated 3 times a week; during the first 4 weeks each plant received 400 ml of water at each irrigation, then the volume was increased to 700 ml. Plants were protected from natural precipitation.

Shoot length, number of leaves, leaf chlorophyll content, leaf water potential (LWP), and leaf osmotic potential were measured in the same week that leaf tissue for transcript profiling was sampled.The LWP was determined on fully-expanded leaves (4 plants per treatment), excised with a sharp blade, using a Scholander-type pressure chamber^14^. The latex exuding from the petiole cut-end after leaf excision was immediately blotted dry, and then the leaf was pressurized at a rate of 0.02 MPa s^−1^. Plants were enclosed in black plastic bags on the evening of the day before measurement and maintained in the dark until pressurized (all measurements were taken between 6:30 a.m and 7:30 a.m). After LWP determination, leaf blades were frozen and kept at -20 °C for determination of osmotic potential using a Wescor 5500 vapour pressure osmometer^14^. Turgor pressure was calculated as the difference between LWP and osmotic potential.

The leaf chlorophyll content was measured nondestructively using a SPAD-502 unit (Konica Minolta, Osaka, Japan), that measured transmission at 600–700 nm and 900–1000 nm wavelengths. The readings had previously been correlated with chlorophyll concentrations extracted using N,N-dimethylformamide (DMF)^94^. In brief, 100 mg of leaf lamina was transferred to a tube containing 3 ml of DMF and kept in the dark at 4 °C for 72 h. The absorbance of the solvent was then read at 647 and 664 nm using a spectrophotometer (Hitachi U-2000, Tokyo, Japan).

### RNA isolation and sequencing

Three leaves from control (0 mM NaCl) and salt-stressed (100 mM) plants were sampled 24 and 48 d after the beginning of experiment (hereafter referred to as S24 and S48, and C24 and C48 for salt-treated and control plants, respectively), corresponding to mid and final dates of the experiment.

One leaf per treatment was collected and separated into 2 parts. One portion was frozen with liquid nitrogen and then stored at −80 °C for RNA isolation, the other portion was used for other measurements.

Total RNA was isolated from leaves according to Giordani *et al*.^95^, followed by a DNAse I (Roche) digestion according to the manufacturer’s instructions, to remove genomic DNA contamination. Finally, RNA was purified by phenol/chloroform extraction and precipitated following standard procedures.

Twelve RNA-Seq libraries were generated using the TruSeq RNASeq Sample Prep kit, according to the manufacturer’s protocol (Illumina Inc., San Diego, CA, USA). Poly-A RNAs were isolated from the total RNA and chemically fragmented. The synthesis of first- and second-strand cDNA was followed by end-repair and 3′ adenylation. Adapters were ligated to the cDNA and fragments were gel-purified and enriched by PCR. Each library was quantified using a Bioanalyzer 2100 (Agilent Technologies, Santa Clara, CA, USA) and run on the Illumina HiSeq2000 (Illumina Inc.) using version 3 reagents.

Paired-ends read sequences were collected. The quality of the reads was checked using FastQC (v. 0.11.5)^96^ and the reads were trimmed with Trimmomatic (v. 0.33)^97^, cropping the first 15 bases and the last 10 bases of each read and removing adapter sequences in order to improve overall quality. Ribosomal RNA contaminant reads were removed by mapping to the DNA sequences of *Ficus* (18S, 5.8S, 28S partial) and *Prunus* (18S total and 5.8S partial) ribosomal RNAs from the NCBI repository (www.ncbi.nlm.nih.gov) using CLC Genomic Workbench version 9.5.3 (CLC-BIO, Aahrus, Denmark, hereafter called CLC) with default parameters. Unmapped reads were retained.

### Differential expression and gene ontology analysis

Trimmed reads were mapped onto coding sequences of the *F*. *carica* (cv. Horaishi) genome assembly (F. carica_assembly01^35^) using CLC with the following parameters: mismatch cost = 2, insertion/deletion cost = 3, length fraction = 0.8, similarity fraction = 0.9. CLC counted unique reads and discarded multi-reads, or distributed multi-reads at similar loci in proportion to the number of unique reads recorded. In the first case, the expression of genes having closely related paralogs would be underestimated. Hence, besides the unique reads we decided to also include reads that occurred up to ten times, a strategy designed to also allow a correct estimation of activity for paralogous genes^98^.

Raw counts of mapped reads were analysed with R statistical package edgeR^99^. Gene expression levels were calculated as reads per kilobase per million of mapped reads (RPKM)^98^. We retained genes with RPKM > 1 in at least 1library.

Pairwise comparison was performed between control and salt-stress libraries (C24-S24 and C48-S48). Resulting *p*-values were corrected with the False Discovery Rate^100^ and genes showing FDR < 0.05 were selected as significant.

The fold changes between treatments were considered significant when the expression values in a treatment were at least 2-fold higher or lower than in another, and this threshold was used to split genes into 2 groups: up-regulated or down-regulated.

Gene Ontology (GO) terms and enzyme codes were extracted from coding sequence annotations as supplied by Mori *et al*.^35^. GO-Slim^101^ was run to reduce the complexity of GO term distribution for gene class analysis. GO enrichment analysis with Fisher’s exact test on differential expressed genes (DEGs) versus the *F*. *carica* predicted transcriptome was performed using Blast2GO^101^, applying corrected *p*-values with FDR < 0.05. GO enrichment analysis was also performed on DEGs of early vs. late stage of saline stress, keeping over- and under-expressed genes separated. Enriched GO terms were summarized using REVIGO (http://revigo.irb.hr/) with “tiny” (cutoff value 0.4) allowed similarity parameters^102^.

Functional categorization of differentially expressed genes was performed using the MapMan tool^44^. MapMan BIN file for fig was obtained using Mercator (Blast_cutoff: 50 and IS_DNA^103^), comparing transcripts to classified proteins.

### Real Time PCR analyses

In order to validate RNA-seq analyses, the transcript levels of 5 genes randomly chosen amongst DEGs after 24 and 48 d of salinization were tested by quantitative Real-Time PCR analysis (qRT-PCR). Relative gene expression values were compared between non-treated samples (control) versus treated samples (salt-stressed). As housekeeping genes were selected an *actin-*encoding gene^35^ (ID code s00085g07448), an *alpha-tubulin*-encoding gene (ID code s00104g08427) and an rRNA 18S sequence (NCBI ID code LN999821), which showed constitutive expression profiles (Table S3). cDNA for qRT-PCR was obtained from 400 ng purified total RNA using the iScript cDNA synthesis kit (BioRad, Hercules, CA, USA) according to manufacturer’s protocol.

Primers were designed using Primer Express 3 software (Applied Biosystem, Foster City, CA, USA) (Table S3). qRT-PCR reactions (20 µl) were performed on 20 ng of cDNA using 200 nM of primers and 1X fast SYBR green Master Mix (Applied Biosystem) following the manufacturer’s instruction. PCR runs were carried out in a StepOne Real Time PCR System (Applied Biosystems).

qRT-PCR was performed on 3 biological and 3 technical replicates for each treatment. Relative abundance of transcripts was calculated using the 2−ΔΔCt method^104^. One-way analysis of variance (ANOVA) of expression level fold-changes was used to assess significant differences between control and treated samples of each gene. A reference actin-encoding gene was used as internal reference.

### Accession codes

Sequence reads of transcriptome sequencing have been deposited in the NCBI sequence read archive under bioproject accession code PRJNA508874.

## Supplementary information

Supplementary Materials
