# Supplementary material for: How an ancient, salt-tolerant fruit crop, Ficus carica L., copes with salinity: a transcriptome analysis
Source: Sci Rep. 2019 Feb 22;9:2561. doi: 10.1038/s41598-019-39114-4 (PMC6385202; doi:10.1038/s41598-019-39114-4)
Supplement: Supplementary file 1 — Supplementary Materials [file 41598_2019_39114_MOESM1_ESM.pdf]

How an ancient, salt-tolerant fruit crop, *Ficus carica* L., copes with salinity: a transcriptome analysis

SUPPLEMENTARY MATERIALS: Tables S1-S4, Figure S1-S6

Alberto Vangelisti, Liceth Solorzano Zambrano, Giovanni Caruso, Desiré Macheda, Rodolfo Bernardi, Gabriele Usai, Flavia Mascagni, Tommaso Giordani, Riccardo Gucci, Andrea Cavallini & Lucia Natali

Department of Agriculture, Food, and Environment, University of Pisa, Via del Borghetto 80, I-56124, Pisa, Italy. Correspondence and requests for materials should be addressed to L.N. (email:lucia.natali@unipi.it)

Table S1. Number of raw and trimmed Illumina paired-end reads used in the experiments and number of reads matching to the *F. carica* cv. Horaishi transcriptome (Mori et al. 2017) for each library. c24= control leaves 24 days after treatment, c48= control leaves 48 days after treatment, s24= salt stress leaves 24 days after treatment, s48= salt stress leaves 48 days after treatment.

Library nr. and treatment	Number of raw reads	Number of reads after trimming	Number of mapped reads onto*F. carica* transcriptome	Percentage of mapped reads onto*F. carica* transcriptome
1 (c24)	20,466,392	19,656,486	14,493,872	73.74
2 (c24)	13,598,862	13,207,584	9,825,343	74.39
3 (c24)	15,094,998	14,614,598	10,462,566	71.59
4 (s24)	16,451,494	15,787,836	11,586,185	73.39
5 (s24)	14,988,358	14,479,386	10,686,045	73.8
6 (s24)	14,385,156	13,820,318	10,199,528	73.8
7 (c48)	61,639,892	58,183,090	42,888,532	73.71
8 (c48)	21,256,610	20,130,780	15,137,335	75.19
9 (c48)	19,393,686	18,677,070	14,172,766	75.88
10 (s48)	15,743,236	15,399,566	11,615,583	75.43
11 (s48)	21,000,134	20,236,072	15,129,672	74.77
12 (s48)	14,772,974	14,346,828	10,551,384	73.55

Table S2. Differentially expressed genes (DEGs) over and under expressed in response to salt after 24 (D24) and 48 (D48) days of treatment. Blue-evidenced DEGs are the 100 highest fold change per class. LogFC= log fold change, FDR= adjusted P value with false discovery rate.

genes	logFC	FDR	product	description	Expression	Time point
s00080g07240	6,916	0,000	cytochrome P450 82A3	Cytochrome P450 82A3 [Morus notabilis] gi|587919895|gb|EXC07349.1|	Over Expressed	D24
s00025g03163	6,853	0,005	aluminum-activated malate transporter 12	Aluminum-activated malate transporter 12 [Morus notabilis]	Over Expressed	D24
s00876g24803	6,588	0,004	-	PREDICTED: extensin-2 isoform X2 [Vigna radiata var. radiata]	Over Expressed	D24
s00169g11264	6,482	0,000	-	transmembrane protein, putative [Medicago truncatula]	Over Expressed	D24
s11940g33512	5,455	0,004	TFL1-like protein	TFL1-like protein [Fragaria vesca]	Over Expressed	D24
s00014g02049	5,432	0,012	-	PREDICTED: beta-fructofuranosidase, cell wall isozyme-like [Cicer arietinum]	Over Expressed	D24
s00928g25376	5,427	0,014	-	***** No hits found ******	Over Expressed	D24
s00097g08100	5,244	0,031	-	***** No hits found ******	Over Expressed	D24
s00609g21531	5,183	0,006	-	***** No hits found ******	Over Expressed	D24
s03038g31142	5,036	0,010	-	Copia protein [Morus notabilis] gi|587885647|gb|EXB74504.1|	Over Expressed	D24
s00694g22691	4,873	0,020	-	### Not characterized ###:hypothetical protein PRUPE_ppa023320mg [Prunus persica]	Over Expressed	D24
s00188g11944	4,869	0,019	-	***** No hits found ******	Over Expressed	D24
s00283g14790	4,686	0,027	-	***** No hits found ******	Over Expressed	D24
s00080g07229	4,678	0,037	disease resistance-responsive family protein	Disease resistance-responsive family protein [Theobroma cacao]	Over Expressed	D24
s00733g23197	4,474	0,048	-	***** No hits found ******	Over Expressed	D24
s21097g35200	4,216	0,014	disease resistance protein RPM1	Disease resistance protein RPM1 [Morus notabilis]	Over Expressed	D24
s00051g05315	3,889	0,000	-	PREDICTED: protein NRT1/ PTR FAMILY 5.6-like [Prunus mume]	Over Expressed	D24
s18818g34833	3,690	0,025	-	Agamous-like MADS-box protein AGL8 [Morus notabilis]	Over Expressed	D24
s00306g15416	3,339	0,001	-	PREDICTED: cucumisin-like [Vitis vinifera]	Over Expressed	D24
s03704g31357	3,086	0,025	-	PREDICTED: random slug protein 5 [Cucumis melo]	Over Expressed	D24
s00785g23824	3,081	0,008	isoflavone 2'-hydroxylase	Isoflavone 2'-hydroxylase [Morus notabilis]	Over Expressed	D24
s03458g31279	3,004	0,006	-	### Not characterized ###:hypothetical protein L484_012677 [Morus notabilis]	Over Expressed	D24

s00620g21689	3,001	0,000	transcription factor bHLH10-like	PREDICTED: transcription factor bHLH10-like [Pyrus x bretschneideri] gi|694397183|ref|XP_009373851.1|	Over Expressed	D24
s00018g02534	2,807	0,015	-	PREDICTED: putative late blight resistance protein homolog R1B-19 [Populus euphratica] gi|743852780|ref|XP_011029353.1|	Over Expressed	D24
s00748g23382	2,783	0,003	ethylene-responsive transcription factor WRI1	Ethylene-responsive transcription factor WRI1 [Morus notabilis]	Over Expressed	D24
s00591g21218	2,775	0,003	similar to L484_004420	### Not characterized ###:hypothetical protein L484_004420 [Morus notabilis]	Over Expressed	D24
s00205g12522	2,726	0,031	-	***** No hits found ******	Over Expressed	D24
s00593g21248	2,629	0,043	-	Putative F-box/LRR-repeat protein [Morus notabilis]	Over Expressed	D24
s00620g21688	2,622	0,028	similar to L484_023851	### Not characterized ###:hypothetical protein L484_023851 [Morus notabilis]	Over Expressed	D24
s00153g10646	2,571	0,000	B3 domain-containing transcription factor VRN1	B3 domain-containing transcription factor VRN1 [Morus notabilis]	Over Expressed	D24
s00023g03041	2,566	0,026	-	### Not characterized ###:hypothetical protein L484_012049 [Morus notabilis]	Over Expressed	D24
s00111g08780	2,496	0,000	C globular stage	C globular stage, putative [Theobroma cacao]	Over Expressed	D24
s07100g32310	2,496	0,035	peptide/nitrate transporter	putative peptide/nitrate transporter [Morus notabilis]	Over Expressed	D24
s00364g16843	2,492	0,000	F-box protein	F-box protein [Morus notabilis] gi|587851439|gb|EXB41588.1|	Over Expressed	D24
s00094g07968	2,386	0,037	-	PREDICTED: protein LURP-one-related 4 [Vitis vinifera]	Over Expressed	D24
s00043g04670	2,366	0,012	mtN26	MtN26 [Medicago truncatula] gi|657404281|gb|KEH43026.1| MtN26 [Medicago truncatula]	Over Expressed	D24
s00003g00605	2,239	0,002	-	### Not characterized ###:hypothetical protein L484_007652 [Morus notabilis]	Over Expressed	D24
s14537g34056	2,235	0,012	uncharacterized protein LOC105969824	### Not characterized ###:PREDICTED: uncharacterized protein LOC105969824 [Erythranthe guttata]	Over Expressed	D24
s00041g04549	2,212	0,004	sulfate transporter 3.4	putative sulfate transporter 3.4 [Morus notabilis]	Over Expressed	D24
s00247g13804	2,209	0,011	E3 ubiquitin-protein ligase RHA2A	E3 ubiquitin-protein ligase RHA2A [Morus notabilis]	Over Expressed	D24
s00622g21717	2,193	0,024	-	***** No hits found ******	Over Expressed	D24

s00114g08941	2,189	0,000	DNA ligase	DNA ligase [Gossypium arboreum]	Over Expressed	D24
s17600g34609	2,181	0,017	disease resistance protein RPM1	Disease resistance protein RPM1 [Morus notabilis]	Over Expressed	D24
s00023g02999	2,095	0,002	BTB/POZ domain-containing protein POB1-like	PREDICTED: BTB/POZ domain-containing protein POB1-like [Vitis vinifera]	Over Expressed	D24
s01125g26995	2,034	0,030	-	### Not characterized ###:hypothetical protein L484_021458 [Morus notabilis]	Over Expressed	D24
s00143g10212	2,015	0,042	-	***** No hits found ******	Over Expressed	D24
s00679g22491	2,002	0,000	nuclear transcription factor Y subunit A-9	Nuclear transcription factor Y subunit A-9 [Morus notabilis]	Over Expressed	D24
s00857g24607	1,981	0,001	uncharacterized protein LOC105640359	### Not characterized ###:PREDICTED: uncharacterized protein LOC105640359 [Jatropha curcas]	Over Expressed	D24
s00624g21744	1,979	0,008	-	### Not characterized ###:hypothetical protein VITISV_041073 [Vitis vinifera]	Over Expressed	D24
s00508g19829	1,973	0,000	-	### Not characterized ###:PREDICTED: uncharacterized protein LOC104881291 [Vitis vinifera]	Over Expressed	D24
s00001g00053	1,959	0,005	-	PREDICTED: uncharacterized mitochondrial protein AtMg00810-like [Glycine max]	Over Expressed	D24
s00016g02282	1,950	0,000	-	PREDICTED: probable root meristem growth factor 8 [Prunus mume]	Over Expressed	D24
s02934g31106	1,935	0,000	-	***** No hits found ******	Over Expressed	D24
s00614g21590	1,922	0,000	-	Auxin-induced protein 5NG4 [Morus notabilis]	Over Expressed	D24
s00100g08254	1,904	0,000	phosphoglucomutase / glucose phosphomutase	Phosphoglucomutase / glucose phosphomutase, putative [Theobroma cacao] gi|508709124|gb|EOY01021.1|	Over Expressed	D24
s19544g34955	1,901	0,010	-	PREDICTED: putative methyltransferase C9orf114 [Jatropha curcas]	Over Expressed	D24
s00080g07239	1,883	0,027	cytochrome P450 82A3	Cytochrome P450 82A3 [Morus notabilis] gi|587919895|gb|EXC07349.1|	Over Expressed	D24
s00419g18056	1,848	0,043	MLO-like protein 2	PREDICTED: MLO-like protein 2, partial [Prunus mume]	Over Expressed	D24
s00149g10484	1,799	0,003	inorganic phosphate transporter 1-7	putative inorganic phosphate transporter 1-7 [Morus notabilis]	Over Expressed	D24
s00261g14202	1,741	0,039	acyl-protein thioesterase 2-like	PREDICTED: acyl-protein thioesterase 2-like [Populus euphratica]	Over Expressed	D24
s00147g10413	1,733	0,000	granule-bound starch synthase 1	Granule-bound starch synthase 1 [Morus notabilis]	Over Expressed	D24

s00145g10292	1,723	0,001	-	### Not characterized ###:hypothetical protein L484_025067 [Morus notabilis]	Over Expressed	D24
s01806g30007	1,718	0,046	S-adenosylmethionine-dependent methyltransferase	putative S-adenosylmethionine-dependent methyltransferase [Morus notabilis] gi|587935268|gb|EXC22151.1|	Over Expressed	D24
s00129g09612	1,717	0,000	chaperonin 60 subunit beta 4	Chaperonin 60 subunit beta 4 [Morus notabilis]	Over Expressed	D24
s00067g06394	1,712	0,038	serine/threonine-protein kinase	Serine/threonine-protein kinase [Morus notabilis]	Over Expressed	D24
s00281g14755	1,702	0,000	adagio protein 3	Adagio protein 3 [Morus notabilis] gi|587841729|gb|EXB32326.1|	Over Expressed	D24
s00712g22909	1,700	0,007	serine/threonine-protein phosphatase 6 regulatory ankyrin repeat subunit B-like	PREDICTED: serine/threonine-protein phosphatase 6 regulatory ankyrin repeat subunit B-like [Malus domestica]	Over Expressed	D24
s00082g07320	1,700	0,040	myb-related protein MYBAS1	Myb-related protein MYBAS1 [Morus notabilis]	Over Expressed	D24
s20456g35106	1,697	0,018	-	***** No hits found ******	Over Expressed	D24
s25528g35814	1,695	0,008	-	***** No hits found ******	Over Expressed	D24
s00065g06255	1,688	0,001	adenylate isopentenyltransferase 3	Adenylate isopentenyltransferase 3 [Morus notabilis]	Over Expressed	D24
s00096g08061	1,676	0,009	cytochrome P450 isoform 1	Cytochrome P450 isoform 1 [Theobroma cacao]	Over Expressed	D24
s00080g07242	1,667	0,002	cytochrome P450 82A3	Cytochrome P450 82A3 [Morus notabilis] gi|587919895|gb|EXC07349.1|	Over Expressed	D24
s00319g15780	1,654	0,001	alpha-1,4 glucan phosphorylase L isozyme	Alpha-1,4 glucan phosphorylase L isozyme [Morus notabilis]	Over Expressed	D24
s00673g22420	1,633	0,001	pentatricopeptide repeat-containing protein At1g12700, mitochondrial	PREDICTED: putative pentatricopeptide repeat-containing protein At1g12700, mitochondrial [Malus domestica]	Over Expressed	D24
s00429g18275	1,633	0,001	anion transporter 5	PREDICTED: probable anion transporter 5 [Gossypium raimondii]	Over Expressed	D24
s00112g08810	1,625	0,001	-	PREDICTED: Fanconi anemia group I protein [Prunus mume]	Over Expressed	D24
s00192g12092	1,617	0,017	LRR receptor-like serine/threonine-protein kinase	putative LRR receptor-like serine/threonine-protein kinase [Morus notabilis] gi|587849092|gb|EXB39332.1|	Over Expressed	D24
s00405g17761	1,614	0,000	similar to L484_020058	### Not characterized ###:hypothetical protein L484_020058 [Morus notabilis]	Over Expressed	D24
s02020g30426	1,592	0,000	-	putative Ty1-copia-like retrotransposon [Cercis chinensis]	Over Expressed	D24
s00069g06496	1,588	0,008	-	Chloroplastic group IIA intron splicing facilitator CRS1, chloroplastic [Glycine soja]	Over Expressed	D24

s00191g12052	1,581	0,000	26S proteasome non-ATPase regulatory subunit 10	26S proteasome non-ATPase regulatory subunit 10 [Morus notabilis]	Over Expressed	D24
s00477g19233	1,577	0,001	similar to L484_013265	### Not characterized ###:hypothetical protein L484_013265 [Morus notabilis]	Over Expressed	D24
s00005g00946	1,572	0,001	receptor-like cytosolic serine/threonine-protein kinase RBK2	Receptor-like cytosolic serine/threonine-protein kinase RBK2 [Morus notabilis] gi|587918190|gb|EXC05707.1|	Over Expressed	D24
s00454g18802	1,563	0,002	-	### Not characterized ###:hypothetical protein L484_026905 [Morus notabilis]	Over Expressed	D24
s07428g32405	1,559	0,007	similar to L484_017568	### Not characterized ###:hypothetical protein L484_017568 [Morus notabilis]	Over Expressed	D24
s01287g28013	1,556	0,000	aldehyde dehydrogenase family 2 member C4	Aldehyde dehydrogenase family 2 member C4 [Morus notabilis]	Over Expressed	D24
s00041g04572	1,555	0,003	mitochondrial carnitine/acylcarnitine carrier protein CACL	Mitochondrial carnitine/acylcarnitine carrier protein CACL [Morus notabilis] gi|587885639|gb|EXB74496.1|	Over Expressed	D24
s00260g14182	1,540	0,008	mitochondrial rRNA methyltransferase	Mitochondrial rRNA methyltransferase [Morus notabilis]	Over Expressed	D24
s00086g07516	1,533	0,025	-	putative membrane-associated kinase regulator 6 -like protein [Gossypium arboreum]	Over Expressed	D24
s00033g03875	1,521	0,035	acyl-protein thioesterase 2	Acyl-protein thioesterase 2 [Morus notabilis]	Over Expressed	D24
s00014g02114	1,520	0,041	nodulation-signaling pathway 1 protein isoform X2	PREDICTED: nodulation-signaling pathway 1 protein isoform X1 [Vitis vinifera] gi|731401830|ref|XP_010654436.1|	Over Expressed	D24
s01090g26759	1,502	0,000	J domain-containing protein requiredfor chloroplast accumulation response 1 isoform X1	PREDICTED: J domain-containing protein requiredfor chloroplast accumulation response 1 isoform X1 [Prunus mume]	Over Expressed	D24
s00183g11749	1,501	0,024	cation/H(+) antiporter 15	Cation/H(+) antiporter 15 [Morus notabilis]	Over Expressed	D24
s01108g26885	1,500	0,000	(S)-N-methylcoclaurine 3'-hydroxylase isozyme 2	(S)-N-methylcoclaurine 3'-hydroxylase isozyme 2 [Morus notabilis]	Over Expressed	D24
s01124g26988	1,496	0,013	tubby-like protein 8	PREDICTED: tubby-like protein 8 [Prunus mume]	Over Expressed	D24
s00944g25535	1,489	0,032	-	***** No hits found ******	Over Expressed	D24
s01820g30040	1,487	0,014	-	PREDICTED: protein NUCLEAR FUSION DEFECTIVE 4-like [Fragaria vesca subsp. vesca]	Over Expressed	D24
s00014g02018	1,482	0,011	-	PREDICTED: ABC transporter C family member 8-like [Prunus mume]	Over Expressed	D24

s00006g01093	1,481	0,000	glucose-6-phosphate 1-epimerase	Putative glucose-6-phosphate 1-epimerase [Morus notabilis]	Over Expressed	D24
s00897g25053	1,468	0,004	-	***** No hits found ******	Over Expressed	D24
s00896g25044	1,459	0,000	ribonuclease J	Ribonuclease J [Morus notabilis] gi|587916461|gb|EXC04124.1|	Over Expressed	D24
s00008g01301	1,458	0,004	4-alpha-glucanotransferase	4-alpha-glucanotransferase [Morus notabilis]	Over Expressed	D24
s00111g08760	1,454	0,017	multidrug resistance protein	Putative multidrug resistance protein [Morus notabilis]	Over Expressed	D24
s06767g32224	1,446	0,004	-	### Not characterized ###:hypothetical protein L484_024613 [Morus notabilis]	Over Expressed	D24
s00112g08808	1,441	0,002	uncharacterized protein LOC100240766	### Not characterized ###:PREDICTED: uncharacterized protein LOC100240766 [Vitis vinifera]	Over Expressed	D24
s00038g04301	1,425	0,006	leucine-rich repeat receptor protein kinase EXS	Leucine-rich repeat receptor protein kinase EXS [Morus notabilis]	Over Expressed	D24
s03029g31139	1,420	0,005	G-type lectin S-receptor-like serine/threonine-protein kinase At1g61500	PREDICTED: G-type lectin S-receptor-like serine/threonine-protein kinase At1g61500 [Prunus mume]	Over Expressed	D24
s15592g34233	1,419	0,040	-	F-box/FBD/LRR-repeat protein [Morus notabilis]	Over Expressed	D24
s00248g13814	1,415	0,008	E3 ubiquitin-protein ligase MARCH1	PREDICTED: E3 ubiquitin-protein ligase MARCH1 [Populus euphratica]	Over Expressed	D24
s00440g18488	1,406	0,001	glycosyltransferase	putative glycosyltransferase [Morus notabilis]	Over Expressed	D24
s00310g15542	1,400	0,005	exonuclease 1	PREDICTED: exonuclease 1 [Pyrus x bretschneideri]	Over Expressed	D24
s01966g30340	1,398	0,022	-	Receptor-like protein 12 [Morus notabilis]	Over Expressed	D24
s00361g16769	1,387	0,002	G-type lectin S-receptor-like serine/threonine-protein kinase	G-type lectin S-receptor-like serine/threonine-protein kinase [Morus notabilis] gi|587924278|gb|EXC11583.1|	Over Expressed	D24
s00052g05353	1,373	0,001	transcription factor bHLH47	PREDICTED: transcription factor bHLH47 [Prunus mume]	Over Expressed	D24
s10596g33210	1,367	0,042	-	Transcription factor TGA7 [Morus notabilis]	Over Expressed	D24
s00248g13837	1,364	0,000	UDP-glycosyltransferase 76E2	UDP-glycosyltransferase 76E2 [Morus notabilis]	Over Expressed	D24
s00331g16057	1,363	0,003	serine/threonine-protein kinase SAPK3 isoform 2	Serine/threonine-protein kinase SAPK3 isoform 2, partial [Theobroma cacao] gi|508776903|gb|EOY24159.1|	Over Expressed	D24
s00423g18151	1,358	0,002	similar to L484_022081	### Not characterized ###:hypothetical protein L484_022081 [Morus notabilis]	Over Expressed	D24

s00376g17141	1,347	0,041	zinc finger protein CONSTANS-LIKE 5	Zinc finger protein CONSTANS-LIKE 5 [Morus notabilis]	Over Expressed	D24
s00154g10696	1,339	0,029	scarecrow-like protein 4	PREDICTED: scarecrow-like protein 4 [Jatropha curcas]	Over Expressed	D24
s00009g01450	1,337	0,029	-	***** No hits found ******	Over Expressed	D24
s00183g11762	1,335	0,002	-	PREDICTED: superoxide dismutase 1 copper chaperone-like [Camelina sativa]	Over Expressed	D24
s01395g28577	1,334	0,028	metal tolerance protein 10	Metal tolerance protein 10 [Morus notabilis]	Over Expressed	D24
s01692g29697	1,332	0,002	-	Retrovirus-related Pol polyprotein from transposon TNT 1-94 [Morus notabilis] gi|587900817|gb|EXB89110.1|	Over Expressed	D24
s00138g09977	1,329	0,002	DEAD-box ATP-dependent RNA helicase 22	DEAD-box ATP-dependent RNA helicase 22 [Morus notabilis]	Over Expressed	D24
s00206g12556	1,327	0,029	vacuolar amino acid transporter YPQ1	PREDICTED: probable vacuolar amino acid transporter YPQ1 [Prunus mume]	Over Expressed	D24
s00114g08945	1,326	0,048	-	F-box [Gossypium arboreum]	Over Expressed	D24
s00090g07730	1,324	0,001	vignain-like	PREDICTED: vignain-like [Prunus mume]	Over Expressed	D24
s00157g10783	1,323	0,000	-	### Not characterized ###:hypothetical protein JCGZ_04599 [Jatropha curcas]	Over Expressed	D24
s04644g31640	1,322	0,020	similar to L484_024613	### Not characterized ###:hypothetical protein L484_024613 [Morus notabilis]	Over Expressed	D24
s00037g04222	1,320	0,035	-	***** No hits found ******	Over Expressed	D24
s01143g27128	1,307	0,002	RNA polymerase sigma factor rpoD	RNA polymerase sigma factor rpoD [Morus notabilis]	Over Expressed	D24
s00016g02290	1,305	0,047	cytochrome P450 734A1	Cytochrome P450 734A1 [Morus notabilis] gi|587854862|gb|EXB44887.1|	Over Expressed	D24
s01251g27836	1,301	0,018	-	***** No hits found ******	Over Expressed	D24
s01749g29881	1,291	0,002	purine permease 9	PREDICTED: probable purine permease 9 [Prunus mume]	Over Expressed	D24
s00278g14663	1,281	0,000	nucleic acid-binding, OB-fold-like protein isoform 1	Nucleic acid-binding, OB-fold-like protein isoform 1 [Theobroma cacao] gi|508775153|gb|EOY22409.1|	Over Expressed	D24
s00001g00034	1,275	0,037	-	PREDICTED: nucleolin 2-like isoform X1 [Citrus sinensis]	Over Expressed	D24
s00059g05902	1,270	0,000	O-fucosyltransferase family protein	O-fucosyltransferase family protein [Theobroma cacao]	Over Expressed	D24
s00207g12589	1,265	0,001	boron transporter 1	PREDICTED: boron transporter 1 [Cucumis melo]	Over Expressed	D24
s00437g18426	1,258	0,042	-	Cysteine-rich RLK (RECEPTOR-like protein kinase) 8 [Theobroma cacao] gi|508718075|gb|EOY09972.1|	Over Expressed	D24

s00045g04848	1,255	0,000	mitochondrial transcription termination factor family protein	Mitochondrial transcription termination factor family protein [Theobroma cacao] gi|508706657|gb|EOX98553.1|	Over Expressed	D24
s00167g11170	1,253	0,022	RING-H2 zinc finger protein RHA4a	RING-H2 zinc finger protein RHA4a [Morus notabilis]	Over Expressed	D24
s00621g21711	1,251	0,039	scarecrow-like transcription factor PAT1	PREDICTED: scarecrow-like transcription factor PAT1 [Prunus mume]	Over Expressed	D24
s00014g02014	1,251	0,045	ABC transporter C family member 8	ABC transporter C family member 8 [Morus notabilis]	Over Expressed	D24
s01419g28694	1,245	0,002	prostaglandin E synthase 2	Prostaglandin E synthase 2 [Morus notabilis]	Over Expressed	D24
s01387g28550	1,236	0,001	1,4-alpha-glucan-branching enzyme	1,4-alpha-glucan-branching enzyme [Morus notabilis]	Over Expressed	D24
s00444g18584	1,210	0,031	similar to L484_000375	### Not characterized ###:hypothetical protein L484_000375 [Morus notabilis]	Over Expressed	D24
s00123g09352	1,207	0,013	uncharacterized protein LOC103950761	### Not characterized ###:PREDICTED: uncharacterized protein LOC103950761 [Pyrus x bretschneideri]	Over Expressed	D24
s00696g22715	1,202	0,000	disease resistance protein RPM1-like	PREDICTED: disease resistance protein RPM1-like [Pyrus x bretschneideri]	Over Expressed	D24
s00137g09943	1,197	0,029	chaperone protein ClpB 1	Chaperone protein ClpB 1 [Morus notabilis]	Over Expressed	D24
s01012g26133	1,196	0,001	-	### Not characterized ###:PREDICTED: uncharacterized protein LOC102629445 [Citrus sinensis]	Over Expressed	D24
s00704g22801	1,195	0,021	gamma-irradiation and mitomycin c induced 1, putative isoform 1	Gamma-irradiation andmitomycinc induced 1, putative isoform 1 [Theobroma cacao] gi|508776308|gb|EOY23564.1|	Over Expressed	D24
s05000g31744	1,195	0,048	-	### Not characterized ###:hypothetical protein L484_012525 [Morus notabilis]	Over Expressed	D24
s00114g08910	1,194	0,002	regulator of Vps4 activity inthe MVB pathway protein isoform 1	Regulator of Vps4 activityin the MVB pathway protein isoform 1 [Theobroma cacao] gi|508698821|gb|EOX90717.1|	Over Expressed	D24
s01840g30080	1,192	0,015	nuclear transport factor 2 family protein	Nuclear transport factor 2 family protein [Theobroma cacao]	Over Expressed	D24
s00248g13812	1,190	0,042	tubulin beta-1 chain	Tubulin beta-1 chain [Morus notabilis] gi|587839844|gb|EXB30492.1|	Over Expressed	D24
s00698g22740	1,184	0,004	zinc transporter 11	Zinc transporter 11 [Morus notabilis] gi|587874120|gb|EXB63273.1|	Over Expressed	D24
s00090g07756	1,180	0,008	two-component response regulator	Two-component response regulator [Morus notabilis]	Over Expressed	D24

s00288g14948	1,165	0,001	-	***** No hits found ******	Over Expressed	D24
s00333g16102	1,157	0,000	-	***** No hits found ******	Over Expressed	D24
s00145g10309	1,156	0,022	similar to L484_025078	### Not characterized ###:hypothetical protein L484_025078 [Morus notabilis]	Over Expressed	D24
s00017g02349	1,154	0,004	tropinone reductase-like protein	Tropinone reductase-like protein [Morus notabilis]	Over Expressed	D24
s00047g05006	1,149	0,006	RING fingerand CHY zinc finger domain-containing protein 1 isoform X1	PREDICTED: RING fingerand CHY zinc finger domain-containing protein 1 isoform X1 [Pyrus x bretschneideri]	Over Expressed	D24
s00911g25205	1,135	0,033	transducin/WD40 repeat-like superfamily protein	Transducin/WD40 repeat-like superfamily protein, putative [Theobroma cacao] gi|508780239|gb|EOY27495.1|	Over Expressed	D24
s00969g25764	1,135	0,027	UPF0695 membrane protein	UPF0695 membrane protein [Morus notabilis]	Over Expressed	D24
s00154g10681	1,133	0,000	isoflavone reductase-like protein	PREDICTED: isoflavone reductase-like protein [Glycine max]	Over Expressed	D24
s00051g05310	1,129	0,036	-	PREDICTED: protein DA1-related 2-like isoform X2 [Nicotiana tomentosiformis]	Over Expressed	D24
s00183g11748	1,129	0,007	cation/H(+) antiporter 15	Cation/H(+) antiporter 15 [Morus notabilis]	Over Expressed	D24
s00184g11792	1,127	0,014	-	PREDICTED: LOW QUALITY PROTEIN: protein S-acyltransferase 18 [Prunus mume]	Over Expressed	D24
s00003g00556	1,125	0,027	arsenical pump-driving ATPase	Putative arsenical pump-driving ATPase [Morus notabilis]	Over Expressed	D24
s00293g15070	1,122	0,000	senescence-associated family protein	senescence-associated family protein [Populus trichocarpa]	Over Expressed	D24
s01409g28655	1,120	0,026	DNA polymerase III polC-type	DNA polymerase III polC-type [Morus notabilis]	Over Expressed	D24
s00079g07115	1,116	0,047	-	***** No hits found ******	Over Expressed	D24
s01293g28041	1,110	0,022	-	***** No hits found ******	Over Expressed	D24
s00264g14303	1,103	0,008	neurofilament heavy polypeptide-like	PREDICTED: neurofilament heavy polypeptide-like [Jaculus jaculus]	Over Expressed	D24
s00092g07855	1,103	0,000	chlorophyllase-2	Chlorophyllase-2 [Morus notabilis] gi|587861296|gb|EXB51153.1|	Over Expressed	D24
s00010g01628	1,100	0,012	LRR receptor-like serine/threonine-protein kinase HSL2	LRR receptor-like serine/threonine-protein kinase HSL2 [Morus notabilis] gi|587916185|gb|EXC03884.1|	Over Expressed	D24
s00604g21431	1,098	0,021	pentatricopeptide repeat-containing protein At1g62680, mitochondrial-like	PREDICTED: pentatricopeptide repeat-containing protein At1g62680, mitochondrial-like [Malus domestica]	Over Expressed	D24
s00120g09223	1,094	0,000	methyltransferase C9orf114	PREDICTED: putative methyltransferase C9orf114 [Vitis vinifera]	Over Expressed	D24
s00092g07854	1,092	0,000	DNA-directed RNA polymerase III subunit RPC9	DNA-directed RNA polymerase III subunit RPC9 [Morus notabilis]	Over Expressed	D24

s00001g00054	1,088	0,005	uncharacterized protein LOC102627405	### Not characterized ###:PREDICTED: uncharacterized protein LOC102627405 [Citrus sinensis]	Over Expressed	D24
s00670g22389	1,086	0,000	two-component response regulator	Two-component response regulator [Morus notabilis]	Over Expressed	D24
s00749g23390	1,085	0,008	pentatricopeptide repeat-containing protein At1g30610, chloroplastic	PREDICTED: pentatricopeptide repeat-containing protein At1g30610, chloroplastic [Prunus mume]	Over Expressed	D24
s00603g21421	1,085	0,000	ATP-dependent zinc metalloprotease FtsH	ATP-dependent zinc metalloprotease FtsH [Morus notabilis]	Over Expressed	D24
s00948g25580	1,080	0,012	phosphoenolpyruvate carboxylase 2	Phosphoenolpyruvate carboxylase 2 [Morus notabilis]	Over Expressed	D24
s00237g13551	1,079	0,019	two-component response regulator-like protein	Two-component response regulator-like protein [Morus notabilis]	Over Expressed	D24
s00331g16049	1,075	0,027	methyltransferase TARBP1	putative methyltransferase TARBP1 [Morus notabilis]	Over Expressed	D24
s00179g11613	1,074	0,000	MATE efflux family protein 1-like	PREDICTED: MATE efflux family protein 1-like [Prunus mume]	Over Expressed	D24
s00079g07173	1,073	0,000	sodium-dependent transporter yocS	putative sodium-dependent transporter yocS [Morus notabilis]	Over Expressed	D24
s00033g03917	1,066	0,007	aspartate, glycine, lysineand serine-rich protein-like	PREDICTED: aspartate, glycine, lysineand serine-rich protein-like [Prunus mume]	Over Expressed	D24
s00063g06163	1,065	0,003	IAA-amino acid hydrolase	IAA-amino acid hydrolase [Morus notabilis]	Over Expressed	D24
s00978g25840	1,062	0,004	-	***** No hits found ******	Over Expressed	D24
s00786g23838	1,060	0,004	subtilisin-like protease	Subtilisin-like protease [Morus notabilis]	Over Expressed	D24
s00180g11623	1,060	0,007	UDP-N-acetylmuramoyl-L-alanyl-D-glutamate--2, 6-diaminopimelate ligase	UDP-N-acetylmuramoyl-L-alanyl-D-glutamate--2, 6-diaminopimelate ligase [Morus notabilis] gi|587846902|gb|EXB37342.1|	Over Expressed	D24
s00147g10412	1,053	0,000	glucan endo-1,3-beta-glucosidase 11	Glucan endo-1,3-beta-glucosidase 11 [Morus notabilis]	Over Expressed	D24
s00057g05768	1,051	0,000	uncharacterized membrane protein	Uncharacterized membrane protein [Morus notabilis]	Over Expressed	D24
s00092g07866	1,050	0,003	-	PREDICTED: protein tesmin/TSO1-like CXC 2 isoform X2 [Prunus mume]	Over Expressed	D24
s00032g03841	1,048	0,020	similar to L484_017966	### Not characterized ###:hypothetical protein L484_017966 [Morus notabilis]	Over Expressed	D24
s01251g27834	1,046	0,000	-	***** No hits found ******	Over Expressed	D24

s00051g05309	1,044	0,049	-	PREDICTED: protein DA1-related 2-like [Pyrus x bretschneideri]	Over Expressed	D24
s01287g28012	1,043	0,000	aldehyde dehydrogenase family 2 member C4	Aldehyde dehydrogenase family 2 member C4 [Morus notabilis]	Over Expressed	D24
s08562g32722	1,038	0,012	-	Putative quinone-oxidoreductase-like protein [Morus notabilis]	Over Expressed	D24
s01021g26203	1,035	0,001	histidinol-phosphate aminotransferase	Histidinol-phosphate aminotransferase [Morus notabilis]	Over Expressed	D24
s01003g26059	1,034	0,047	cysteine-rich RLK (RECEPTOR-like protein kinase) 8	Cysteine-rich RLK (RECEPTOR-like protein kinase) 8 [Theobroma cacao]	Over Expressed	D24
s17598g34608	1,033	0,046	-	Alliin lyase precursor, putative [Ricinus communis]	Over Expressed	D24
s01998g30387	1,029	0,005	RNA helicase SDE3	PREDICTED: probable RNA helicase SDE3 [Populus euphratica]	Over Expressed	D24
s00745g23345	1,026	0,036	flowering locus T family protein	flowering locus T family protein [Populus trichocarpa]	Over Expressed	D24
s00204g12512	1,025	0,040	similar to L484_000425	### Not characterized ###:hypothetical protein L484_000425 [Morus notabilis]	Over Expressed	D24
s00052g05364	1,024	0,028	pentatricopeptide repeat-containing protein At1g74600, chloroplastic	PREDICTED: pentatricopeptide repeat-containing protein At1g74600, chloroplastic [Prunus mume]	Over Expressed	D24
s00407g17827	1,023	0,000	-	PREDICTED: protein TRANSPARENT TESTA 12-like [Gossypium raimondii]	Over Expressed	D24
s00040g04423	1,023	0,016	-	***** No hits found ******	Over Expressed	D24
s01229g27711	1,015	0,018	-	PREDICTED: pentatricopeptide repeat-containing protein At1g62720-like [Pyrus x bretschneideri]	Over Expressed	D24
s00661g22285	1,014	0,000	anthocyanidin 5,3-O-glucosyltransferase	Anthocyanidin 5,3-O-glucosyltransferase [Morus notabilis]	Over Expressed	D24
s00242g13683	1,014	0,033	pentatricopeptide repeat-containing protein At4g39952, mitochondrial	PREDICTED: pentatricopeptide repeat-containing protein At4g39952, mitochondrial [Prunus mume]	Over Expressed	D24
s01107g26873	1,014	0,003	pentatricopeptide repeat-containing protein At5g08305	PREDICTED: pentatricopeptide repeat-containing protein At5g08305 [Prunus mume]	Over Expressed	D24
s00312g15573	1,014	0,008	1-aminocyclopropane-1-carboxylate oxidase homolog 1-like	PREDICTED: 1-aminocyclopropane-1-carboxylate oxidase homolog 1-like [Nicotiana tomentosiformis]	Over Expressed	D24
s00333g16101	1,013	0,008	chloroplastic group IIA intron splicing facilitator CRS1	Chloroplastic group IIA intron splicing facilitator CRS1 [Morus notabilis] gi|587933540|gb|EXC20503.1|	Over Expressed	D24

s00033g03906	1,010	0,024	inactive rhomboid protein 1	Inactive rhomboid protein 1 [Morus notabilis]	Over Expressed	D24
s01478g28934	1,009	0,030	TBC1 domain family member 13	TBC1 domain family member 13 [Morus notabilis]	Over Expressed	D24
s00040g04432	1,007	0,005	4Fe-4S ferredoxin, iron-sulfur binding isoform 1	4Fe-4S ferredoxin, iron-sulfur binding isoform 1 [Theobroma cacao]	Over Expressed	D24
s00141g10091	1,006	0,035	pleiotropic drug resistance protein 2	Pleiotropic drug resistance protein 2 [Morus notabilis]	Over Expressed	D24
s00006g01040	1,004	0,000	shaggy-related protein kinase epsilon	Shaggy-related protein kinase epsilon [Morus notabilis]	Over Expressed	D24
s00733g23191	1,004	0,001	similar to L484_016704	### Not characterized ###:hypothetical protein L484_016704 [Morus notabilis]	Over Expressed	D24
s03164g31182	-7,573	0,000	LRR receptor-like serine/threonine-protein kinase	putative LRR receptor-like serine/threonine-protein kinase [Morus notabilis] gi|587885757|gb|EXB74614.1|	Under Expressed	D24
s00896g25043	-7,544	0,004	calcium-binding protein CML37	Calcium-binding protein CML37 [Morus notabilis]	Under Expressed	D24
s00038g04282	-7,437	0,000	21 kDa protein	21 kDa protein [Morus notabilis] gi|587951022|gb|EXC36884.1| 21 kDa protein [Morus notabilis]	Under Expressed	D24
s00172g11376	-7,128	0,046	N-acetyltransferase p20	putative N-acetyltransferase p20 [Morus notabilis]	Under Expressed	D24
s00069g06538	-7,121	0,004	-	wound-induced protein precursor [Glycine max]	Under Expressed	D24
s01095g26791	-6,670	0,008	GDSL esterase/lipase	GDSL esterase/lipase [Morus notabilis] gi|587868944|gb|EXB58276.1|	Under Expressed	D24
s00006g01076	-6,572	0,009	-	***** No hits found ******	Under Expressed	D24
s00051g05321	-6,530	0,039	-	G-type lectin S-receptor-like serine/threonine-protein kinase [Morus notabilis] gi|587837766|gb|EXB28514.1|	Under Expressed	D24
s00031g03733	-6,347	0,017	-	***** No hits found ******	Under Expressed	D24
s00035g04111	-6,285	0,031	-	3-isopropylmalate dehydratase large subunit [Gossypium arboreum]	Under Expressed	D24
s00173g11396	-6,285	0,002	endochitinase	Endochitinase [Morus notabilis] gi|587865661|gb|EXB55191.1|	Under Expressed	D24
s00527g20166	-6,191	0,039	homeobox-leucine zipper protein ATHB-21	Homeobox-leucine zipper protein ATHB-21 [Morus notabilis]	Under Expressed	D24
s00008g01366	-6,185	0,026	ethylene-responsive transcription factor	Ethylene-responsive transcription factor [Morus notabilis]	Under Expressed	D24
s00344g16347	-6,170	0,008	-	***** No hits found ******	Under Expressed	D24
s00634g21885	-6,140	0,035	-	PREDICTED: proline-rich protein 4-like [Vigna radiata var. radiata]	Under Expressed	D24

s00288g14928	-6,011	0,039	flowering promoting factor 1	Flowering promoting factor 1 [Theobroma cacao]	Under Expressed	D24
s01500g29040	-5,999	0,000	-	PREDICTED: calcium-binding protein CML39-like [Gossypium raimondii]	Under Expressed	D24
s00096g08052	-5,963	0,019	-	putative receptor-like protein kinase [Morus notabilis]	Under Expressed	D24
s00116g09037	-5,963	0,018	-	PREDICTED: protein IDA [Prunus mume]	Under Expressed	D24
s01300g28071	-5,952	0,035	-	Cysteine-rich RLK (RECEPTOR-like protein kinase) 8 [Theobroma cacao] gi|508724766|gb|EOY16663.1|	Under Expressed	D24
s00014g02101	-5,624	0,038	anthocyanin regulatory C1 protein	Anthocyanin regulatory C1 protein [Morus notabilis]	Under Expressed	D24
s01834g30071	-5,552	0,001	LRR receptor-like serine/threonine-protein kinase	putative LRR receptor-like serine/threonine-protein kinase [Morus notabilis] gi|587885756|gb|EXB74613.1|	Under Expressed	D24
s00164g11047	-5,496	0,025	chalcone synthase	Chalcone synthase [Morus notabilis] gi|587888218|gb|EXB76929.1|	Under Expressed	D24
s00653g22150	-5,477	0,023	BON1-associated protein 2	BON1-associated protein 2 [Morus notabilis]	Under Expressed	D24
s01494g29013	-5,191	0,015	-	PREDICTED: transcription factor PAR1-like [Gossypium raimondii]	Under Expressed	D24
s00470g19104	-5,149	0,000	-	Calcium-binding EF-hand family protein, putative [Theobroma cacao]	Under Expressed	D24
s00074g06836	-5,107	0,016	-	PREDICTED: B3 domain-containing transcription factor NGA4-like [Populus euphratica]	Under Expressed	D24
s00004g00728	-5,099	0,015	-	***** No hits found ******	Under Expressed	D24
s03966g31442	-5,092	0,009	-	***** No hits found ******	Under Expressed	D24
s15689g34250	-5,090	0,009	-	gag/pol protein [Bryonia dioica]	Under Expressed	D24
s00032g03857	-5,089	0,007	RING-H2 finger protein ATL3	RING-H2 finger protein ATL3 [Morus notabilis]	Under Expressed	D24
s01042g26401	-5,022	0,018	similar to L484_011611	### Not characterized ###:hypothetical protein L484_011611 [Morus notabilis]	Under Expressed	D24
s00303g15320	-4,997	0,026	sex determination protein tasselseed-2	Sex determination protein tasselseed-2 [Morus notabilis]	Under Expressed	D24
s00244g13737	-4,959	0,029	CI small heat shock protein 2	CI small heat shock protein 2 [Prunus salicina]	Under Expressed	D24
s01804g30004	-4,876	0,012	ethylene-responsive transcription factor 1B	Ethylene-responsive transcription factor 1B [Morus notabilis]	Under Expressed	D24
s00173g11403	-4,828	0,006	-	### Not characterized ###:Uncharacterized protein TCM_019918 [Theobroma cacao]	Under Expressed	D24

s00041g04548	-4,808	0,000	-	Coatomer subunit beta'-3 [Morus notabilis]	Under Expressed	D24
s00035g04069	-4,807	0,026	late embryogenesis abundant hydroxyproline-rich glycoprotein family	Late embryogenesis abundant hydroxyproline-rich glycoprotein family, putative [Theobroma cacao]	Under Expressed	D24
s00031g03735	-4,781	0,024	-	***** No hits found ******	Under Expressed	D24
s00232g13385	-4,693	0,023	-	### Not characterized ###:hypothetical protein L484_005249 [Morus notabilis]	Under Expressed	D24
s00458g18863	-4,683	0,037	(RS)-norcoclaurine 6-O-methyltransferase	(RS)-norcoclaurine 6-O-methyltransferase [Morus notabilis]	Under Expressed	D24
s01060g26543	-4,665	0,033	-	calciumion binding protein, putative [Ricinus communis]	Under Expressed	D24
s00123g09309	-4,635	0,037	tabersonine 16-O-methyltransferase	Tabersonine 16-O-methyltransferase [Morus notabilis]	Under Expressed	D24
s00147g10417	-4,570	0,009	-	### Not characterized ###:hypothetical protein L484_024119 [Morus notabilis]	Under Expressed	D24
s01171g27344	-4,560	0,013	-	### Not characterized ###:hypothetical protein L484_018218 [Morus notabilis]	Under Expressed	D24
s00299g15226	-4,546	0,016	WRKY transcription factor 18	WRKY transcription factor 18 [Morus notabilis]	Under Expressed	D24
s00303g15319	-4,532	0,000	wound-induced protein 1	PREDICTED: wound-induced protein 1 [Fragaria vesca subsp. vesca]	Under Expressed	D24
s00076g06935	-4,526	0,000	pathogenesis-related protein transcriptional activator PTI6	Pathogenesis-related protein transcriptional activator PTI6 [Morus notabilis] gi|587866561|gb|EXB56018.1|	Under Expressed	D24
s00148g10443	-4,497	0,002	stachyose synthase-like	PREDICTED: stachyose synthase-like [Prunus mume]	Under Expressed	D24
s00312g15580	-4,485	0,047	-	PREDICTED: ethylene-responsive transcription factor ERF096-like [Pyrus x bretschneideri]	Under Expressed	D24
s00069g06537	-4,485	0,032	wound-induced protein WIN2	Wound-induced protein WIN2 [Morus notabilis]	Under Expressed	D24
s00006g01088	-4,455	0,004	cytochrome P450 86B1-like	PREDICTED: cytochrome P450 86B1-like [Populus euphratica]	Under Expressed	D24
s00004g00684	-4,394	0,000	galactose oxidase	galactose oxidase [Ziziphus jujuba]	Under Expressed	D24
s00008g01340	-4,391	0,040	-	putative WRKY transcription factor 51 [Morus notabilis]	Under Expressed	D24
s00644g22030	-4,338	0,023	-	Glycolipid transfer domain-containing protein 1 [Gossypium arboreum]	Under Expressed	D24

s00458g18864	-4,326	0,000	-	(RS)-norcoclaurine 6-O-methyltransferase [Morus notabilis]	Under Expressed	D24
s00004g00779	-4,266	0,000	dnaJ homolog subfamily C member 1	DnaJ homolog subfamily C member 1 [Morus notabilis]	Under Expressed	D24
s00172g11377	-4,131	0,030	N-acetyltransferase p20	putative N-acetyltransferase p20 [Morus notabilis]	Under Expressed	D24
s00185g11839	-4,130	0,028	metacaspase-1-like	PREDICTED: metacaspase-1-like [Jatropha curcas]	Under Expressed	D24
s00192g12067	-4,121	0,000	aspartic proteinase nepenthesin-1	Aspartic proteinase nepenthesin-1 [Morus notabilis]	Under Expressed	D24
s00068g06436	-4,070	0,045	E3 ubiquitin-protein ligase RGLG2	E3 ubiquitin-protein ligase RGLG2 [Morus notabilis]	Under Expressed	D24
s00001g00155	-4,066	0,049	-	### Not characterized ###:hypothetical protein L484_012747 [Morus notabilis]	Under Expressed	D24
s00181g11661	-4,048	0,030	serine acetyltransferase 3, mitochondrial	Serine acetyltransferase 3, mitochondrial, putative [Theobroma cacao] gi|508781685|gb|EOY28941.1|	Under Expressed	D24
s00039g04387	-4,030	0,000	similar to L484_000104	### Not characterized ###:hypothetical protein L484_000104 [Morus notabilis]	Under Expressed	D24
s00040g04463	-3,999	0,000	-	GAST-like gene product family protein [Populus trichocarpa]	Under Expressed	D24
s00256g14080	-3,970	0,015	heat stress transcription factor A-2c	Heat stress transcription factor A-2c, putative [Theobroma cacao]	Under Expressed	D24
s00322g15868	-3,966	0,026	thaumatin-like protein 1	PREDICTED: thaumatin-like protein 1 [Cicer arietinum]	Under Expressed	D24
s01243g27793	-3,960	0,043	wall-associated receptor kinase-like 9	Wall-associated receptor kinase-like 9 [Morus notabilis]	Under Expressed	D24
s00055g05566	-3,940	0,006	-	PREDICTED: LOW QUALITY PROTEIN: protein PLANT CADMIUM RESISTANCE 8 [Vigna radiata var. radiata]	Under Expressed	D24
s02226g30702	-3,932	0,048	transcription factor bHLH100-like	PREDICTED: transcription factor bHLH100-like [Fragaria vesca subsp. vesca]	Under Expressed	D24
s00033g03927	-3,932	0,002	phosphatidylcholine:diacylglycerol cholinephosphotransferase 1	PREDICTED: phosphatidylcholine:diacylglycerol cholinephosphotransferase 1 [Glycine max]	Under Expressed	D24
s01500g29037	-3,931	0,007	-	putative calcium-binding protein CML10 [Morus notabilis]	Under Expressed	D24
s00614g21595	-3,919	0,044	auxin-induced protein 5NG4	Auxin-induced protein 5NG4 [Morus notabilis]	Under Expressed	D24

s18275g34741	-3,891	0,038	pentatricopeptide repeat-containing protein	Pentatricopeptide repeat-containing protein [Morus notabilis]	Under Expressed	D24
s00218g12961	-3,873	0,012	-	PREDICTED: sigma factor binding protein 1, chloroplastic-like [Populus euphratica]	Under Expressed	D24
s00581g21074	-3,845	0,017	-	***** No hits found ******	Under Expressed	D24
s00101g08292	-3,841	0,004	ethylene-responsive transcription factor 2	Ethylene-responsive transcription factor 2 [Morus notabilis]	Under Expressed	D24
s00977g25835	-3,837	0,043	-	putative LRR receptor-like serine/threonine-protein kinase [Morus notabilis] gi|587885757|gb|EXB74614.1|	Under Expressed	D24
s00608g21507	-3,826	0,013	lactosylceramide 4-alpha-galactosyltransferase-like	PREDICTED: lactosylceramide 4-alpha-galactosyltransferase-like [Fragaria vesca subsp. vesca]	Under Expressed	D24
s00022g02980	-3,773	0,022	-	### Not characterized ###:hypothetical protein L484_024544 [Morus notabilis]	Under Expressed	D24
s00863g24669	-3,771	0,028	-	### Not characterized ###:hypothetical protein L484_000041 [Morus notabilis]	Under Expressed	D24
s00356g16649	-3,765	0,005	-	G-type lectin S-receptor-like serine/threonine-protein kinase [Morus notabilis] gi|587865878|gb|EXB55394.1|	Under Expressed	D24
s00034g03977	-3,746	0,016	-	***** No hits found ******	Under Expressed	D24
s00017g02383	-3,743	0,035	scarecrow-like protein 30	PREDICTED: scarecrow-like protein 30 [Gossypium raimondii]	Under Expressed	D24
s00028g03434	-3,730	0,004	proteasome subunit beta type-3-A	Proteasome subunit beta type-3-A [Morus notabilis]	Under Expressed	D24
s00112g08820	-3,715	0,015	RING-H2 finger protein ATL2	RING-H2 finger protein ATL2 [Morus notabilis]	Under Expressed	D24
s00298g15169	-3,692	0,048	anthocyanin regulatory C1 protein	Anthocyanin regulatory C1 protein [Morus notabilis]	Under Expressed	D24
s00116g09020	-3,691	0,000	-	leucoanthocyanidin reductase [Humulus lupulus]	Under Expressed	D24
s06315g32096	-3,681	0,001	(RS)-norcoclaurine 6-O-methyltransferase	(RS)-norcoclaurine 6-O-methyltransferase [Morus notabilis]	Under Expressed	D24
s00062g06063	-3,666	0,031	serine/threonine-protein kinase	Serine/threonine-protein kinase [Morus notabilis]	Under Expressed	D24
s00574g20968	-3,655	0,021	-	PREDICTED: glycine-rich cell wall structural protein 1-like [Populus euphratica]	Under Expressed	D24
s00129g09589	-3,621	0,022	similar to L484_026553	### Not characterized ###:hypothetical protein L484_026553 [Morus notabilis]	Under Expressed	D24
s00138g09998	-3,609	0,023	formin-like protein 8	Formin-like protein 8 [Morus notabilis] gi|587894133|gb|EXB82665.1|	Under Expressed	D24

s00037g04216	-3,606	0,029	peroxidase 51-like	PREDICTED: peroxidase 51-like [Gossypium raimondii]	Under Expressed	D24
s00823g24268	-3,605	0,022	receptor-like protein kinase	putative receptor-like protein kinase [Morus notabilis]	Under Expressed	D24
s00356g16650	-3,598	0,013	G-type lectin S-receptor-like serine/threonine-protein kinase	G-type lectin S-receptor-like serine/threonine-protein kinase [Morus notabilis] gi|587865878|gb|EXB55394.1|	Under Expressed	D24
s01296g28050	-3,591	0,005	auxin-induced protein 5NG4	Auxin-induced protein 5NG4 [Morus notabilis]	Under Expressed	D24
s00297g15152	-3,559	0,042	-	putative S-adenosylmethionine-dependent methyltransferase [Morus notabilis] gi|587935268|gb|EXC22151.1|	Under Expressed	D24
s00116g09014	-3,553	0,000	AP2/ERF and B3 domain-containing transcription factor RAV1	AP2/ERF and B3 domain-containing transcription factor RAV1 [Morus notabilis] gi|587864332|gb|EXB53997.1|	Under Expressed	D24
s00111g08794	-3,550	0,033	RING/U-box superfamily protein, putative isoform 1	RING/U-box superfamily protein, putative isoform 1 [Theobroma cacao] gi|590643833|ref|XP_007030912.1|	Under Expressed	D24
s03907g31418	-3,546	0,005	-	***** No hits found ******	Under Expressed	D24
s08835g32784	-3,526	0,047	wall-associated receptor kinase 2-like	PREDICTED: wall-associated receptor kinase 2-like [Malus domestica]	Under Expressed	D24
s01087g26736	-3,525	0,019	-	### Not characterized ###:hypothetical protein L484_021517 [Morus notabilis]	Under Expressed	D24
s00634g21887	-3,519	0,001	BAHD acyltransferase DCR	BAHD acyltransferase DCR [Morus notabilis]	Under Expressed	D24
s00862g24651	-3,479	0,001	-	### Not characterized ###:hypothetical protein L484_023020 [Morus notabilis]	Under Expressed	D24
s00082g07325	-3,477	0,000	RING finger and CHY zinc finger domain-containing protein 1	RING fingerand CHY zinc finger domain-containing protein 1 [Morus notabilis] gi|587887097|gb|EXB75898.1|	Under Expressed	D24
s01514g29100	-3,463	0,033	-	PREDICTED: glutelin type-A 1-like [Nelumbo nucifera]	Under Expressed	D24
s00069g06516	-3,461	0,003	beta-galactosidase	beta-galactosidase [Prunus salicina]	Under Expressed	D24
s00137g09948	-3,450	0,034	serine/threonine-protein kinase-like protein	Putative serine/threonine-protein kinase-like protein [Morus notabilis] gi|587900038|gb|EXB88391.1|	Under Expressed	D24
s00594g21274	-3,433	0,004	10-deacetylbaccatin III 10-O-acetyltransferase	10-deacetylbaccatin III 10-O-acetyltransferase [Morus notabilis]	Under Expressed	D24
s00123g09330	-3,424	0,026	zinc finger protein ZAT10	PREDICTED: zinc finger protein ZAT10 [Populus euphratica]	Under Expressed	D24
s07127g32320	-3,396	0,033	heat shock cognate 70 kDa protein 2-like	PREDICTED: LOW QUALITY PROTEIN: heat shock cognate 70 kDa protein 2-like [Jatropha curcas]	Under Expressed	D24
s00581g21073	-3,387	0,015	-	### Not characterized ###:hypothetical protein L484_013065 [Morus notabilis]	Under Expressed	D24

s02234g30709	-3,371	0,024	ankyrin repeat family protein	Ankyrin repeat family protein [Theobroma cacao]	Under Expressed	D24
s00289g14954	-3,368	0,039	pathogenesis-related protein transcriptional activator PTI5	Pathogenesis-related protein transcriptional activator PTI5 [Morus notabilis] gi|587945628|gb|EXC32022.1|	Under Expressed	D24
s00001g00002	-3,368	0,023	-	### Not characterized ###:hypothetical protein L484_013383 [Morus notabilis]	Under Expressed	D24
s00138g09990	-3,363	0,031	metalloendoproteinase 1	Metalloendoproteinase 1 [Morus notabilis]	Under Expressed	D24
s01171g27343	-3,344	0,030	nudix hydrolase 2	Nudix hydrolase 2 [Morus notabilis] gi|587845513|gb|EXB36060.1|	Under Expressed	D24
s00034g03978	-3,335	0,026	WRKY transcription factor 40	putative WRKY transcription factor 40 [Morus notabilis]	Under Expressed	D24
s00030g03656	-3,317	0,024	calcium-transporting ATPase 2, plasma membrane-type	Calcium-transporting ATPase 2, plasma membrane-type [Morus notabilis]	Under Expressed	D24
s00531g20245	-3,298	0,001	thaumatin-like protein isoform 2	thaumatin-like protein isoform 2 [Ficus pumila var. awkeotsang]	Under Expressed	D24
s00077g07029	-3,296	0,034	WRKY transcription factor 70	putative WRKY transcription factor 70 [Morus notabilis]	Under Expressed	D24
s00101g08300	-3,286	0,025	ethylene-responsive transcription factor 6	Ethylene-responsive transcription factor 6 [Morus notabilis]	Under Expressed	D24
s00078g07106	-3,285	0,015	NAC domain-containing protein 2	NAC domain-containing protein 2 [Morus notabilis]	Under Expressed	D24
s00628g21792	-3,276	0,024	zingipain-2-like	PREDICTED: zingipain-2-like [Gossypium raimondii]	Under Expressed	D24
s00040g04475	-3,274	0,000	chaperone protein dnaJ 11	Chaperone protein dnaJ 11 [Morus notabilis]	Under Expressed	D24
s01841g30084	-3,273	0,039	G-type lectin S-receptor-like serine/threonine-protein kinase At2g19130	PREDICTED: G-type lectin S-receptor-like serine/threonine-protein kinase At2g19130 [Pyrus x bretschneideri]	Under Expressed	D24
s11294g33374	-3,247	0,035	-	PREDICTED: inositol phosphorylceramide glucuronosyltransferase 1-like [Vigna radiata var. radiata]	Under Expressed	D24
s00234g13451	-3,227	0,026	-	PREDICTED: transcription factor bHLH137-like [Prunus mume]	Under Expressed	D24
s00003g00481	-3,212	0,000	sugar transporter ERD6-like 5	Sugar transporter ERD6-like 5 [Morus notabilis]	Under Expressed	D24
s00376g17144	-3,205	0,043	GDSL esterase/lipase	GDSL esterase/lipase [Morus notabilis] gi|587895731|gb|EXB84225.1|	Under Expressed	D24
s04517g31607	-3,189	0,030	-	***** No hits found ******	Under Expressed	D24
s00014g02127	-3,186	0,027	cytochrome P450 71A1	Cytochrome P450 71A1 [Morus notabilis] gi|587832975|gb|EXB23806.1|	Under Expressed	D24

s00031g03717	-3,186	0,043	-	putative calcium-binding protein CML45 [Morus notabilis]	Under Expressed	D24
s00252g13943	-3,142	0,047	CBS domain-containing protein CBSX1	CBS domain-containing protein CBSX1 [Morus notabilis]	Under Expressed	D24
s00232g13386	-3,142	0,030	-	### Not characterized ###:hypothetical protein L484_005247 [Morus notabilis]	Under Expressed	D24
s13015g33743	-3,135	0,037	LRR receptor-like serine/threonine-protein kinase	putative LRR receptor-like serine/threonine-protein kinase [Morus notabilis] gi|587885757|gb|EXB74614.1|	Under Expressed	D24
s00053g05397	-3,126	0,038	E3 ubiquitin-protein ligase LIN-1	Putative E3 ubiquitin-protein ligase LIN-1 [Morus notabilis]	Under Expressed	D24
s00143g10196	-3,103	0,008	similar to L484_010790	### Not characterized ###:hypothetical protein L484_010790 [Morus notabilis]	Under Expressed	D24
s00567g20827	-3,097	0,048	mitochondrial chaperone bcs1	putative mitochondrial chaperone bcs1 [Morus notabilis]	Under Expressed	D24
s00212g12766	-3,074	0,000	transcription factor bHLH93-like	PREDICTED: transcription factor bHLH93-like [Malus domestica]	Under Expressed	D24
s00042g04632	-3,071	0,002	DNA-damage-repair/toleration protein	DNA-damage-repair/toleration protein [Morus notabilis]	Under Expressed	D24
s00297g15148	-3,067	0,043	S-adenosylmethionine-dependent methyltransferase	putative S-adenosylmethionine-dependent methyltransferase [Morus notabilis] gi|587839619|gb|EXB30273.1|	Under Expressed	D24
s00005g00867	-3,064	0,014	-	PREDICTED: protein SRG1 [Fragaria vesca subsp. vesca]	Under Expressed	D24
s01266g27919	-3,061	0,043	uncharacterized RNA-binding protein	Uncharacterized RNA-binding protein [Morus notabilis]	Under Expressed	D24
s00302g15312	-3,049	0,023	serine/threonine-protein kinase-like protein	Serine/threonine-protein kinase-like protein [Morus notabilis]	Under Expressed	D24
s00932g25401	-3,043	0,044	cytochrome P450 77A3	Cytochrome P450 77A3 [Morus notabilis] gi|587938697|gb|EXC25405.1|	Under Expressed	D24
s00085g07457	-3,041	0,001	-	PREDICTED: CASP-like protein 4D1 [Fragaria vesca subsp. vesca]	Under Expressed	D24
s00005g00953	-3,026	0,002	nucleotide-diphospho-sugar transferase family protein	nucleotide-diphospho-sugar transferase family protein [Medicago truncatula] gi|657371973|gb|KEH17925.1|	Under Expressed	D24
s09261g32886	-3,020	0,038	NAC domain-containing protein 94	Putative NAC domain-containing protein 94 [Morus notabilis]	Under Expressed	D24
s04605g31635	-3,019	0,000	phi-1-like phosphate-induced protein	phi-1-like phosphate-induced protein [Phaseolus vulgaris]	Under Expressed	D24
s00523g20096	-3,016	0,000	cysteine-rich repeat secretory protein 3 isoform X1	PREDICTED: cysteine-rich repeat secretory protein 3 isoform X1 [Fragaria vesca subsp. vesca]	Under Expressed	D24

s12085g33550	-2,996	0,045	-	***** No hits found ******	Under Expressed	D24
s00930g25391	-2,987	0,016	-	***** No hits found ******	Under Expressed	D24
s01500g29038	-2,967	0,017	-	putative calcium-binding protein CML10 [Morus notabilis]	Under Expressed	D24
s01111g26902	-2,918	0,002	BR enhanced expression 1	BR enhanced expression 1 [Theobroma cacao]	Under Expressed	D24
s00220g13014	-2,908	0,000	pectinesterase/pectinesterase inhibitor 35	putative pectinesterase/pectinesterase inhibitor 35 [Morus notabilis] gi|587909537|gb|EXB97446.1|	Under Expressed	D24
s00067g06422	-2,901	0,043	benzyl alcohol O-benzoyltransferase	PREDICTED: benzyl alcohol O-benzoyltransferase [Prunus mume]	Under Expressed	D24
s00544g20469	-2,901	0,047	syntaxin-121 protein	Syntaxin-121 protein [Gossypium arboreum]	Under Expressed	D24
s00210g12684	-2,898	0,002	inactive protein RESTRICTED TEV MOVEMENT 2-like	PREDICTED: inactive protein RESTRICTED TEV MOVEMENT 2-like [Malus domestica]	Under Expressed	D24
s00740g23284	-2,897	0,049	expansin-like protein	Expansin-like protein [Morus notabilis] gi|587940103|gb|EXC26724.1|	Under Expressed	D24
s00519g20037	-2,883	0,049	U-box domain-containing protein 20	U-box domain-containing protein 20 [Morus notabilis]	Under Expressed	D24
s00460g18925	-2,863	0,006	annexin D3	Annexin D3 [Morus notabilis] gi|587873455|gb|EXB62640.1|	Under Expressed	D24
s00361g16784	-2,863	0,038	disease resistance RPP13-like protein 4	PREDICTED: disease resistance RPP13-like protein 4 [Prunus mume]	Under Expressed	D24
s00133g09790	-2,859	0,039	-	protein binding protein, putative [Ricinus communis]	Under Expressed	D24
s00236g13502	-2,857	0,011	similar to L484_025643	### Not characterized ###:hypothetical protein L484_025643 [Morus notabilis]	Under Expressed	D24
s00101g08299	-2,848	0,039	-	***** No hits found ******	Under Expressed	D24
s04524g31609	-2,844	0,033	inactive purple acid phosphatase 27	putative inactive purple acid phosphatase 27 [Morus notabilis]	Under Expressed	D24
s00041g04547	-2,834	0,002	coatomer subunit beta'-3	Coatomer subunit beta'-3 [Morus notabilis]	Under Expressed	D24
s01296g28049	-2,829	0,031	auxin-induced protein 5NG4	Auxin-induced protein 5NG4 [Morus notabilis]	Under Expressed	D24
s00443g18558	-2,818	0,006	EG45-like domain containing protein	PREDICTED: EG45-like domain containing protein [Fragaria vesca subsp. vesca]	Under Expressed	D24
s01087g26735	-2,795	0,048	-	### Not characterized ###:hypothetical protein L484_021517 [Morus notabilis]	Under Expressed	D24
s00007g01222	-2,790	0,014	mitochondrial uncoupling protein 5	PREDICTED: mitochondrial uncoupling protein 5 [Cucumis sativus]	Under Expressed	D24
s00337g16177	-2,790	0,002	nematode resistance protein-like HSPRO2	PREDICTED: nematode resistance protein-like HSPRO2 [Prunus mume]	Under Expressed	D24

s01332g28263	-2,768	0,029	uncharacterized LOC105121148	uncharacterized LOC105121148 [Populus euphratica]	Under Expressed	D24
s00132g09705	-2,767	0,030	WRKY transcription factor 46	putative WRKY transcription factor 46 [Morus notabilis]	Under Expressed	D24
s00640g21976	-2,765	0,000	plasmamembrane intrinsic protein 1	plasmamembrane intrinsic protein 1;3 [Salix purpurea]	Under Expressed	D24
s00581g21075	-2,762	0,016	-	### Not characterized ###:hypothetical protein L484_013065 [Morus notabilis]	Under Expressed	D24
s00492g19511	-2,757	0,034	lysine histidine transporter 1-like	PREDICTED: lysine histidine transporter 1-like [Tarenaya hassleriana]	Under Expressed	D24
s00517g19996	-2,749	0,022	-	Chloride channel protein CLC-f [Morus notabilis]	Under Expressed	D24
s01221g27665	-2,742	0,000	alpha,alpha-trehalose-phosphate synthase	putative alpha,alpha-trehalose-phosphate synthase [UDP-forming] 11 [Morus notabilis] gi|587935354|gb|EXC22233.1|	Under Expressed	D24
s00146g10358	-2,725	0,024	NAC domain-containing protein 94	Putative NAC domain-containing protein 94 [Morus notabilis]	Under Expressed	D24
s00028g03479	-2,663	0,044	glutamate receptor 2.7	Glutamate receptor 2.7 [Morus notabilis]	Under Expressed	D24
s00566g20814	-2,659	0,019	-	### Not characterized ###:hypothetical protein L484_005841 [Morus notabilis]	Under Expressed	D24
s00185g11856	-2,655	0,007	cyclin-U4-1-like	PREDICTED: cyclin-U4-1-like [Nelumbo nucifera]	Under Expressed	D24
s00351g16530	-2,652	0,008	transcription factor	Transcription factor [Morus notabilis] gi|587874541|gb|EXB63679.1|	Under Expressed	D24
s00153g10630	-2,650	0,026	HTH-type transcriptional regulator protein ptxE	HTH-type transcriptional regulator protein ptxE [Theobroma cacao]	Under Expressed	D24
s00380g17222	-2,644	0,019	F-box protein At2g27310-like	PREDICTED: F-box protein At2g27310-like [Fragaria vesca subsp. vesca]	Under Expressed	D24
s00021g02839	-2,619	0,020	transcription factor	Transcription factor [Morus notabilis] gi|587877853|gb|EXB66876.1|	Under Expressed	D24
s27635g36101	-2,616	0,037	-	Heavy metal transport/detoxification superfamily protein, putative [Theobroma cacao] gi|508701700|gb|EOX93596.1|	Under Expressed	D24
s00470g19108	-2,614	0,041	-	Polcalcin Jun o 2 [Morus notabilis] gi|587932722|gb|EXC19749.1|	Under Expressed	D24
s00460g18926	-2,613	0,033	annexin D4	Annexin D4 [Morus notabilis] gi|587873456|gb|EXB62641.1|	Under Expressed	D24
s00186g11900	-2,612	0,001	oligopeptide transporter 6	Oligopeptide transporter 6 [Morus notabilis]	Under Expressed	D24

s10131g33102	-2,612	0,000	COBRA-like protein 2 precursor	COBRA-like protein 2 precursor [Medicago truncatula]	Under Expressed	D24
s00760g23514	-2,605	0,023	-	***** No hits found ******	Under Expressed	D24
s00154g10665	-2,594	0,030	-	***** No hits found ******	Under Expressed	D24
s00090g07772	-2,593	0,016	vinorine synthase	Vinorine synthase [Morus notabilis] gi|587893898|gb|EXB82430.1|	Under Expressed	D24
s00270g14464	-2,583	0,000	F-box/kelch-repeat protein	F-box/kelch-repeat protein [Morus notabilis]	Under Expressed	D24
s00023g03039	-2,579	0,046	WRKY transcription factor 41	putative WRKY transcription factor 41 [Morus notabilis]	Under Expressed	D24
s00266g14372	-2,578	0,006	ceramide-1-phosphate transfer protein	PREDICTED: ceramide-1-phosphate transfer protein [Cucumis sativus]	Under Expressed	D24
s00653g22160	-2,568	0,006	-	***** No hits found ******	Under Expressed	D24
s00012g01791	-2,566	0,042	receptor-like protein 12	PREDICTED: receptor-like protein 12 [Prunus mume]	Under Expressed	D24
s00131g09696	-2,554	0,037	-	Importin subunit alpha [Morus notabilis]	Under Expressed	D24
s00079g07120	-2,553	0,009	6,7-dimethyl-8-ribityllumazine synthase	6,7-dimethyl-8-ribityllumazine synthase [Theobroma cacao]	Under Expressed	D24
s00001g00176	-2,546	0,020	-	PREDICTED: protein EXORDIUM-like [Glycine max]	Under Expressed	D24
s00001g00238	-2,544	0,001	glucan 1,3-beta-glucosidase A	putative glucan 1,3-beta-glucosidase A [Morus notabilis]	Under Expressed	D24
s00469g19086	-2,531	0,015	-	***** No hits found ******	Under Expressed	D24
s01347g28340	-2,495	0,004	G-type lectin S-receptor-like serine/threonine-protein kinase SD2-5	G-type lectin S-receptor-like serine/threonine-protein kinase SD2-5 [Morus notabilis] gi|587904174|gb|EXB92375.1|	Under Expressed	D24
s00025g03198	-2,494	0,031	-	***** No hits found ******	Under Expressed	D24
s00464g18997	-2,475	0,001	galactose oxidase	PREDICTED: galactose oxidase [Jatropha curcas]	Under Expressed	D24
s00426g18219	-2,464	0,000	zinc finger protein ZAT10	Zinc finger protein ZAT10 [Morus notabilis]	Under Expressed	D24
s00163g11009	-2,453	0,041	galactinol synthase 2-like	PREDICTED: galactinol synthase 2-like [Fragaria vesca subsp. vesca]	Under Expressed	D24
s00891g25008	-2,446	0,022	reticuline oxidase-like protein	Reticuline oxidase-like protein [Morus notabilis]	Under Expressed	D24
s00253g13969	-2,441	0,004	formin-like protein 5	Formin-like protein 5 [Morus notabilis] gi|587861076|gb|EXB50940.1|	Under Expressed	D24
s00800g23992	-2,440	0,004	glutamine amidotransferase	Putative Glutamine amidotransferase [Morus notabilis]	Under Expressed	D24
s00191g12061	-2,433	0,002	glutaredoxin family protein	Glutaredoxin family protein, putative [Theobroma cacao]	Under Expressed	D24

s00133g09773	-2,426	0,022	-	PREDICTED: protein BPS1, chloroplastic-like [Prunus mume]	Under Expressed	D24
s00012g01869	-2,421	0,009	chitotriosidase-1-like	PREDICTED: chitotriosidase-1-like [Prunus mume]	Under Expressed	D24
s00567g20842	-2,418	0,037	ocs element-binding factor 1	Ocs element-binding factor 1 [Morus notabilis]	Under Expressed	D24
s00203g12462	-2,416	0,005	ABC transporter G family member 6	ABC transporter G family member 6 [Morus notabilis]	Under Expressed	D24
s00483g19354	-2,416	0,001	-	***** No hits found ******	Under Expressed	D24
s00451g18730	-2,415	0,030	GEM-like protein 8	PREDICTED: putative GEM-like protein 8 [Vitis vinifera]	Under Expressed	D24
s00278g14658	-2,400	0,000	-	### Not characterized ###:PREDICTED: uncharacterized protein LOC104234424 [Nicotiana sylvestris]	Under Expressed	D24
s01280g27982	-2,394	0,019	wall-associated receptor kinase-like 10	Wall-associated receptor kinase-like 10 [Morus notabilis]	Under Expressed	D24
s00256g14066	-2,376	0,014	RING-H2 finger protein ATL11	RING-H2 finger protein ATL11 [Morus notabilis]	Under Expressed	D24
s00556g20661	-2,372	0,019	uncharacterized protein TCM_015048	### Not characterized ###:Uncharacterized protein TCM_015048 [Theobroma cacao]	Under Expressed	D24
s00113g08891	-2,348	0,001	inactive purple acid phosphatase 1 -like protein	putative inactive purple acid phosphatase 1 -like protein [Gossypium arboreum]	Under Expressed	D24
s00200g12349	-2,342	0,023	-	stress induced protein [Vitis vinifera] gi|225580061|gb|ACN94268.1| stress induced protein [Vitis vinifera]	Under Expressed	D24
s00092g07859	-2,341	0,032	-	PREDICTED: E3 ubiquitin-protein ligase RING1-like [Nelumbo nucifera]	Under Expressed	D24
s00004g00731	-2,339	0,026	beta-galactosidase 3	Beta-galactosidase 3 [Morus notabilis] gi|587938279|gb|EXC25028.1|	Under Expressed	D24
s00604g21446	-2,334	0,000	similar to L484_003785	### Not characterized ###:hypothetical protein L484_003785 [Morus notabilis]	Under Expressed	D24
s00059g05860	-2,324	0,000	-	Homeobox-leucine zipper protein HOX9, putative [Theobroma cacao]	Under Expressed	D24
s00183g11766	-2,310	0,003	senescence associated gene 20	Senescence associated gene 20, putative [Theobroma cacao]	Under Expressed	D24
s01003g26053	-2,281	0,015	aromatic-L-amino-acid decarboxylase	Aromatic-L-amino-acid decarboxylase [Morus notabilis]	Under Expressed	D24
s00412g17914	-2,281	0,035	oligopeptide transporter 1	Oligopeptide transporter 1 [Morus notabilis]	Under Expressed	D24
s00004g00730	-2,281	0,010	beta-galactosidase 3	Beta-galactosidase 3 [Morus notabilis] gi|587938279|gb|EXC25028.1|	Under Expressed	D24

s01823g30047	-2,222	0,001	PAE	PAE [Litchi chinensis]	Under Expressed	D24
s00393g17505	-2,221	0,042	alpha carbonic anhydrase 4-like	PREDICTED: alpha carbonic anhydrase 4-like [Eucalyptus grandis]	Under Expressed	D24
s00045g04877	-2,209	0,012	guanine nucleotide-binding protein alpha-1 subunit	Guanine nucleotide-binding protein alpha-1 subunit [Morus notabilis] gi|587874507|gb|EXB63645.1|	Under Expressed	D24
s00317g15702	-2,208	0,025	ethylene-responsive transcription factor 4	Ethylene-responsive transcription factor 4 [Morus notabilis]	Under Expressed	D24
s00001g00171	-2,196	0,012	phospho-2-dehydro-3-deoxyheptonate aldolase 2	Phospho-2-dehydro-3-deoxyheptonate aldolase 2 [Morus notabilis]	Under Expressed	D24
s00010g01581	-2,192	0,000	ACT domain-containing protein ACR1	PREDICTED: ACT domain-containing protein ACR1 [Fragaria vesca subsp. vesca] gi|764516880|ref|XP_011466252.1|	Under Expressed	D24
s00117g09048	-2,181	0,012	LRR receptor-like serine/threonine-protein kinase At4g26540	PREDICTED: probable LRR receptor-like serine/threonine-protein kinase At4g26540 [Prunus mume]	Under Expressed	D24
s00012g01864	-2,180	0,021	similar to L484_014458	### Not characterized ###:hypothetical protein L484_014458 [Morus notabilis]	Under Expressed	D24
s00343g16343	-2,178	0,001	auxin-induced protein X15-like	PREDICTED: auxin-induced protein X15-like [Pyrus x bretschneideri]	Under Expressed	D24
s00044g04826	-2,174	0,006	S-adenosylmethionine decarboxylase proenzyme 3	S-adenosylmethionine decarboxylase proenzyme 3 [Morus notabilis]	Under Expressed	D24
s00203g12461	-2,165	0,045	caffeoylshikimate esterase-like	PREDICTED: caffeoylshikimate esterase-like [Pyrus x bretschneideri]	Under Expressed	D24
s00062g06083	-2,155	0,005	receptor-like protein kinase HAIKU2	Receptor-like protein kinase HAIKU2 [Morus notabilis]	Under Expressed	D24
s00010g01619	-2,151	0,016	xyloglucan endotransglucosylase/hydrolase protein 6	putative xyloglucan endotransglucosylase/hydrolase protein 6 [Morus notabilis] gi|587889580|gb|EXB78247.1|	Under Expressed	D24
s00162g11007	-2,146	0,001	bark storage protein A	Bark storage protein A [Morus notabilis]	Under Expressed	D24
s00419g18069	-2,145	0,001	ethylene-responsive transcription factor 12	Ethylene-responsive transcription factor 12 [Morus notabilis]	Under Expressed	D24
s01010g26115	-2,145	0,000	lysosomal beta glucosidase	Lysosomal beta glucosidase [Morus notabilis]	Under Expressed	D24
s00059g05899	-2,144	0,003	lysM domain-containing GPI-anchored protein 2	LysM domain-containing GPI-anchored protein 2 [Morus notabilis]	Under Expressed	D24
s00098g08137	-2,128	0,001	verprolin-like	PREDICTED: verprolin-like [Gossypium raimondii]	Under Expressed	D24

s00109g08662	-2,123	0,019	non-specific lipid transfer protein GPI-anchored 1-like	PREDICTED: non-specific lipid transfer protein GPI-anchored 1-like [Malus domestica]	Under Expressed	D24
s01041g26392	-2,122	0,046	xyloglucan endotransglucosylase/hydrolase protein 9	Xyloglucan endotransglucosylase/hydrolase protein 9 [Morus notabilis] gi|587936334|gb|EXC23178.1|	Under Expressed	D24
s07452g32410	-2,119	0,000	-	Nodulin MtN21 /EamA-like transporter family protein, putative isoform 1 [Theobroma cacao] gi|508705522|gb|EOX97418.1|	Under Expressed	D24
s00541g20427	-2,117	0,013	inositol oxygenase 1	Inositol oxygenase 1 [Morus notabilis] gi|587885754|gb|EXB74611.1|	Under Expressed	D24
s00069g06515	-2,115	0,006	-	### Not characterized ###:hypothetical protein L484_027272 [Morus notabilis]	Under Expressed	D24
s00048g05051	-2,112	0,004	U-box domain-containing protein 20	U-box domain-containing protein 20 [Morus notabilis]	Under Expressed	D24
s01623g29491	-2,107	0,026	cytochrome P450 71A1-like	PREDICTED: cytochrome P450 71A1-like [Fragaria vesca subsp. vesca]	Under Expressed	D24
s00029g03576	-2,099	0,000	ATP binding protein	ATP binding protein, putative [Ricinus communis]	Under Expressed	D24
s00091g07781	-2,093	0,029	calreticulin-3-like isoform X2	PREDICTED: calreticulin-3-like isoform X2 [Cicer arietinum]	Under Expressed	D24
s00285g14844	-2,087	0,024	-	***** No hits found ******	Under Expressed	D24
s00305g15394	-2,086	0,017	exocyst complex component 7	Exocyst complex component 7 [Morus notabilis]	Under Expressed	D24
s00088g07627	-2,077	0,033	-	### Not characterized ###:hypothetical protein MIMGU_mgv1a014916mg [Erythranthe guttata]	Under Expressed	D24
s01098g26811	-2,062	0,039	ankyrin repeat-containing protein	Ankyrin repeat-containing protein [Morus notabilis]	Under Expressed	D24
s00462g18963	-2,060	0,023	-	***** No hits found ******	Under Expressed	D24
s00075g06904	-2,056	0,047	leucine-rich repeat receptor-like serine/threonine/tyrosine-protein kinase	Leucine-rich repeat receptor-like serine/threonine/tyrosine-protein kinase [Morus notabilis] gi|587906372|gb|EXB94444.1|	Under Expressed	D24
s00832g24347	-2,052	0,001	-	Oxidative stress 3, putative isoform 2 [Theobroma cacao]	Under Expressed	D24
s00007g01181	-2,050	0,003	similar to L484_019803	### Not characterized ###:hypothetical protein L484_019803 [Morus notabilis]	Under Expressed	D24
s00014g02030	-2,040	0,032	beta-galactosidase 1	Beta-galactosidase 1 [Morus notabilis] gi|587923778|gb|EXC11109.1|	Under Expressed	D24
s00587g21145	-2,039	0,033	glutamyl-tRNA reductase 1	Glutamyl-tRNA reductase 1 [Morus notabilis]	Under Expressed	D24
s00019g02669	-2,038	0,007	-	### Not characterized ###:hypothetical protein L484_024428 [Morus notabilis]	Under Expressed	D24

s00163g11019	-2,033	0,000	alkaline/neutral invertase CINV2	PREDICTED: alkaline/neutral invertase CINV2 [Prunus mume]	Under Expressed	D24
s00373g17068	-2,032	0,000	stem-specific protein TSJT1	Stem-specific protein TSJT1, putative [Ricinus communis]	Under Expressed	D24
s19183g34895	-2,022	0,023	-	***** No hits found ******	Under Expressed	D24
s00509g19847	-2,018	0,017	linoleate 9S-lipoxygenase 5	putative linoleate 9S-lipoxygenase 5 [Morus notabilis]	Under Expressed	D24
s00148g10451	-2,004	0,000	glycerol-3-phosphate acyltransferase 3	PREDICTED: probable glycerol-3-phosphate acyltransferase 3 [Populus euphratica]	Under Expressed	D24
s00113g08845	-2,001	0,000	serine/threonine-protein kinase HT1	Serine/threonine-protein kinase HT1 [Morus notabilis]	Under Expressed	D24
s01400g28607	-1,996	0,032	zinc-regulated protein 8-like	PREDICTED: zinc-regulated protein 8-like [Gossypium raimondii]	Under Expressed	D24
s01022g26217	-1,989	0,048	-	***** No hits found ******	Under Expressed	D24
s01842g30086	-1,988	0,028	G-type lectin S-receptor-like serine/threonine-protein kinase	G-type lectin S-receptor-like serine/threonine-protein kinase [Morus notabilis] gi|587922781|gb|EXC10167.1|	Under Expressed	D24
s00539g20370	-1,982	0,003	octicosapeptide/Phox/Bem1p family protein	Octicosapeptide/Phox/Bem1p family protein [Theobroma cacao]	Under Expressed	D24
s00112g08805	-1,981	0,000	bidirectional sugar transporter SWEET17	Bidirectional sugar transporter SWEET17 [Morus notabilis]	Under Expressed	D24
s00611g21551	-1,978	0,008	-	***** No hits found ******	Under Expressed	D24
s00088g07643	-1,978	0,002	pseudo response regulator, putative isoform 4	Pseudo response regulator, putative isoform 4 [Theobroma cacao]	Under Expressed	D24
s00345g16365	-1,977	0,037	leucine-rich repeat receptor-like tyrosine-protein kinase	Leucine-rich repeat receptor-like tyrosine-protein kinase [Morus notabilis] gi|587868024|gb|EXB57397.1|	Under Expressed	D24
s00285g14843	-1,975	0,000	UDP-glucuronate 4-epimerase 6	UDP-glucuronate 4-epimerase 6 [Morus notabilis]	Under Expressed	D24
s00136g09882	-1,971	0,001	homeobox-leucine zipper protein ATHB-7	Homeobox-leucine zipper protein ATHB-7 [Morus notabilis]	Under Expressed	D24
s08589g32731	-1,969	0,024	reticuline oxidase-like protein	Reticuline oxidase-like protein [Morus notabilis]	Under Expressed	D24
s00013g01919	-1,964	0,039	DNA binding protein	DNA binding protein, putative [Ricinus communis]	Under Expressed	D24
s00080g07212	-1,956	0,012	calcium-binding protein CML41	putative calcium-binding protein CML41 [Morus notabilis]	Under Expressed	D24
s00435g18387	-1,949	0,038	reticuline oxidase-like protein	Reticuline oxidase-like protein [Morus notabilis]	Under Expressed	D24
s00069g06534	-1,948	0,004	auxin-responsive protein IAA3	Auxin-responsive protein IAA3 [Morus notabilis]	Under Expressed	D24

s00965g25719	-1,942	0,045	glutathione S-transferase parA	putative glutathione S-transferase parA [Morus notabilis]	Under Expressed	D24
s02447g30886	-1,940	0,000	lupeol synthase	Lupeol synthase [Morus notabilis] gi|587852429|gb|EXB42556.1|	Under Expressed	D24
s00511g19881	-1,939	0,000	GDSL esterase/lipase 1	GDSL esterase/lipase 1 [Morus notabilis]	Under Expressed	D24
s00442g18543	-1,933	0,039	-	PREDICTED: probable protein phosphatase 2C 25 [Pyrus x bretschneideri]	Under Expressed	D24
s00932g25402	-1,933	0,001	cytochrome P450 77A3	Cytochrome P450 77A3 [Morus notabilis] gi|587938697|gb|EXC25405.1|	Under Expressed	D24
s00309g15490	-1,928	0,030	-	CAMP-regulated phosphoprotein 19-related protein isoform 1 [Theobroma cacao] gi|590661689|ref|XP_007035743.1|	Under Expressed	D24
s00667g22355	-1,923	0,049	GATA transcription factor 5-like	PREDICTED: GATA transcription factor 5-like [Prunus mume]	Under Expressed	D24
s01166g27296	-1,920	0,001	mitogen-activated protein kinase kinase 4	Mitogen-activated protein kinase kinase 4 [Morus notabilis]	Under Expressed	D24
s00055g05581	-1,906	0,028	aquaporin TIP2-1-like	PREDICTED: aquaporin TIP2-1-like [Populus euphratica]	Under Expressed	D24
s01267g27927	-1,899	0,032	-	***** No hits found ******	Under Expressed	D24
s00082g07294	-1,891	0,000	neurofilament medium polypeptide	PREDICTED: neurofilament medium polypeptide [Prunus mume]	Under Expressed	D24
s00341g16291	-1,888	0,000	similar to L484_014311	### Not characterized ###:hypothetical protein L484_014311 [Morus notabilis]	Under Expressed	D24
s00232g13384	-1,887	0,008	-	### Not characterized ###:hypothetical protein L484_005250 [Morus notabilis]	Under Expressed	D24
s00681g22516	-1,880	0,025	disease resistance protein RPM1	Disease resistance protein RPM1 [Morus notabilis]	Under Expressed	D24
s00723g23058	-1,878	0,034	histone-lysine N-methyltransferase SETD1B	PREDICTED: histone-lysine N-methyltransferase SETD1B [Vitis vinifera]	Under Expressed	D24
s00006g01064	-1,876	0,018	raffinose synthase family protein	Raffinose synthase family protein [Theobroma cacao]	Under Expressed	D24
s00143g10192	-1,874	0,004	similar to L484_010786	### Not characterized ###:hypothetical protein L484_010786 [Morus notabilis]	Under Expressed	D24
s00149g10512	-1,870	0,002	-	***** No hits found ******	Under Expressed	D24
s00225g13175	-1,866	0,000	similar to L484_003293	### Not characterized ###:hypothetical protein L484_003293 [Morus notabilis]	Under Expressed	D24
s10982g33306	-1,862	0,042	-	PREDICTED: glutaredoxin-C9-like [Populus euphratica]	Under Expressed	D24

s00178g11567	-1,854	0,047	mitotic spindle checkpoint protein MAD2	Mitotic spindle checkpoint protein MAD2 [Populus trichocarpa]	Under Expressed	D24
s00092g07845	-1,850	0,046	perakine reductase-like	PREDICTED: perakine reductase-like [Prunus mume]	Under Expressed	D24
s00102g08340	-1,847	0,024	GDSL esterase/lipase 1	GDSL esterase/lipase 1 [Morus notabilis]	Under Expressed	D24
s00423g18162	-1,841	0,044	peptide transporter	Peptide transporter [Morus notabilis] gi|587913687|gb|EXC01490.1|	Under Expressed	D24
s00361g16755	-1,840	0,032	G-type lectin S-receptor-like serine/threonine-protein kinase	G-type lectin S-receptor-like serine/threonine-protein kinase [Morus notabilis] gi|587924277|gb|EXC11582.1|	Under Expressed	D24
s00154g10691	-1,834	0,029	cytochrome P450 90B1	PREDICTED: cytochrome P450 90B1 [Populus euphratica]	Under Expressed	D24
s00019g02674	-1,824	0,004	L-type lectin-domain containing receptor kinase VIII.1	L-type lectin-domain containing receptor kinase VIII.1 [Morus notabilis] gi|587929073|gb|EXC16248.1|	Under Expressed	D24
s01741g29854	-1,824	0,000	D-3-phosphoglycerate dehydrogenase	D-3-phosphoglycerate dehydrogenase [Morus notabilis]	Under Expressed	D24
s00223g13104	-1,823	0,000	glucan endo-1,3-beta-glucosidase 7	Glucan endo-1,3-beta-glucosidase 7 [Morus notabilis]	Under Expressed	D24
s12983g33737	-1,817	0,025	inter-alpha-trypsin inhibitor heavy chain H3 precursor	Inter-alpha-trypsin inhibitor heavy chain H3 precursor, putative [Ricinus communis] gi|223536573|gb|EEF38218.1|	Under Expressed	D24
s12528g33648	-1,813	0,022	calmodulin-binding protein 25-like	PREDICTED: calmodulin-binding protein 25-like [Glycine max]	Under Expressed	D24
s00075g06895	-1,811	0,019	E3 ubiquitin-protein ligase RGLG2-like isoform X1	PREDICTED: E3 ubiquitin-protein ligase RGLG2-like isoform X1 [Citrus sinensis]	Under Expressed	D24
s00056g05629	-1,799	0,001	peroxidase 47	Peroxidase 47 [Morus notabilis] gi|587874944|gb|EXB64071.1|	Under Expressed	D24
s00496g19600	-1,797	0,011	F-box/kelch-repeat protein	F-box/kelch-repeat protein [Morus notabilis]	Under Expressed	D24
s00425g18184	-1,786	0,043	similar to L484_021665	### Not characterized ###:hypothetical protein L484_021665 [Morus notabilis]	Under Expressed	D24
s00148g10450	-1,782	0,003	glycerol-3-phosphate acyltransferase 3	putative glycerol-3-phosphate acyltransferase 3 [Morus notabilis]	Under Expressed	D24
s00067g06359	-1,780	0,014	glutamine amidotransferase PB2B2.05 isoform X2	PREDICTED: putative glutamine amidotransferase PB2B2.05 isoform X2 [Nicotiana tomentosiformis]	Under Expressed	D24
s00628g21805	-1,771	0,031	similar to L484_006153	### Not characterized ###:hypothetical protein L484_006153 [Morus notabilis]	Under Expressed	D24
s01269g27934	-1,769	0,024	cysteine-rich receptor-like protein kinase 3	Cysteine-rich receptor-like protein kinase 3 [Morus notabilis]	Under Expressed	D24
s00470g19113	-1,767	0,036	-	***** No hits found ******	Under Expressed	D24

s00090g07725	-1,762	0,047	uncharacterized protein LOC103321871	### Not characterized ###:PREDICTED: uncharacterized protein LOC103321871 [Prunus mume]	Under Expressed	D24
s00519g20044	-1,762	0,027	aldose 1-epimerase	Aldose 1-epimerase [Morus notabilis] gi|587906203|gb|EXB94289.1|	Under Expressed	D24
s00481g19303	-1,762	0,002	copperion binding protein	copperion binding protein, putative [Ricinus communis]	Under Expressed	D24
s01358g28404	-1,748	0,034	WRKY transcription factor 69	putative WRKY transcription factor 69 [Morus notabilis]	Under Expressed	D24
s00121g09270	-1,747	0,042	-	PREDICTED: protein PMR5-like [Populus euphratica]	Under Expressed	D24
s00245g13758	-1,746	0,027	riboflavin biosynthesis protein ribBA	Riboflavin biosynthesis protein ribBA [Morus notabilis]	Under Expressed	D24
s00083g07366	-1,739	0,012	UDP-glycosyltransferase 73C3	UDP-glycosyltransferase 73C3 [Morus notabilis]	Under Expressed	D24
s00003g00585	-1,736	0,000	lysine decarboxylase family protein, putative isoform 2	Lysine decarboxylase family protein, putative isoform 2 [Theobroma cacao] gi|508787142|gb|EOY34398.1|	Under Expressed	D24
s00378g17180	-1,732	0,008	LIM and calponin domains-containing protein 1	LIM andcalponin domains-containing protein 1, putative [Theobroma cacao] gi|508720366|gb|EOY12263.1|	Under Expressed	D24
s01537g29184	-1,724	0,015	thaumatin-like protein	PREDICTED: thaumatin-like protein [Fragaria vesca subsp. vesca]	Under Expressed	D24
s00891g25003	-1,719	0,000	reticuline oxidase-like protein	PREDICTED: reticuline oxidase-like protein [Prunus mume]	Under Expressed	D24
s00011g01693	-1,719	0,005	E3 ubiquitin-protein ligase RHA2A	E3 ubiquitin-protein ligase RHA2A [Morus notabilis]	Under Expressed	D24
s00151g10597	-1,717	0,040	-	Dehydrin Rab15 [Morus notabilis] gi|587938113|gb|EXC24886.1|	Under Expressed	D24
s00106g08497	-1,712	0,045	serine/arginine repetitive matrix protein 2	Serine/arginine repetitive matrix protein 2, putative [Theobroma cacao] gi|508773408|gb|EOY20664.1|	Under Expressed	D24
s00046g04922	-1,709	0,029	basic helix-loop-helix DNA-binding family protein	Basic helix-loop-helix DNA-binding family protein [Theobroma cacao]	Under Expressed	D24
s00245g13743	-1,699	0,011	L-type lectin-domain containing receptor kinase S.5	putative L-type lectin-domain containing receptor kinase S.5 [Morus notabilis] gi|587890762|gb|EXB79403.1|	Under Expressed	D24
s00077g07027	-1,697	0,015	cinnamate-4-hydroxylase	Cinnamate-4-hydroxylase [Theobroma cacao]	Under Expressed	D24
s00452g18735	-1,691	0,038	serine/threonine-protein phosphatase 6 regulatory ankyrin repeat subunit A	Serine/threonine-protein phosphatase 6 regulatory ankyrin repeat subunit A [Morus notabilis] gi|587898459|gb|EXB86896.1|	Under Expressed	D24
s00265g14333	-1,687	0,006	glucan endo-1,3-beta-glucosidase 14	Glucan endo-1,3-beta-glucosidase 14 [Morus notabilis]	Under Expressed	D24

s00361g16776	-1,682	0,001	zinc finger protein CONSTANS-LIKE 11	Putative zinc finger protein CONSTANS-LIKE 11 [Morus notabilis]	Under Expressed	D24
s00042g04608	-1,680	0,001	linoleate 13S-lipoxygenase 2-1	Linoleate 13S-lipoxygenase 2-1 [Morus notabilis]	Under Expressed	D24
s00891g25005	-1,678	0,022	reticuline oxidase-like protein	Reticuline oxidase-like protein [Morus notabilis]	Under Expressed	D24
s00391g17463	-1,673	0,046	-	Protein kinase APK1A [Morus notabilis] gi|587943988|gb|EXC30494.1|	Under Expressed	D24
s00751g23411	-1,672	0,000	similar to L484_015990	### Not characterized ###:hypothetical protein L484_015990 [Morus notabilis]	Under Expressed	D24
s00270g14475	-1,666	0,038	-	***** No hits found ******	Under Expressed	D24
s00410g17875	-1,663	0,012	chaperone protein dnaJ 11	Chaperone protein dnaJ 11 [Morus notabilis]	Under Expressed	D24
s00023g03071	-1,659	0,000	-	### Not characterized ###:hypothetical protein L484_001386 [Morus notabilis]	Under Expressed	D24
s00777g23731	-1,657	0,024	leucine-rich repeat receptor-like protein kinase	putative leucine-rich repeat receptor-like protein kinase [Morus notabilis] gi|587877349|gb|EXB66395.1|	Under Expressed	D24
s00030g03627	-1,655	0,000	RING-H2 zinc finger protein RHA1a	RING-H2 zinc finger protein RHA1a [Morus notabilis]	Under Expressed	D24
s00156g10748	-1,650	0,023	-	***** No hits found ******	Under Expressed	D24
s14273g34003	-1,634	0,004	-	### Not characterized ###:hypothetical protein PRUPE_ppa012671mg [Prunus persica]	Under Expressed	D24
s00011g01754	-1,633	0,000	mediator of RNA polymerase II transcription subunit 26	Mediator of RNA polymerase II transcription subunit 26, putative [Theobroma cacao] gi|508700239|gb|EOX92135.1|	Under Expressed	D24
s08346g32666	-1,631	0,025	thaumatin-like protein isoform 1	thaumatin-like protein isoform 1 [Ficus pumila var. awkeotsang]	Under Expressed	D24
s00033g03912	-1,621	0,002	-	### Not characterized ###:hypothetical protein CICLE_v10026775mg [Citrus clementina]	Under Expressed	D24
s00440g18490	-1,620	0,044	sugar transport protein 14	Sugar transport protein 14 [Morus notabilis]	Under Expressed	D24
s00090g07727	-1,619	0,040	uncharacterized protein LOC103949845 isoform X1	### Not characterized ###:PREDICTED: uncharacterized protein LOC103949845 isoform X1 [Pyrus x bretschneideri]	Under Expressed	D24
s00060g05960	-1,611	0,050	similar to L484_024095	### Not characterized ###:hypothetical protein L484_024095 [Morus notabilis]	Under Expressed	D24
s00513g19933	-1,610	0,039	nudix hydrolase 16	Nudix hydrolase 16 [Morus notabilis] gi|587847999|gb|EXB38302.1|	Under Expressed	D24
s00426g18210	-1,609	0,047	somatic embryogenesis receptor kinase 1	Somatic embryogenesis receptor kinase 1 [Morus notabilis]	Under Expressed	D24

s00280g14730	-1,607	0,004	similar to L484_024389	### Not characterized ###:hypothetical protein L484_024389 [Morus notabilis]	Under Expressed	D24
s00518g20014	-1,600	0,031	gibberellin 3-beta-dioxygenase 4	Gibberellin 3-beta-dioxygenase 4 [Morus notabilis]	Under Expressed	D24
s00062g06049	-1,597	0,005	copper transporter 5	Copper transporter 5 [Morus notabilis] gi|587848712|gb|EXB38971.1|	Under Expressed	D24
s00172g11380	-1,597	0,018	NAC domain-containing protein 29	NAC domain-containing protein 29 [Morus notabilis]	Under Expressed	D24
s00057g05744	-1,596	0,015	1-aminocyclopropane-1-carboxylate oxidase	1-aminocyclopropane-1-carboxylate oxidase [Ficus carica]	Under Expressed	D24
s00788g23866	-1,595	0,001	-	PREDICTED: protein trichome birefringence-like 39 [Populus euphratica]	Under Expressed	D24
s00060g05931	-1,588	0,000	hydroxyproline-rich glycoprotein family protein	Hydroxyproline-rich glycoprotein family protein, putative [Theobroma cacao] gi|508777006|gb|EOY24262.1|	Under Expressed	D24
s00818g24212	-1,584	0,038	S-adenosyl-L-methionine-dependent methyltransferases superfamily protein isoform 1	S-adenosyl-L-methionine-dependent methyltransferases superfamily protein isoform 1 [Theobroma cacao]	Under Expressed	D24
s18340g34755	-1,580	0,030	-	***** No hits found ******	Under Expressed	D24
s00082g07304	-1,574	0,048	S-adenosylmethionine synthase 2	METK2_TOBAC RecName: Full=S-adenosylmethionine synthase 2;Short=AdoMet synthase 2;AltName: Full=Methionine adenosyltransferase 2;Short=MAT 2 gi|38261499|gb|AAR15895.1| S-adenosyl-L-methionine synthetase [Nicotiana tabacum]	Under Expressed	D24
s00599g21354	-1,573	0,035	microtubule-associated protein TORTIFOLIA1	PREDICTED: microtubule-associated protein TORTIFOLIA1 [Malus domestica]	Under Expressed	D24
s00063g06155	-1,569	0,049	momilactone A synthase	Momilactone A synthase [Morus notabilis]	Under Expressed	D24
s00230g13314	-1,567	0,000	transmembrane protein	transmembrane protein, putative [Medicago truncatula]	Under Expressed	D24
s00485g19392	-1,562	0,043	-	### Not characterized ###:PREDICTED: uncharacterized protein LOC105636851 [Jatropha curcas]	Under Expressed	D24
s00220g13011	-1,558	0,002	chitinase-like protein 1	Chitinase-like protein 1 [Morus notabilis]	Under Expressed	D24
s09839g33032	-1,555	0,000	bifunctional glutamate/proline--tRNA ligase	Bifunctional glutamate/proline--tRNA ligase [Morus notabilis]	Under Expressed	D24
s00468g19072	-1,555	0,024	folic acidand derivative biosynthetic process	Folic acidand derivative biosynthetic process [Theobroma cacao]	Under Expressed	D24
s00137g09940	-1,553	0,027	zinc finger CCCH domain-containing protein 29	Zinc finger CCCH domain-containing protein 29 [Morus notabilis]	Under Expressed	D24

s00230g13320	-1,552	0,040	monosaccharide-H+ symporter	monosaccharide-H+ symporter [Datisca glomerata]	Under Expressed	D24
s00142g10161	-1,549	0,001	proton-coupled amino acid transporter 3	PREDICTED: proton-coupled amino acid transporter 3 [Prunus mume]	Under Expressed	D24
s08740g32763	-1,548	0,048	cysteine-rich receptor-like protein kinase 25	Cysteine-rich receptor-like protein kinase 25 [Morus notabilis]	Under Expressed	D24
s00451g18727	-1,547	0,032	transmembrane protein 53	PREDICTED: transmembrane protein 53 [Prunus mume]	Under Expressed	D24
s01832g30066	-1,547	0,031	disease resistance protein	Disease resistance protein [Morus notabilis]	Under Expressed	D24
s00066g06356	-1,543	0,005	auxin transporter-like protein 3	PREDICTED: auxin transporter-like protein 3 [Cucumis melo]	Under Expressed	D24
s00275g14583	-1,543	0,002	3-ketoacyl-CoA synthase 11	3-ketoacyl-CoA synthase 11 [Morus notabilis]	Under Expressed	D24
s00903g25127	-1,542	0,019	glycerol-3-phosphate acyltransferase	glycerol-3-phosphate acyltransferase [Medicago truncatula]	Under Expressed	D24
s08531g32714	-1,540	0,016	-	putative S-adenosylmethionine-dependent methyltransferase [Morus notabilis] gi|587855082|gb|EXB45095.1|	Under Expressed	D24
s00013g02000	-1,537	0,000	SNF1-related protein kinase regulatory subunit gamma-1	SNF1-related protein kinase regulatory subunit gamma-1 [Morus notabilis] gi|587910127|gb|EXB98015.1|	Under Expressed	D24
s00143g10189	-1,534	0,003	uncharacterized protein LOC103330325	### Not characterized ###:PREDICTED: uncharacterized protein LOC103330325 [Prunus mume]	Under Expressed	D24
s00104g08421	-1,533	0,001	amino acid permease 6	PREDICTED: amino acid permease 6 [Nelumbo nucifera]	Under Expressed	D24
s00104g08422	-1,532	0,000	amino acid permease 6	Amino acid permease 6 [Theobroma cacao] gi|508723703|gb|EOY15600.1|	Under Expressed	D24
s07469g32414	-1,532	0,000	similar to L484_006989	### Not characterized ###:hypothetical protein L484_006989 [Morus notabilis]	Under Expressed	D24
s00079g07123	-1,531	0,006	exocyst complex component 7	Exocyst complex component 7 [Morus notabilis]	Under Expressed	D24
s00384g17303	-1,531	0,016	transcription factor bHLH75-like	PREDICTED: transcription factor bHLH75-like [Populus euphratica]	Under Expressed	D24
s00530g20217	-1,528	0,017	-	Methyltransferase-related protein, putative [Theobroma cacao]	Under Expressed	D24
s00606g21471	-1,527	0,000	-	PREDICTED: probable LRR receptor-like serine/threonine-protein kinase At1g53420 [Pyrus x bretschneideri]	Under Expressed	D24
s00111g08770	-1,526	0,044	septum-promoting GTP-binding protein 1	Septum-promoting GTP-binding protein 1 [Morus notabilis]	Under Expressed	D24
s00123g09344	-1,525	0,003	subtilisin-like protease	Subtilisin-like protease [Morus notabilis]	Under Expressed	D24

s00098g08156	-1,524	0,001	RING-H2 finger protein ATL1	RING-H2 finger protein ATL1 [Morus notabilis]	Under Expressed	D24
s00057g05743	-1,523	0,030	-	***** No hits found ******	Under Expressed	D24
s00001g00232	-1,522	0,003	periplasmic beta-glucosidase precursor	Periplasmic beta-glucosidase precursor, putative [Ricinus communis]	Under Expressed	D24
s01464g28874	-1,518	0,039	alpha-galactosidase/alpha-n-acetylgalactosaminidase	alpha-galactosidase/alpha-n-acetylgalactosaminidase, putative [Ricinus communis] gi|223535102|gb|EEF36784.1|	Under Expressed	D24
s09716g33002	-1,514	0,004	-	PLATZ transcription factor family protein [Medicago truncatula]	Under Expressed	D24
s07613g32462	-1,512	0,009	-	4-nitrophenylphosphatase, putative [Ricinus communis]	Under Expressed	D24
s00012g01785	-1,512	0,001	phenylalanine ammonia lyase 1	phenylalanine ammonia lyase 1 [Morus alba]	Under Expressed	D24
s00355g16616	-1,511	0,028	alkaline/neutral invertase CINV2	PREDICTED: alkaline/neutral invertase CINV2 [Prunus mume]	Under Expressed	D24
s00304g15349	-1,509	0,028	actin cross-linking protein	Actin cross-linking protein, putative [Theobroma cacao]	Under Expressed	D24
s13987g33933	-1,508	0,049	transcription factor	Transcription factor [Morus notabilis] gi|587846520|gb|EXB36998.1|	Under Expressed	D24
s00337g16181	-1,477	0,000	amino acid transporter	amino acid transporter, putative [Ricinus communis]	Under Expressed	D24
s02564g30953	-1,454	0,042	-	***** No hits found ******	Under Expressed	D24
s00040g04473	-1,443	0,026	similar to L484_016065	### Not characterized ###:hypothetical protein L484_016065 [Morus notabilis]	Under Expressed	D24
s00021g02811	-1,440	0,000	zinc finger CCCH domain-containing protein 49	Zinc finger CCCH domain-containing protein 49 [Morus notabilis]	Under Expressed	D24
s02094g30518	-1,439	0,002	carboxylesterase 8	putative carboxylesterase 8 [Morus notabilis]	Under Expressed	D24
s00227g13254	-1,433	0,008	-	***** No hits found ******	Under Expressed	D24
s00104g08416	-1,433	0,047	formin-like protein 18	Formin-like protein 18 [Theobroma cacao]	Under Expressed	D24
s00601g21386	-1,430	0,000	3-ketoacyl-CoA synthase 6	3-ketoacyl-CoA synthase 6 [Theobroma cacao]	Under Expressed	D24
s00010g01616	-1,424	0,004	homeodomain-leucine zipper protein HD4	Homeodomain-leucine zipper protein HD4 [Theobroma cacao]	Under Expressed	D24
s00158g10832	-1,420	0,044	LRR receptor-like protein kinase m4	LRR receptor-like protein kinase m4 [Malus domestica]	Under Expressed	D24
s00258g14121	-1,411	0,000	beta-hexosaminidase 2	PREDICTED: beta-hexosaminidase 2 [Prunus mume]	Under Expressed	D24
s00522g20088	-1,407	0,049	mapkinase substrate	mapkinase substrate [Acacia mangium]	Under Expressed	D24

s00215g12861	-1,407	0,032	pectinesterase/pectinesterase inhibitor PPE8B	Pectinesterase/pectinesterase inhibitor PPE8B [Morus notabilis]	Under Expressed	D24
s00803g24018	-1,401	0,008	cytidine deaminase	Cytidine deaminase [Morus notabilis] gi|587848572|gb|EXB38831.1|	Under Expressed	D24
s00470g19112	-1,401	0,000	phosphatase 2C family protein isoform 1	Phosphatase 2C family protein isoform 1 [Theobroma cacao]	Under Expressed	D24
s00029g03549	-1,399	0,001	triacylglycerol lipase	triacylglycerol lipase, putative [Ricinus communis]	Under Expressed	D24
s01488g28983	-1,397	0,000	-	PREDICTED: zinc finger homeobox protein 4-like [Citrus sinensis]	Under Expressed	D24
s00192g12068	-1,395	0,001	similar to L484_018088	### Not characterized ###:hypothetical protein L484_018088 [Morus notabilis]	Under Expressed	D24
s00006g01047	-1,393	0,001	phosphoenolpyruvate/phosphate translocator 2	Phosphoenolpyruvate/phosphate translocator 2 [Morus notabilis]	Under Expressed	D24
s00791g23909	-1,388	0,000	receptor-like protein kinase	putative receptor-like protein kinase [Morus notabilis]	Under Expressed	D24
s23147g35493	-1,384	0,042	phenylalanine ammonia-lyase	Phenylalanine ammonia-lyase [Morus notabilis]	Under Expressed	D24
s00049g05153	-1,383	0,000	inactive receptor kinase	putative inactive receptor kinase [Morus notabilis]	Under Expressed	D24
s00046g04925	-1,380	0,001	S-adenosyl-L-methionine-dependent methyltransferases superfamily protein	S-adenosyl-L-methionine-dependent methyltransferases superfamily protein [Theobroma cacao] gi|508703717|gb|EOX95613.1|	Under Expressed	D24
s00266g14376	-1,377	0,024	similar to L484_014675	### Not characterized ###:hypothetical protein L484_014675 [Morus notabilis]	Under Expressed	D24
s00462g18964	-1,375	0,000	RING-H2 finger protein ATL54	RING-H2 finger protein ATL54 [Morus notabilis]	Under Expressed	D24
s00215g12862	-1,374	0,012	pectinesterase/pectinesterase inhibitor PPE8B	Pectinesterase/pectinesterase inhibitor PPE8B [Morus notabilis]	Under Expressed	D24
s00105g08440	-1,372	0,003	soluble inorganic pyrophosphatase	Soluble inorganic pyrophosphatase [Morus notabilis]	Under Expressed	D24
s01074g26646	-1,372	0,039	alpha/beta hydrolase domain-containing protein 17C	PREDICTED: alpha/beta hydrolase domain-containing protein 17C [Prunus mume]	Under Expressed	D24
s00009g01444	-1,368	0,028	UBX domain-containing protein 1	UBX domain-containing protein 1 [Morus notabilis]	Under Expressed	D24
s00202g12438	-1,364	0,042	disease resistance RPP13-like protein 4	Disease resistance RPP13-like protein 4 [Morus notabilis]	Under Expressed	D24
s00037g04258	-1,362	0,000	isoflavone 2'-hydroxylase	Isoflavone 2'-hydroxylase [Morus notabilis]	Under Expressed	D24
s00009g01547	-1,360	0,000	calmodulin-like protein 1	Calmodulin-like protein 1 [Morus notabilis]	Under Expressed	D24
s00465g19016	-1,360	0,003	-	***** No hits found ******	Under Expressed	D24

s00258g14124	-1,359	0,003	similar to VITISV_036996	### Not characterized ###:hypothetical protein VITISV_036996 [Vitis vinifera]	Under Expressed	D24
s00055g05613	-1,356	0,000	transcription factor	Transcription factor [Morus notabilis] gi|587885720|gb|EXB74577.1| Transcription factor [Morus notabilis]	Under Expressed	D24
s00082g07317	-1,349	0,045	uncharacterized membrane protein YMR155W-like	PREDICTED: uncharacterized membrane protein YMR155W-like [Citrus sinensis]	Under Expressed	D24
s00054g05518	-1,348	0,007	3-hydroxy-3-methylglutaryl-coenzyme A reductase 2	3-hydroxy-3-methylglutaryl-coenzyme A reductase 2 [Morus alba]	Under Expressed	D24
s00176g11502	-1,346	0,000	-	PREDICTED: kinesin-like protein KIF21A [Populus euphratica]	Under Expressed	D24
s05943g31999	-1,341	0,014	1-aminocyclopropane-1-carboxylate oxidase 3	1-aminocyclopropane-1-carboxylate oxidase 3 [Morus notabilis]	Under Expressed	D24
s00055g05554	-1,340	0,013	similar to L484_026207	### Not characterized ###:hypothetical protein L484_026207 [Morus notabilis]	Under Expressed	D24
s00348g16432	-1,339	0,034	serine/threonine-protein kinase	putative serine/threonine-protein kinase [Morus notabilis]	Under Expressed	D24
s00116g09016	-1,339	0,001	galacturonosyltransferase-like 3	putative galacturonosyltransferase-like 3 [Morus notabilis]	Under Expressed	D24
s00144g10278	-1,328	0,027	-	PREDICTED: protein AUXIN-REGULATED GENE INVOLVED IN ORGAN SIZE-like [Pyrus x bretschneideri]	Under Expressed	D24
s00232g13380	-1,320	0,000	-	***** No hits found ******	Under Expressed	D24
s00309g15501	-1,308	0,029	GTP-binding protein	GTP-binding protein [Morus notabilis] gi|587866258|gb|EXB55736.1|	Under Expressed	D24
s00681g22520	-1,301	0,024	-	PREDICTED: protein COBRA-like [Pyrus x bretschneideri]	Under Expressed	D24
s00376g17145	-1,298	0,000	pectinesterase/pectinesterase inhibitor 34	putative pectinesterase/pectinesterase inhibitor 34 [Morus notabilis] gi|587895730|gb|EXB84224.1|	Under Expressed	D24
s00327g15964	-1,295	0,041	-	PREDICTED: protein trichome birefringence-like 35 [Jatropha curcas]	Under Expressed	D24
s00102g08339	-1,292	0,028	GDSL esterase/lipase 1	GDSL esterase/lipase 1 [Morus notabilis]	Under Expressed	D24
s00402g17710	-1,291	0,029	transporter MCH1	putative transporter MCH1 [Morus notabilis]	Under Expressed	D24
s00283g14810	-1,290	0,004	F-box/LRR-repeat protein At4g29420	PREDICTED: F-box/LRR-repeat protein At4g29420 [Jatropha curcas]	Under Expressed	D24
s00136g09914	-1,289	0,022	naringenin,2-oxoglutarate 3-dioxygenase	Naringenin,2-oxoglutarate 3-dioxygenase [Morus notabilis]	Under Expressed	D24
s01268g27931	-1,289	0,033	auxin-induced protein 5NG4	Auxin-induced protein 5NG4 [Morus notabilis]	Under Expressed	D24

s00064g06231	-1,284	0,004	carboxylesterase 15	PREDICTED: probable carboxylesterase 15 [Malus domestica]	Under Expressed	D24
s00030g03653	-1,280	0,019	adenylyl-sulfate kinase 3 isoform X2	PREDICTED: adenylyl-sulfate kinase 3 isoform X2 [Fragaria vesca subsp. vesca]	Under Expressed	D24
s01354g28377	-1,278	0,013	-	PREDICTED: transcription factor bHLH128 [Malus domestica]	Under Expressed	D24
s00328g15987	-1,277	0,007	serine/threonine-protein kinase Aurora-1	Serine/threonine-protein kinase Aurora-1 [Morus notabilis]	Under Expressed	D24
s00198g12281	-1,275	0,018	early nodulin-like protein 2	Early nodulin-like protein 2 [Morus notabilis]	Under Expressed	D24
s00037g04204	-1,271	0,022	-	***** No hits found ******	Under Expressed	D24
s00431g18308	-1,265	0,003	cytochrome P450 85A	Cytochrome P450 85A [Morus notabilis] gi|587888974|gb|EXB77660.1|	Under Expressed	D24
s00864g24685	-1,264	0,011	F-box/kelch-repeat protein	F-box/kelch-repeat protein [Morus notabilis]	Under Expressed	D24
s00084g07400	-1,262	0,042	NAC domain-containing protein 100	NAC domain-containing protein 100 [Morus notabilis]	Under Expressed	D24
s00077g06999	-1,257	0,046	-	PREDICTED: alpha-galactosidase-like [Pyrus x bretschneideri]	Under Expressed	D24
s00064g06216	-1,257	0,003	fasciclin-like arabinogalactan protein 17	PREDICTED: fasciclin-like arabinogalactan protein 17 [Populus euphratica] gi|743898520|ref|XP_011042550.1|	Under Expressed	D24
s00458g18873	-1,255	0,011	similar to L484_018723	### Not characterized ###:hypothetical protein L484_018723 [Morus notabilis]	Under Expressed	D24
s00635g21905	-1,253	0,026	reticulon-like protein	Reticulon-like protein [Morus notabilis]	Under Expressed	D24
s00791g23906	-1,251	0,000	receptor-like protein kinase At5g39020	PREDICTED: probable receptor-like protein kinase At5g39020 [Pyrus x bretschneideri]	Under Expressed	D24
s00137g09958	-1,248	0,000	cytochrome P450 90A1	PREDICTED: cytochrome P450 90A1 [Prunus mume]	Under Expressed	D24
s00280g14734	-1,248	0,000	plasma intrinsic protein 2,2	plasma intrinsic protein 2,2 [Juglans regia]	Under Expressed	D24
s01347g28339	-1,246	0,006	alpha,alpha-trehalose-phosphate synthase [UDP-forming] 7	putative alpha,alpha-trehalose-phosphate synthase [UDP-forming] 7 [Morus notabilis] gi|587904173|gb|EXB92374.1|	Under Expressed	D24
s00023g03035	-1,243	0,045	uncharacterized protein LOC105351441	### Not characterized ###:PREDICTED: uncharacterized protein LOC105351441 [Fragaria vesca subsp. vesca]	Under Expressed	D24
s00311g15565	-1,242	0,003	FK506-binding protein 4-like isoform X1	PREDICTED: FK506-binding protein 4-like isoform X1 [Brassica napus]	Under Expressed	D24
s05538g31894	-1,238	0,050	glutamate decarboxylase 1	Glutamate decarboxylase 1 [Morus notabilis]	Under Expressed	D24

s00134g09807	-1,237	0,005	-	### Not characterized ###:PREDICTED: uncharacterized protein LOC100265438 isoform X1 [Vitis vinifera] gi|296086582|emb|CBI32217.3| unnamed protein product [Vitis vinifera]	Under Expressed	D24
s00025g03207	-1,235	0,003	uncharacterized protein LOC106774881	### Not characterized ###:PREDICTED: uncharacterized protein LOC106774881 [Vigna radiata var. radiata]	Under Expressed	D24
s03023g31136	-1,232	0,004	-	UDP-glucosyltransferase, putative [Ricinus communis]	Under Expressed	D24
s00053g05435	-1,228	0,048	polyol transporter 5	Polyol transporter 5 [Morus notabilis] gi|587891305|gb|EXB79938.1|	Under Expressed	D24
s00537g20336	-1,225	0,007	cyclic nucleotide-gated ionchannel 14	putative cyclic nucleotide-gated ionchannel 14 [Morus notabilis]	Under Expressed	D24
s00187g11939	-1,223	0,022	NAC domain-containing protein 72	NAC domain-containing protein 72 [Morus notabilis]	Under Expressed	D24
s00852g24563	-1,221	0,000	-	### Not characterized ###:hypothetical protein L484_008140 [Morus notabilis]	Under Expressed	D24
s00573g20950	-1,219	0,006	cytochrome P450 71A1	Cytochrome P450 71A1 [Morus notabilis] gi|587867550|gb|EXB56947.1| Cytochrome P450 71A1 [Morus notabilis]	Under Expressed	D24
s00162g11005	-1,218	0,003	glycosyltransferase family protein 64 protein C5	PREDICTED: glycosyltransferase family protein 64 protein C5 [Fragaria vesca subsp. vesca]	Under Expressed	D24
s00982g25868	-1,217	0,003	transcription factor RF2b	Transcription factor RF2b [Morus notabilis]	Under Expressed	D24
s00384g17300	-1,213	0,000	mannose-6-phosphate isomerase	putative mannose-6-phosphate isomerase [Morus notabilis]	Under Expressed	D24
s00427g18234	-1,210	0,014	uncharacterized protein L484_005224	### Not characterized ###:Uncharacterized protein L484_005224 [Morus notabilis]	Under Expressed	D24
s03565g31304	-1,209	0,003	MLO-like protein 6	PREDICTED: MLO-like protein 6 [Populus euphratica]	Under Expressed	D24
s00150g10531	-1,206	0,001	patatin group A-3	Patatin group A-3 [Morus notabilis] gi|587891462|gb|EXB80085.1|	Under Expressed	D24
s00791g23908	-1,205	0,036	-	PREDICTED: probable receptor-like protein kinase At1g67000, partial [Vigna radiata var. radiata]	Under Expressed	D24
s01598g29417	-1,201	0,000	-	PREDICTED: protein MIZU-KUSSEI 1 [Vitis vinifera]	Under Expressed	D24
s00008g01311	-1,197	0,044	F-box/kelch-repeat protein SKIP6	F-box/kelch-repeat protein SKIP6 [Morus notabilis]	Under Expressed	D24
s04767g31683	-1,197	0,000	membrane lipoprotein lipid attachment site-like protein	membrane lipoprotein lipid attachment site-like protein, putative [Medicago truncatula] gi|657373996|gb|KEH19601.1|	Under Expressed	D24
s07483g32420	-1,197	0,042	seven transmembrane MLO family protein	Seven transmembrane MLO family protein [Theobroma cacao]	Under Expressed	D24

s00142g10154	-1,196	0,022	transporter	putative transporter [Morus notabilis] gi|587907084|gb|EXB95111.1|	Under Expressed	D24
s00679g22496	-1,195	0,045	guanylate kinase	Guanylate kinase [Morus notabilis] gi|587863124|gb|EXB52902.1|	Under Expressed	D24
s01096g26796	-1,183	0,000	BOI-related E3 ubiquitin-protein ligase 1	PREDICTED: BOI-related E3 ubiquitin-protein ligase 1 [Fragaria vesca subsp. vesca]	Under Expressed	D24
s00300g15255	-1,178	0,000	U-box domain-containing protein 18	U-box domain-containing protein 18 [Morus notabilis]	Under Expressed	D24
s00909g25195	-1,174	0,040	cysteine proteinase RD21a	Cysteine proteinase RD21a [Morus notabilis]	Under Expressed	D24
s00052g05349	-1,169	0,028	pectinesterase 1	Pectinesterase 1 [Morus notabilis] gi|587860032|gb|EXB49963.1|	Under Expressed	D24
s00444g18587	-1,168	0,008	F-box protein	F-box protein [Morus notabilis] gi|587866374|gb|EXB55843.1|	Under Expressed	D24
s00094g07941	-1,168	0,028	tubulin beta-1 chain	Tubulin beta-1 chain [Morus notabilis] gi|587846916|gb|EXB37356.1|	Under Expressed	D24
s00812g24128	-1,161	0,009	-	PREDICTED: protein MIZU-KUSSEI 1 [Malus domestica]	Under Expressed	D24
s00063g06126	-1,154	0,038	aspartic proteinase nepenthesin-2	Aspartic proteinase nepenthesin-2 [Morus notabilis]	Under Expressed	D24
s00117g09083	-1,153	0,005	L-type lectin-domain containing receptor kinase S.1	L-type lectin-domain containing receptor kinase S.1 [Morus notabilis] gi|587905385|gb|EXB93547.1|	Under Expressed	D24
s00038g04285	-1,152	0,003	21 kDa protein	PREDICTED: 21 kDa protein [Prunus mume]	Under Expressed	D24
s00175g11474	-1,150	0,012	GDP-mannose 4,6 dehydratase 2	GDP-mannose 4,6 dehydratase 2 [Morus notabilis]	Under Expressed	D24
s00103g08359	-1,148	0,039	glutamate decarboxylase 1 -like protein	Glutamate decarboxylase 1 -like protein [Gossypium arboreum]	Under Expressed	D24
s00035g04110	-1,148	0,050	polygalacturonase	putative polygalacturonase [Morus notabilis]	Under Expressed	D24
s00321g15827	-1,145	0,001	ammonium transporter 2	Ammonium transporter 2 [Morus notabilis]	Under Expressed	D24
s00014g02048	-1,142	0,003	glycosyl hydrolases family 32 protein isoform 1	Glycosyl hydrolases family 32 protein isoform 1 [Theobroma cacao]	Under Expressed	D24
s00053g05417	-1,142	0,044	dynein light chain LC6, flagellar outerarm	Dynein light chain LC6, flagellar outerarm [Morus notabilis]	Under Expressed	D24
s00556g20662	-1,138	0,000	MRNA, cds, clone: RAFL24-31-B07	MRNA, cds, clone: RAFL24-31-B07, putative [Theobroma cacao]	Under Expressed	D24
s01049g26466	-1,137	0,038	-	CCL1 [Humulus lupulus]	Under Expressed	D24
s00059g05863	-1,135	0,011	fasciclin-like arabinogalactan protein 10	PREDICTED: fasciclin-like arabinogalactan protein 10 [Fragaria vesca subsp. vesca]	Under Expressed	D24
s00151g10556	-1,125	0,000	GATA transcription factor 5	GATA transcription factor 5 [Morus notabilis]	Under Expressed	D24
s00764g23570	-1,122	0,017	DUF630 family protein	DUF630 family protein [Medicago truncatula]	Under Expressed	D24

s00077g07000	-1,122	0,010	alpha-galactosidase 3	PREDICTED: alpha-galactosidase 3 [Jatropha curcas]	Under Expressed	D24
s00673g22417	-1,119	0,046	sodium-coupled neutral amino acid transporter 4	PREDICTED: sodium-coupled neutral amino acid transporter 4 [Pyrus x bretschneideri]	Under Expressed	D24
s00289g14963	-1,115	0,010	OPA3-like protein	PREDICTED: OPA3-like protein [Vitis vinifera]	Under Expressed	D24
s00007g01208	-1,114	0,003	-	### Not characterized ###:hypothetical protein [Plasmodium vivax Sal-1]	Under Expressed	D24
s02101g30525	-1,111	0,001	agmatine coumaroyltransferase	Agmatine coumaroyltransferase [Morus notabilis]	Under Expressed	D24
s00791g23907	-1,100	0,039	-	putative receptor-like protein kinase [Morus notabilis]	Under Expressed	D24
s00193g12132	-1,098	0,034	ranBP2-type zinc finger protein At1g67325-like isoform X1	PREDICTED: ranBP2-type zinc finger protein At1g67325-like isoform X1 [Vitis vinifera]	Under Expressed	D24
s00227g13253	-1,093	0,014	bidirectional sugar transporter SWEET1 -like protein	Bidirectional sugar transporter SWEET1 -like protein [Gossypium arboreum]	Under Expressed	D24
s00107g08555	-1,092	0,032	serine-threonine protein kinase, plant-type	serine-threonine protein kinase, plant-type, putative [Ricinus communis] gi|223549901|gb|EEF51388.1|	Under Expressed	D24
s00053g05391	-1,091	0,000	auxin-responsive protein IAA27	Auxin-responsive protein IAA27 [Morus notabilis]	Under Expressed	D24
s00384g17299	-1,087	0,005	elongator complex protein 5-like	PREDICTED: elongator complex protein 5-like [Citrus sinensis]	Under Expressed	D24
s00030g03636	-1,087	0,020	PRA1 family protein B4	PREDICTED: PRA1 family protein B4 [Malus domestica]	Under Expressed	D24
s08986g32819	-1,087	0,037	-	***** No hits found ******	Under Expressed	D24
s00586g21128	-1,086	0,001	RING-H2 finger protein ATL13	RING-H2 finger protein ATL13 [Morus notabilis]	Under Expressed	D24
s00756g23472	-1,086	0,013	-	SAUR-like auxin-responsive protein family [Theobroma cacao]	Under Expressed	D24
s00217g12920	-1,083	0,024	-	Protein ETHYLENE INSENSITIVE 3 [Morus notabilis]	Under Expressed	D24
s00225g13184	-1,083	0,001	arogenate dehydratase/prephenate dehydratase 6	Arogenate dehydratase/prephenate dehydratase 6 [Morus notabilis]	Under Expressed	D24
s10351g33151	-1,074	0,050	fructokinase-4	putative fructokinase-4 [Morus notabilis]	Under Expressed	D24
s00252g13966	-1,069	0,001	-	protein phosphatase-2c, putative [Ricinus communis]	Under Expressed	D24
s00382g17278	-1,068	0,008	chaperone protein ClpB3, chloroplastic	PREDICTED: chaperone protein ClpB3, chloroplastic [Malus domestica]	Under Expressed	D24
s00743g23313	-1,065	0,000	amidophosphoribosyltransferase	Amidophosphoribosyltransferase [Morus notabilis]	Under Expressed	D24

s00141g10115	-1,065	0,001	solute carrier family 35 member F1-like	PREDICTED: solute carrier family 35 member F1-like [Prunus mume]	Under Expressed	D24
s00021g02844	-1,064	0,001	-	PREDICTED: protein REVERSION-TO-ETHYLENE SENSITIVITY1 [Prunus mume]	Under Expressed	D24
s00314g15630	-1,063	0,024	auxin:hydrogen symporter	auxin:hydrogen symporter, putative [Ricinus communis]	Under Expressed	D24
s00068g06434	-1,063	0,000	cellulase protein	Cellulase protein [Theobroma cacao] gi|508708807|gb|EOY00704.1|	Under Expressed	D24
s00076g06936	-1,062	0,023	ubiquitin-conjugating enzyme E2-17 kDa	PREDICTED: ubiquitin-conjugating enzyme E2-17 kDa [Sesamum indicum]	Under Expressed	D24
s00314g15629	-1,061	0,008	-	Uncharacterized transporter [Morus notabilis]	Under Expressed	D24
s00344g16346	-1,058	0,007	similar to L484_028070	### Not characterized ###:hypothetical protein L484_028070 [Morus notabilis]	Under Expressed	D24
s00013g01905	-1,056	0,020	baculoviral IAP repeat-containing protein 7-A	Baculoviral IAP repeat-containing protein 7-A [Morus notabilis]	Under Expressed	D24
s00029g03595	-1,054	0,006	-	PREDICTED: protein EXORDIUM-like 2 [Populus euphratica]	Under Expressed	D24
s00490g19494	-1,053	0,022	G-type lectin S-receptor-like serine/threonine-protein kinase	G-type lectin S-receptor-like serine/threonine-protein kinase [Morus notabilis] gi|587867998|gb|EXB57371.1|	Under Expressed	D24
s00028g03511	-1,050	0,011	sericin-2	PREDICTED: sericin-2 [Prunus mume]	Under Expressed	D24
s00028g03464	-1,047	0,000	aspartic proteinase nepenthesin-2	Aspartic proteinase nepenthesin-2 [Morus notabilis]	Under Expressed	D24
s00530g20219	-1,043	0,019	CBL-interacting serine/threonine-protein kinase 11	CBL-interacting serine/threonine-protein kinase 11 [Morus notabilis] gi|587932508|gb|EXC19556.1|	Under Expressed	D24
s00477g19236	-1,029	0,044	-	Calcium-dependent lipid-binding family protein, putative [Theobroma cacao] gi|508711108|gb|EOY03005.1|	Under Expressed	D24
s00351g16516	-1,028	0,006	LRR receptor-like serine/threonine-protein kinase At4g36180	PREDICTED: probable LRR receptor-like serine/threonine-protein kinase At4g36180 [Malus domestica]	Under Expressed	D24
s00274g14555	-1,025	0,000	ribose-phosphate pyrophosphokinase 1	Ribose-phosphate pyrophosphokinase 1 [Morus notabilis]	Under Expressed	D24
s00253g13994	-1,025	0,024	-	PREDICTED: protein NDR1-like [Fragaria vesca subsp. vesca]	Under Expressed	D24
s00044g04757	-1,023	0,000	aluminum induced protein with YGL and LRDR motifs	Aluminum induced protein with YGL and LRDR motifs [Theobroma cacao]	Under Expressed	D24
s00242g13685	-1,021	0,039	epoxide hydrolase 3-like	PREDICTED: epoxide hydrolase 3-like [Malus domestica]	Under Expressed	D24

s00122g09302	-1,019	0,004	U-box domain-containing protein 16	U-box domain-containing protein 16 [Morus notabilis]	Under Expressed	D24
s00537g20337	-1,017	0,040	cellulose-synthase-like C5	Cellulose-synthase-like C5 [Theobroma cacao]	Under Expressed	D24
s00657g22213	-1,010	0,050	hydroxycinnamoyl-Coenzyme A shikimate/quinate hydroxycinnamoyltransferase	Hydroxycinnamoyl-Coenzyme A shikimate/quinate hydroxycinnamoyltransferase [Morus notabilis]	Under Expressed	D24
s00606g21470	-1,010	0,016	-	PREDICTED: probable LRR receptor-like serine/threonine-protein kinase At1g53430-like isoform X5 [Citrus sinensis]	Under Expressed	D24
s00975g25808	-1,010	0,019	UPF0481 protein At3g47200-like	PREDICTED: UPF0481 protein At3g47200-like [Sesamum indicum]	Under Expressed	D24
s16858g34475	-1,010	0,001	GDSL esterase/lipase 1	GDSL esterase/lipase 1 [Morus notabilis]	Under Expressed	D24
s00077g07008	-1,008	0,042	transcription factor RF2b	Transcription factor RF2b [Morus notabilis]	Under Expressed	D24
s00365g16891	-1,003	0,001	mitochondrial substrate carrier family protein P	PREDICTED: mitochondrial substrate carrier family protein P [Prunus mume]	Under Expressed	D24
s00694g22691	5,585	0,000	-	### Not characterized ###:hypothetical protein PRUPE_ppa023320mg [Prunus persica]	Over Expressed	D48
s00067g06362	5,347	0,020	-	PREDICTED: non-specific lipid-transfer protein 2-like [Cucumis melo]	Over Expressed	D48
s00046g04909	4,988	0,000	cytochrome P450 78A3	Cytochrome P450 78A3 [Morus notabilis] gi|587895348|gb|EXB83849.1|	Over Expressed	D48
s00614g21595	4,905	0,008	auxin-induced protein 5NG4	Auxin-induced protein 5NG4 [Morus notabilis]	Over Expressed	D48
s00385g17333	4,767	0,002	-	PREDICTED: mybfamily transcription factor APL-like isoform X2 [Prunus mume]	Over Expressed	D48
s00374g17096	4,509	0,046	-	### Not characterized ###:PREDICTED: uncharacterized protein LOC101293734 [Fragaria vesca subsp. vesca]	Over Expressed	D48
s00442g18535	4,487	0,000	RING-H2 finger protein ATL66	RING-H2 finger protein ATL66 [Morus notabilis]	Over Expressed	D48
s17202g34542	4,465	0,048	-	***** No hits found ******	Over Expressed	D48
s00019g02612	4,370	0,004	-	PREDICTED: pathogenesis-related protein 1A-like [Eucalyptus grandis]	Over Expressed	D48
s07957g32556	4,346	0,013	-	Cytochrome P450 [Morus notabilis] gi|587893952|gb|EXB82484.1|	Over Expressed	D48
s00439g18470	4,229	0,005	-	Vicilin-like antimicrobial peptides 2-2 [Morus notabilis]	Over Expressed	D48
s00756g23469	4,229	0,000	glucose-1-phosphate adenylyltransferase large subunit 3	Glucose-1-phosphate adenylyltransferase large subunit 3 [Morus notabilis] gi|587846684|gb|EXB37149.1|	Over Expressed	D48
s18644g34804	4,216	0,016	-	***** No hits found ******	Over Expressed	D48
s03046g31144	4,209	0,047	pirin-like protein	PREDICTED: pirin-like protein [Glycine max]	Over Expressed	D48

s00046g04958	4,199	0,000	-	Histone deacetylase 18 -like protein [Gossypium arboreum]	Over Expressed	D48
s21650g35284	4,165	0,000	-	***** No hits found ******	Over Expressed	D48
s17173g34534	3,967	0,029	-	### Not characterized ###:hypothetical protein VITISV_022441 [Vitis vinifera]	Over Expressed	D48
s00099g08224	3,906	0,001	CAP160 protein	CAP160 protein, putative [Theobroma cacao]	Over Expressed	D48
s01243g27792	3,761	0,000	-	PREDICTED: wall-associated receptor kinase-like 22 [Prunus mume]	Over Expressed	D48
s00616g21632	3,743	0,000	delta-1-pyrroline-5-carboxylate synthase	Delta-1-pyrroline-5-carboxylate synthase [Morus notabilis]	Over Expressed	D48
s00197g12247	3,709	0,002	-	***** No hits found ******	Over Expressed	D48
s00197g12257	3,609	0,041	fatty acyl-CoA reductase 3	Fatty acyl-CoA reductase 3 [Morus notabilis]	Over Expressed	D48
s00768g23620	3,596	0,003	AC093178_1 Putative polyprotein	AC093178_1 Putative polyprotein [Oryza sativa Japonica Group]	Over Expressed	D48
s13193g33777	3,579	0,046	cytochrome P450 71A1	Cytochrome P450 71A1 [Morus notabilis] gi|587906393|gb|EXB94465.1|	Over Expressed	D48
s00065g06261	3,514	0,000	EID1-like F-box protein 3	PREDICTED: EID1-like F-box protein 3 [Vitis vinifera]	Over Expressed	D48
s00785g23824	3,508	0,000	isoflavone 2'-hydroxylase	Isoflavone 2'-hydroxylase [Morus notabilis]	Over Expressed	D48
s00018g02534	3,504	0,001	-	PREDICTED: putative late blight resistance protein homolog R1B-19 [Populus euphratica] gi|743852780|ref|XP_011029353.1|	Over Expressed	D48
s15768g34268	3,487	0,013	copper-transporting ATPase 3	Putative copper-transporting ATPase 3 [Morus notabilis]	Over Expressed	D48
s00068g06438	3,473	0,000	cytochrome P450	Cytochrome P450 [Morus notabilis] gi|587893952|gb|EXB82484.1|	Over Expressed	D48
s00773g23687	3,464	0,007	polyprotein	polyprotein [Ananas comosus]	Over Expressed	D48
s00579g21049	3,460	0,007	-	***** No hits found ******	Over Expressed	D48
s00158g10845	3,449	0,030	similar to L484_015065	### Not characterized ###:hypothetical protein L484_015065 [Morus notabilis]	Over Expressed	D48
s00002g00406	3,441	0,000	serine/arginine repetitive matrix protein 2 isoform 1	Serine/arginine repetitive matrix protein 2 isoform 1 [Theobroma cacao] gi|508702714|gb|EOX94610.1|	Over Expressed	D48
s01136g27085	3,293	0,013	expansin-like protein	Expansin-like protein [Morus notabilis] gi|587914306|gb|EXC02085.1|	Over Expressed	D48
s00003g00605	3,252	0,000	-	### Not characterized ###:hypothetical protein L484_007652 [Morus notabilis]	Over Expressed	D48

s00002g00421	3,172	0,000	heat stress transcription factor A-6b	Heat stress transcription factor A-6b [Morus notabilis]	Over Expressed	D48
s00287g14908	3,153	0,000	-	***** No hits found ******	Over Expressed	D48
s21292g35232	3,141	0,006	cytochrome P450 704C1-like	PREDICTED: cytochrome P450 704C1-like [Pyrus x bretschneideri]	Over Expressed	D48
s00350g16497	3,140	0,040	-	***** No hits found ******	Over Expressed	D48
s01059g26534	3,130	0,047	-	***** No hits found ******	Over Expressed	D48
s03630g31324	3,037	0,040	UDP-glycosyltransferase 87A1	UDP-glycosyltransferase 87A1 [Morus notabilis]	Over Expressed	D48
s00509g19844	3,007	0,007	-	***** No hits found ******	Over Expressed	D48
s27004g36012	3,007	0,019	-	Cellulose synthase family protein isoform 1 [Theobroma cacao]	Over Expressed	D48
s00036g04168	2,969	0,020	-	Putative Myb family transcription factor [Morus notabilis]	Over Expressed	D48
s00139g10024	2,965	0,000	highly ABA-induced PP2C gene 2 isoform 1	Highly ABA-induced PP2C gene 2 isoform 1 [Theobroma cacao]	Over Expressed	D48
s00609g21528	2,959	0,000	delta-1-pyrroline-5-carboxylate synthase	Delta-1-pyrroline-5-carboxylate synthase [Morus notabilis]	Over Expressed	D48
s00006g01156	2,907	0,000	UPF0392 protein RCOM_0530710	PREDICTED: UPF0392 protein RCOM_0530710 [Jatropha curcas]	Over Expressed	D48
s01689g29686	2,894	0,047	-	***** No hits found ******	Over Expressed	D48
s00488g19437	2,852	0,000	uncharacterized membrane protein At3g27390 isoform X2	PREDICTED: uncharacterized membrane protein At3g27390 isoform X2 [Gossypium raimondii]	Over Expressed	D48
s00243g13708	2,834	0,001	1-aminocyclopropane-1-carboxylate oxidase-1-like protein	1-aminocyclopropane-1-carboxylate oxidase-1-like protein [Morus notabilis] gi|587929255|gb|EXC16422.1|	Over Expressed	D48
s00362g16800	2,814	0,000	AFP homolog 2	PREDICTED: AFP homolog 2 [Prunus mume]	Over Expressed	D48
s00351g16511	2,813	0,005	acetyltransferase	putative acetyltransferase [Morus notabilis]	Over Expressed	D48
s00163g11009	2,781	0,017	galactinol synthase 2-like	PREDICTED: galactinol synthase 2-like [Fragaria vesca subsp. vesca]	Over Expressed	D48
s00350g16495	2,774	0,002	inactive purple acid phosphatase 27	putative inactive purple acid phosphatase 27 [Morus notabilis]	Over Expressed	D48
s00005g00867	2,755	0,013	-	PREDICTED: protein SRG1 [Fragaria vesca subsp. vesca]	Over Expressed	D48
s00551g20581	2,744	0,008	similar to VITISV_024746	### Not characterized ###:hypothetical protein VITISV_024746 [Vitis vinifera]	Over Expressed	D48
s00713g22919	2,691	0,013	galactinol--sucrose galactosyltransferase 2-like isoform X1	PREDICTED: probable galactinol--sucrose galactosyltransferase 2-like isoform X1 [Citrus sinensis]	Over Expressed	D48
s00515g19963	2,677	0,000	formimidoyltransferase-cyclodeaminase-like	PREDICTED: formimidoyltransferase-cyclodeaminase-like [Prunus mume]	Over Expressed	D48

s00087g07586	2,671	0,000	unnamed protein product	### Not characterized ###:unnamed protein product [Coffea canephora]	Over Expressed	D48
s00044g04765	2,671	0,001	-	***** No hits found ******	Over Expressed	D48
s00046g04956	2,668	0,000	pleiotropic drug resistance protein 1	Pleiotropic drug resistance protein 1 [Morus notabilis]	Over Expressed	D48
s00010g01651	2,654	0,005	serine/threonine-protein kinase nek3-like	PREDICTED: probable serine/threonine-protein kinase nek3-like [Citrus sinensis]	Over Expressed	D48
s10943g33294	2,616	0,000	dnaJ homolog subfamily B member 8-like	PREDICTED: dnaJ homolog subfamily B member 8-like [Pyrus x bretschneideri]	Over Expressed	D48
s00196g12215	2,614	0,038	-	***** No hits found ******	Over Expressed	D48
s00840g24420	2,607	0,040	-	***** No hits found ******	Over Expressed	D48
s00983g25879	2,586	0,000	-	### Not characterized ###:hypothetical protein L484_004442 [Morus notabilis]	Over Expressed	D48
s00496g19604	2,554	0,036	bidirectional sugar transporter SWEET16	Bidirectional sugar transporter SWEET16 [Morus notabilis]	Over Expressed	D48
s00456g18824	2,538	0,017	-	### Not characterized ###:hypothetical protein L484_002641 [Morus notabilis]	Over Expressed	D48
s00001g00053	2,530	0,017	-	PREDICTED: uncharacterized mitochondrial protein AtMg00810-like [Glycine max]	Over Expressed	D48
s00124g09364	2,530	0,015	E3 ubiquitin-protein ligase listerin	E3 ubiquitin-protein ligase listerin [Morus notabilis]	Over Expressed	D48
s00045g04880	2,523	0,000	serine/threonine-protein kinase SMG1	Serine/threonine-protein kinase SMG1 [Morus notabilis]	Over Expressed	D48
s00032g03821	2,502	0,000	glucanase like protein	Glucanase like protein [Theobroma cacao]	Over Expressed	D48
s01232g27721	2,491	0,004	-	PREDICTED: protein ZINC INDUCED FACILITATOR-LIKE 1-like isoform X2 [Malus domestica]	Over Expressed	D48
s00256g14057	2,480	0,001	-	PREDICTED: protein TRANSPARENT TESTA 12-like [Pyrus x bretschneideri]	Over Expressed	D48
s00814g24153	2,470	0,000	sugar transporter ERD6-like 6	Sugar transporter ERD6-like 6 [Morus notabilis]	Over Expressed	D48
s00587g21137	2,470	0,000	-	PREDICTED: putative disease resistance protein RGA3 [Jatropha curcas]	Over Expressed	D48
s11955g33520	2,465	0,003	-	cinnamate beta-D-glucosyltransferase-like [Vitis vinifera]	Over Expressed	D48
s00599g21349	2,460	0,008	-	### Not characterized ###:hypothetical protein L484_011011 [Morus notabilis]	Over Expressed	D48
s00006g01064	2,458	0,001	raffinose synthase family protein	Raffinose synthase family protein [Theobroma cacao]	Over Expressed	D48

s15417g34205	2,452	0,007	-	PREDICTED: protein GLUTAMINE DUMPER 6-like [Malus domestica]	Over Expressed	D48
s00294g15096	2,444	0,008	tetraspanin-19-like isoform X2	PREDICTED: tetraspanin-19-like isoform X2 [Prunus mume]	Over Expressed	D48
s00187g11925	2,435	0,000	alanine-glyoxylate aminotransferase	alanine-glyoxylate aminotransferase, putative [Ricinus communis]	Over Expressed	D48
s00068g06468	2,434	0,037	similar to L484_019165	### Not characterized ###:hypothetical protein L484_019165 [Morus notabilis]	Over Expressed	D48
s24576g35699	2,433	0,043	-	### Not characterized ###:PREDICTED: uncharacterized protein LOC105852868 [Cicer arietinum]	Over Expressed	D48
s00786g23838	2,414	0,000	subtilisin-like protease	Subtilisin-like protease [Morus notabilis]	Over Expressed	D48
s02848g31082	2,413	0,000	-	***** No hits found ******	Over Expressed	D48
s00759g23503	2,402	0,000	-	PREDICTED: protein YLS9 [Gossypium raimondii]	Over Expressed	D48
s00405g17768	2,400	0,007	-	### Not characterized ###:hypothetical protein L484_020052 [Morus notabilis]	Over Expressed	D48
s00041g04555	2,391	0,001	pre-mRNA cleavage complex 2 protein Pcf11, putative isoform 2	Pre-mRNA cleavage complex 2 protein Pcf11, putative isoform 2 [Theobroma cacao] gi|508779462|gb|EOY26718.1|	Over Expressed	D48
s00405g17769	2,387	0,008	-	***** No hits found ******	Over Expressed	D48
s00124g09408	2,371	0,024	uncharacterized protein LOC103329980	### Not characterized ###:PREDICTED: uncharacterized protein LOC103329980 [Prunus mume]	Over Expressed	D48
s00637g21925	2,360	0,000	-	PREDICTED: U4/U6.U5 tri-snRNP-associated protein 1-like [Malus domestica] gi|657997037|ref|XP_008390896.1|	Over Expressed	D48
s00404g17739	2,353	0,007	inactive leucine-rich repeat receptor-like protein kinase	putative inactive leucine-rich repeat receptor-like protein kinase [Morus notabilis] gi|587909790|gb|EXB97691.1|	Over Expressed	D48
s00418g18055	2,336	0,001	S-type anion channel SLAH3	S-type anion channel SLAH3 [Morus notabilis]	Over Expressed	D48
s00571g20919	2,331	0,004	atrophin-1	PREDICTED: LOW QUALITY PROTEIN: atrophin-1 [Cucumis melo]	Over Expressed	D48
s11929g33509	2,305	0,000	-	PREDICTED: protein ECERIFERUM 1-like, partial [Prunus mume]	Over Expressed	D48
s00320g15804	2,297	0,000	glycosyltransferase-like At2g41451	PREDICTED: glycosyltransferase-like At2g41451 [Nelumbo nucifera]	Over Expressed	D48
s00322g15855	2,290	0,010	-	### Not characterized ###:hypothetical protein L484_003435 [Morus notabilis]	Over Expressed	D48
s00154g10671	2,288	0,002	pentatricopeptide repeat-containing protein At3g47840	PREDICTED: putative pentatricopeptide repeat-containing protein At3g47840 [Pyrus x bretschneideri]	Over Expressed	D48
s13183g33771	2,271	0,000	-	PREDICTED: probable cytokinin riboside 5'-monophosphate phosphoribohydrolase LOGL1-like isoform X2 [Citrus sinensis]	Over Expressed	D48

s25457g35803	2,260	0,039	similar to L484_005846	### Not characterized ###:hypothetical protein L484_005846 [Morus notabilis]	Over Expressed	D48
s02187g30660	2,230	0,002	uncharacterized protein LOC102631362	### Not characterized ###:PREDICTED: uncharacterized protein LOC102631362 [Citrus sinensis]	Over Expressed	D48
s18780g34821	2,186	0,004	glucan endo-1,3-beta-glucosidase isoform X1	PREDICTED: glucan endo-1,3-beta-glucosidase isoform X1 [Jatropha curcas]	Over Expressed	D48
s05386g31855	2,176	0,000	-	PREDICTED: probable cytokinin riboside 5'-monophosphate phosphoribohydrolase LOGL1 [Vitis vinifera]	Over Expressed	D48
s00590g21184	2,175	0,003	similar to VITISV_024814	### Not characterized ###:hypothetical protein VITISV_024814 [Vitis vinifera]	Over Expressed	D48
s00478g19250	2,150	0,050	gibberellin 2-beta-dioxygenase 1	Gibberellin 2-beta-dioxygenase 1 [Morus notabilis]	Over Expressed	D48
s10712g33237	2,137	0,012	-	***** No hits found ******	Over Expressed	D48
s02091g30514	2,133	0,000	-	PREDICTED: cytokinin riboside 5'-monophosphate phosphoribohydrolase LOG8-like [Prunus mume]	Over Expressed	D48
s00165g11095	2,132	0,008	-	### Not characterized ###:hypothetical protein L484_012154 [Morus notabilis]	Over Expressed	D48
s00030g03632	2,123	0,036	alpha-aminoadipic semialdehyde synthase	Alpha-aminoadipic semialdehyde synthase [Morus notabilis]	Over Expressed	D48
s00308g15457	2,120	0,000	-	***** No hits found ******	Over Expressed	D48
s00199g12310	2,110	0,000	mitogen-activated protein kinase kinase kinase 2	Mitogen-activated protein kinase kinase kinase 2 [Morus notabilis]	Over Expressed	D48
s07887g32536	2,098	0,000	uncharacterized protein LOC104877618	### Not characterized ###:PREDICTED: uncharacterized protein LOC104877618 [Vitis vinifera]	Over Expressed	D48
s00287g14905	2,091	0,000	-	***** No hits found ******	Over Expressed	D48
s10042g33077	2,089	0,037	cellulose synthase-like protein G2	Cellulose synthase-like protein G2 [Morus notabilis]	Over Expressed	D48
s00006g01145	2,077	0,001	-	### Not characterized ###:hypothetical protein PRUPE_ppa018289mg [Prunus persica]	Over Expressed	D48
s00681g22514	2,074	0,015	cytochrome P450 78A4	Cytochrome P450 78A4 [Morus notabilis] gi|587838212|gb|EXB28925.1|	Over Expressed	D48
s00022g02925	2,062	0,021	polygalacturonase	Polygalacturonase [Morus notabilis] gi|587904952|gb|EXB93148.1|	Over Expressed	D48
s00287g14906	2,054	0,000	RING fingerand CHY zinc finger domain-containing protein 1-like	PREDICTED: RING fingerand CHY zinc finger domain-containing protein 1-like [Eucalyptus grandis]	Over Expressed	D48
s00247g13804	2,048	0,014	E3 ubiquitin-protein ligase RHA2A	E3 ubiquitin-protein ligase RHA2A [Morus notabilis]	Over Expressed	D48
s00305g15382	2,014	0,008	similar to L484_010847	### Not characterized ###:hypothetical protein L484_010847 [Morus notabilis]	Over Expressed	D48

s00083g07387	2,003	0,000	UDP-glycosyltransferase 73C3	UDP-glycosyltransferase 73C3 [Morus notabilis]	Over Expressed	D48
s00557g20673	2,000	0,022	-	PREDICTED: protein ECERIFERUM 1-like [Prunus mume]	Over Expressed	D48
s00120g09218	1,997	0,043	-	### Not characterized ###:hypothetical protein PRUPE_ppa016191mg [Prunus persica]	Over Expressed	D48
s00444g18584	1,987	0,000	similar to L484_000375	### Not characterized ###:hypothetical protein L484_000375 [Morus notabilis]	Over Expressed	D48
s00021g02872	1,985	0,020	thromboxane-A synthase	Thromboxane-A synthase [Morus notabilis]	Over Expressed	D48
s01069g26609	1,978	0,001	4-alpha-glucanotransferase DPE2	4-alpha-glucanotransferase DPE2 [Morus notabilis]	Over Expressed	D48
s00628g21805	1,961	0,008	similar to L484_006153	### Not characterized ###:hypothetical protein L484_006153 [Morus notabilis]	Over Expressed	D48
s21629g35283	1,959	0,021	-	### Not characterized ###:hypothetical protein VITISV_041073 [Vitis vinifera]	Over Expressed	D48
s00331g16057	1,953	0,000	serine/threonine-protein kinase SAPK3 isoform 2	Serine/threonine-protein kinase SAPK3 isoform 2, partial [Theobroma cacao] gi|508776903|gb|EOY24159.1|	Over Expressed	D48
s00287g14907	1,949	0,000	-	***** No hits found ******	Over Expressed	D48
s00749g23397	1,946	0,000	cytochrome P450	Cytochrome P450 [Morus notabilis] gi|587886359|gb|EXB75164.1|	Over Expressed	D48
s00611g21551	1,944	0,000	-	***** No hits found ******	Over Expressed	D48
s00614g21603	1,943	0,000	-	PREDICTED: protein ALTERED XYLOGLUCAN 4-like [Prunus mume]	Over Expressed	D48
s00187g11939	1,942	0,000	NAC domain-containing protein 72	NAC domain-containing protein 72 [Morus notabilis]	Over Expressed	D48
s08382g32673	1,938	0,034	-	***** No hits found ******	Over Expressed	D48
s00030g03631	1,930	0,036	lysine-ketoglutarate reductase/saccharopine dehydrogenase bifunctional enzyme	Lysine-ketoglutarate reductase/saccharopine dehydrogenase bifunctional enzyme [Theobroma cacao]	Over Expressed	D48
s00479g19278	1,930	0,000	purple acid phosphatase 3	Purple acid phosphatase 3 [Morus notabilis]	Over Expressed	D48
s00136g09906	1,919	0,015	two-component response regulator-like protein	Two-component response regulator-like protein [Morus notabilis]	Over Expressed	D48
s00046g04905	1,910	0,033	-	***** No hits found ******	Over Expressed	D48
s00151g10569	1,908	0,006	purple acid phosphatase 25	Purple acid phosphatase 25 [Morus notabilis]	Over Expressed	D48
s00207g12577	1,908	0,031	myb-related protein 305	Myb-related protein 305 [Morus notabilis]	Over Expressed	D48
s00232g13400	1,902	0,000	-	***** No hits found ******	Over Expressed	D48
s00101g08271	1,884	0,004	glycerol-3-phosphate transporter 4	Putative glycerol-3-phosphate transporter 4 [Morus notabilis]	Over Expressed	D48

s00261g14202	1,884	0,020	acyl-protein thioesterase 2-like	PREDICTED: acyl-protein thioesterase 2-like [Populus euphratica]	Over Expressed	D48
s00194g12157	1,871	0,007	-	PREDICTED: protein GLUTAMINE DUMPER 3-like [Malus domestica]	Over Expressed	D48
s00027g03337	1,869	0,044	similar to L484_015100	### Not characterized ###:hypothetical protein L484_015100 [Morus notabilis]	Over Expressed	D48
s00017g02349	1,860	0,000	tropinone reductase-like protein	Tropinone reductase-like protein [Morus notabilis]	Over Expressed	D48
s01327g28237	1,852	0,000	-	PREDICTED: proteasome activator subunit 4 [Solanum lycopersicum]	Over Expressed	D48
s01327g28234	1,848	0,000	amino acid transporter	amino acid transporter, putative [Ricinus communis]	Over Expressed	D48
s01825g30051	1,843	0,000	acetyltransferase	putative acetyltransferase [Morus notabilis]	Over Expressed	D48
s00067g06403	1,831	0,007	-	### Not characterized ###:hypothetical protein L484_027125 [Morus notabilis]	Over Expressed	D48
s00679g22491	1,828	0,000	nuclear transcription factor Y subunit A-9	Nuclear transcription factor Y subunit A-9 [Morus notabilis]	Over Expressed	D48
s00471g19142	1,826	0,000	-	### Not characterized ###:hypothetical protein B456_004G198900 [Gossypium raimondii]	Over Expressed	D48
s00004g00666	1,820	0,000	-	***** No hits found ******	Over Expressed	D48
s00384g17305	1,819	0,000	abscisic acid-deficient 4	Abscisic acid-deficient 4 [Theobroma cacao]	Over Expressed	D48
s02020g30426	1,817	0,000	-	putative Ty1-copia-like retrotransposon [Cercis chinensis]	Over Expressed	D48
s00020g02736	1,810	0,047	serine/threonine-protein kinase WNK-related isoform 1	Serine/threonine-protein kinase WNK-related isoform 1 [Theobroma cacao] gi|508722478|gb|EOY14375.1|	Over Expressed	D48
s00820g24228	1,801	0,026	ripening-related protein-like	ripening-related protein-like [Vitis vinifera]	Over Expressed	D48
s00821g24240	1,796	0,000	inositol oxygenase 1-like isoform X1	PREDICTED: inositol oxygenase 1-like isoform X1 [Citrus sinensis]	Over Expressed	D48
s00008g01339	1,792	0,029	-	***** No hits found ******	Over Expressed	D48
s00112g08814	1,781	0,001	lipoamide acyltransferase componentof branched-chain alpha-keto acid dehydrogenase complex	Lipoamide acyltransferase componentof branched-chain alpha-keto acid dehydrogenase complex [Morus notabilis]	Over Expressed	D48
s00125g09432	1,779	0,002	esterase-like	PREDICTED: LOW QUALITY PROTEIN: esterase-like [Prunus mume]	Over Expressed	D48
s00043g04721	1,779	0,001	-	***** No hits found ******	Over Expressed	D48
s00017g02409	1,771	0,000	-	Glucan endo-1,3-beta-glucosidase [Morus notabilis]	Over Expressed	D48
s00103g08372	1,768	0,000	glutathione S-transferase	PREDICTED: probable glutathione S-transferase [Prunus mume]	Over Expressed	D48

s00048g05051	1,768	0,020	U-box domain-containing protein 20	U-box domain-containing protein 20 [Morus notabilis]	Over Expressed	D48
s00789g23879	1,766	0,000	similar to L484_003112	### Not characterized ###:hypothetical protein L484_003112 [Morus notabilis]	Over Expressed	D48
s00595g21295	1,764	0,000	monosaccharide-sensing protein 2	Monosaccharide-sensing protein 2 [Morus notabilis]	Over Expressed	D48
s00156g10751	1,762	0,000	syntaxin-binding protein 5	Syntaxin-binding protein 5 [Morus notabilis]	Over Expressed	D48
s00067g06394	1,760	0,000	serine/threonine-protein kinase	Serine/threonine-protein kinase [Morus notabilis]	Over Expressed	D48
s00368g16954	1,759	0,012	translocator-like protein	Translocator-like protein [Morus notabilis]	Over Expressed	D48
s00099g08222	1,757	0,011	interaptin	PREDICTED: interaptin [Prunus mume]	Over Expressed	D48
s01339g28292	1,756	0,012	-	***** No hits found ******	Over Expressed	D48
s00258g14107	1,755	0,001	-	### Not characterized ###:hypothetical protein L484_002671 [Morus notabilis]	Over Expressed	D48
s06049g32019	1,752	0,040	uncharacterized protein LOC104879739	### Not characterized ###:PREDICTED: uncharacterized protein LOC104879739 [Vitis vinifera]	Over Expressed	D48
s00786g23837	1,749	0,001	-	protein phosphatase 2c, putative [Ricinus communis]	Over Expressed	D48
s00168g11211	1,733	0,026	thiamine thiazole synthase 2	Thiamine thiazole synthase 2 [Morus notabilis]	Over Expressed	D48
s00187g11938	1,733	0,005	-	***** No hits found ******	Over Expressed	D48
s00423g18151	1,732	0,000	similar to L484_022081	### Not characterized ###:hypothetical protein L484_022081 [Morus notabilis]	Over Expressed	D48
s00203g12451	1,720	0,033	sulfate transporter 4.2	putative sulfate transporter 4.2 [Morus notabilis]	Over Expressed	D48
s00038g04301	1,709	0,000	leucine-rich repeat receptor protein kinase EXS	Leucine-rich repeat receptor protein kinase EXS [Morus notabilis]	Over Expressed	D48
s00077g06986	1,698	0,023	adenine/guanine permease AZG1	Adenine/guanine permease AZG1 [Morus notabilis]	Over Expressed	D48
s00061g06023	1,689	0,003	copper chaperonefor superoxide dismutase	Copper chaperonefor superoxide dismutase [Morus notabilis]	Over Expressed	D48
s00199g12309	1,685	0,004	aspartic proteinase	Aspartic proteinase [Morus notabilis] gi|587888347|gb|EXB77055.1|	Over Expressed	D48
s24475g35680	1,684	0,001	retrovirus-related Pol polyprotein	Retrovirus-related Pol polyprotein from transposon TNT 1-94 [Morus notabilis] gi|587949157|gb|EXC35359.1|	Over Expressed	D48
s00004g00768	1,682	0,006	glucose-1-phosphate adenylyltransferase large subunit	Glucose-1-phosphate adenylyltransferase large subunit [Morus notabilis] gi|587938319|gb|EXC25068.1|	Over Expressed	D48
s01118g26941	1,675	0,012	BTB/POZ domain-containing protein	BTB/POZ domain-containing protein [Morus notabilis]	Over Expressed	D48

s03669g31340	1,671	0,000	-	PREDICTED: hippocampus abundant transcript-like protein 1 isoform X2 [Sesamum indicum] gi|747060950|ref|XP_011076926.1|	Over Expressed	D48
s00043g04722	1,670	0,000	-	NHX1 [Morus notabilis]	Over Expressed	D48
s00080g07237	1,667	0,001	myosin heavy chain kinase B	Myosin heavy chain kinase B [Morus notabilis]	Over Expressed	D48
s00152g10615	1,656	0,000	serine/threonine-protein kinase Nek7	Serine/threonine-protein kinase Nek7 [Morus notabilis]	Over Expressed	D48
s00903g25127	1,649	0,012	glycerol-3-phosphate acyltransferase	glycerol-3-phosphate acyltransferase [Medicago truncatula]	Over Expressed	D48
s00181g11655	1,649	0,031	-	### Not characterized ###:hypothetical protein L484_010593 [Morus notabilis]	Over Expressed	D48
s00017g02350	1,641	0,005	-	Tropinone reductase-like protein [Morus notabilis]	Over Expressed	D48
s00291g15026	1,639	0,040	cytochrome P450 71D11-like	PREDICTED: cytochrome P450 71D11-like [Fragaria vesca subsp. vesca]	Over Expressed	D48
s00308g15456	1,638	0,001	folate-biopterin transporter 7-like	PREDICTED: probable folate-biopterin transporter 7-like [Citrus sinensis]	Over Expressed	D48
s06168g32051	1,637	0,000	-	***** No hits found ******	Over Expressed	D48
s00982g25867	1,635	0,000	-	***** No hits found ******	Over Expressed	D48
s11820g33480	1,631	0,015	-	### Not characterized ###:hypothetical protein POPTR_0001s42340g, partial [Populus trichocarpa] gi|550349590|gb|ERP66980.1|	Over Expressed	D48
s00038g04289	1,630	0,025	serine/threonine-protein kinase	putative serine/threonine-protein kinase [Morus notabilis]	Over Expressed	D48
s01290g28025	1,628	0,019	similar to L484_016377	### Not characterized ###:hypothetical protein L484_016377 [Morus notabilis]	Over Expressed	D48
s00423g18156	1,627	0,000	alpha-glucan phosphorylase, H isozyme	Alpha-glucan phosphorylase, H isozyme [Morus notabilis]	Over Expressed	D48
s00430g18286	1,626	0,002	selenium-binding protein 2	Selenium-binding protein 2 [Morus notabilis]	Over Expressed	D48
s06985g32279	1,626	0,035	acetyl-CoA carboxylase 1	Acetyl-CoA carboxylase 1 [Morus notabilis]	Over Expressed	D48
s00094g07955	1,626	0,029	copper-transporting ATPase 3	Putative copper-transporting ATPase 3 [Morus notabilis]	Over Expressed	D48
s01205g27550	1,624	0,004	similar to L484_009885	### Not characterized ###:hypothetical protein L484_009885 [Morus notabilis]	Over Expressed	D48
s00138g10001	1,620	0,000	galacturonosyltransferase-like 9	putative galacturonosyltransferase-like 9 [Morus notabilis]	Over Expressed	D48
s00746g23361	1,618	0,000	myb domain protein 17 isoform 1	Myb domain protein 17 isoform 1 [Theobroma cacao]	Over Expressed	D48
s00061g05989	1,611	0,042	DNA-directed RNA polymerase D subunit 1	DNA-directed RNA polymerase D subunit 1 [Morus notabilis]	Over Expressed	D48
s00017g02427	1,610	0,000	E3 ubiquitin-protein ligase XBAT31	Putative E3 ubiquitin-protein ligase XBAT31 [Morus notabilis]	Over Expressed	D48
s11486g33417	1,605	0,026	disease resistance protein At3g14460	PREDICTED: putative disease resistance protein At3g14460 [Pyrus x bretschneideri] gi|694392078|ref|XP_009371531.1|	Over Expressed	D48

s00001g00008	1,602	0,007	-	***** No hits found ******	Over Expressed	D48
s00319g15780	1,599	0,001	alpha-1,4 glucan phosphorylase L isozyme	Alpha-1,4 glucan phosphorylase L isozyme [Morus notabilis]	Over Expressed	D48
s00118g09118	1,598	0,000	flavonoid 3-O-glucosyltransferase	flavonoid 3-O-glucosyltransferase, partial [Morus alba]	Over Expressed	D48
s01118g26940	1,594	0,020	BTB/POZ domain-containing protein	BTB/POZ domain-containing protein [Morus notabilis]	Over Expressed	D48
s00212g12760	1,590	0,000	phosphoenolpyruvate carboxylase	Phosphoenolpyruvate carboxylase [Morus notabilis]	Over Expressed	D48
s00022g02966	1,587	0,008	patatin group A-3	Patatin group A-3 [Morus notabilis] gi|587904995|gb|EXB93191.1|	Over Expressed	D48
s01401g28611	1,580	0,002	1-phosphatidylinositol phosphodiesterase	1-phosphatidylinositol phosphodiesterase [Morus notabilis]	Over Expressed	D48
s02837g31079	1,574	0,001	-	Selenium-binding protein 2 [Morus notabilis]	Over Expressed	D48
s00041g04572	1,574	0,000	mitochondrial carnitine/acylcarnitine carrier protein CACL	Mitochondrial carnitine/acylcarnitine carrier protein CACL [Morus notabilis] gi|587885639|gb|EXB74496.1|	Over Expressed	D48
s00057g05768	1,569	0,000	uncharacterized membrane protein	Uncharacterized membrane protein [Morus notabilis]	Over Expressed	D48
s00015g02137	1,551	0,037	MATE efflux family protein DTX1	MATE efflux family protein DTX1 [Morus notabilis]	Over Expressed	D48
s02687g31017	1,550	0,001	allantoate deiminase	Allantoate deiminase [Morus notabilis] gi|587877959|gb|EXB66978.1|	Over Expressed	D48
s00124g09363	1,542	0,038	uncharacterized protein LOC102629445	### Not characterized ###:PREDICTED: uncharacterized protein LOC102629445 [Citrus sinensis]	Over Expressed	D48
s00007g01172	1,541	0,000	serine/threonine-protein kinase SRK2E	Serine/threonine-protein kinase SRK2E [Theobroma cacao]	Over Expressed	D48
s00312g15567	1,530	0,000	1-aminocyclopropane-1-carboxylate oxidase homolog 1-like	PREDICTED: 1-aminocyclopropane-1-carboxylate oxidase homolog 1-like [Malus domestica]	Over Expressed	D48
s00037g04203	1,530	0,000	formin-like protein 6	Formin-like protein 6 [Morus notabilis] gi|587905947|gb|EXB94058.1|	Over Expressed	D48
s07023g32286	1,529	0,007	retrovirus-related Pol polyprotein	Retrovirus-related Pol polyprotein from transposon TNT 1-94 [Morus notabilis] gi|587900079|gb|EXB88426.1|	Over Expressed	D48
s12812g33704	1,524	0,001	-	***** No hits found ******	Over Expressed	D48
s00435g18390	1,511	0,039	FAD-binding Berberine family protein	FAD-binding Berberine family protein [Theobroma cacao]	Over Expressed	D48
s00065g06281	1,505	0,000	uncharacterized protein LOC103449078	### Not characterized ###:PREDICTED: uncharacterized protein LOC103449078 [Malus domestica]	Over Expressed	D48
s00397g17581	1,500	0,000	-	### Not characterized ###:hypothetical protein L484_013187 [Morus notabilis]	Over Expressed	D48
s00269g14436	1,499	0,049	cyclic nucleotide-gated ionchannel 2-like	PREDICTED: cyclic nucleotide-gated ionchannel 2-like [Citrus sinensis]	Over Expressed	D48

s01649g29575	1,496	0,024	similar to VITISV_002211	### Not characterized ###:hypothetical protein VITISV_002211 [Vitis vinifera]	Over Expressed	D48
s00080g07234	1,496	0,001	kinase superfamily protein	Kinase superfamily protein [Theobroma cacao]	Over Expressed	D48
s00114g08941	1,486	0,000	DNA ligase	DNA ligase [Gossypium arboreum]	Over Expressed	D48
s00017g02428	1,486	0,040	-	***** No hits found ******	Over Expressed	D48
s00026g03291	1,482	0,016	NAC domain-containing protein 29	NAC domain-containing protein 29 [Morus notabilis]	Over Expressed	D48
s10171g33112	1,482	0,044	cellulose synthase-like protein G2	Cellulose synthase-like protein G2 [Morus notabilis]	Over Expressed	D48
s00010g01565	1,482	0,045	-	***** No hits found ******	Over Expressed	D48
s01445g28803	1,468	0,000	homeobox protein 31	Homeobox protein 31 [Theobroma cacao] gi|508778518|gb|EOY25774.1|	Over Expressed	D48
s00358g16681	1,467	0,019	-	orf186 (mitochondrion) [Phoenix dactylifera]	Over Expressed	D48
s00172g11380	1,465	0,031	NAC domain-containing protein 29	NAC domain-containing protein 29 [Morus notabilis]	Over Expressed	D48
s00014g02018	1,461	0,011	-	PREDICTED: ABC transporter C family member 8-like [Prunus mume]	Over Expressed	D48
s00004g00672	1,456	0,001	auxin response factor 5	Auxin response factor 5 [Morus notabilis]	Over Expressed	D48
s00077g07008	1,449	0,001	transcription factor RF2b	Transcription factor RF2b [Morus notabilis]	Over Expressed	D48
s01861g30132	1,441	0,004	LRR receptor-like serine/threonine-protein kinase At1g07650 isoform X1	PREDICTED: probable LRR receptor-like serine/threonine-protein kinase At1g07650 isoform X1 [Prunus mume]	Over Expressed	D48
s00526g20156	1,440	0,000	common plant regulatory factor 1	Common plant regulatory factor 1 [Morus notabilis]	Over Expressed	D48
s00099g08223	1,439	0,023	similar to L484_015783	### Not characterized ###:hypothetical protein L484_015783 [Morus notabilis]	Over Expressed	D48
s13942g33922	1,430	0,045	-	***** No hits found ******	Over Expressed	D48
s00481g19303	1,412	0,019	copperion binding protein	Copper ion binding protein, putative [Ricinus communis]	Over Expressed	D48
s00780g23762	1,408	0,016	heat stress transcription factor A-2	Heat stress transcription factor A-2 [Morus notabilis]	Over Expressed	D48
s00272g14499	1,401	0,036	-	PREDICTED: defensin Ec-AMP-D2-like [Nelumbo nucifera]	Over Expressed	D48
s00237g13551	1,401	0,001	two-component response regulator-like protein	Two-component response regulator-like protein [Morus notabilis]	Over Expressed	D48
s00581g21077	1,400	0,040	-	### Not characterized ###:hypothetical protein PRUPE_ppa015045mg [Prunus persica]	Over Expressed	D48
s00013g01956	1,399	0,000	similar to L484_010133	### Not characterized ###:hypothetical protein L484_010133 [Morus notabilis]	Over Expressed	D48
s01210g27592	1,398	0,037	C2 and GRAM domain-containing protein	C2 and GRAM domain-containing protein [Morus notabilis]	Over Expressed	D48

s00054g05527	1,396	0,008	alpha,alpha-trehalose-phosphate synthase [UDP-forming] 5	Alpha,alpha-trehalose-phosphate synthase [UDP-forming] 5 [Morus notabilis] gi|587877496|gb|EXB66534.1|	Over Expressed	D48
s00022g02901	1,395	0,000	UDP-N-acetylglucosamine--peptide N-acetylglucosaminyltransferase SEC	putative UDP-N-acetylglucosamine--peptide N-acetylglucosaminyltransferase SEC [Morus notabilis]	Over Expressed	D48
s00003g00522	1,393	0,043	serine carboxypeptidase-like 48	Serine carboxypeptidase-like 48 [Morus notabilis]	Over Expressed	D48
s00115g08974	1,388	0,003	seed maturation protein LEA 4	seed maturation protein LEA 4 [Glycine tomentella]	Over Expressed	D48
s00021g02805	1,385	0,000	branched-chain-amino-acid aminotransferase 2	Branched-chain-amino-acid aminotransferase 2 [Morus notabilis]	Over Expressed	D48
s00129g09594	1,382	0,005	-	***** No hits found ******	Over Expressed	D48
s01329g28248	1,380	0,001	histidine decarboxylase	Histidine decarboxylase [Morus notabilis]	Over Expressed	D48
s00180g11622	1,379	0,005	similar to L484_024270	### Not characterized ###:hypothetical protein L484_024270 [Morus notabilis]	Over Expressed	D48
s00003g00546	1,375	0,004	P-loop containing nucleoside triphosphate hydrolases superfamily protein isoform 1	P-loop containing nucleoside triphosphate hydrolases superfamily protein isoform 1 [Theobroma cacao]	Over Expressed	D48
s00035g04065	1,373	0,026	ATP binding protein	ATP binding protein, putative [Ricinus communis]	Over Expressed	D48
s00057g05767	1,368	0,012	proline-rich receptor-like protein kinase PERK8	Proline-rich receptor-like protein kinase PERK8 [Morus notabilis]	Over Expressed	D48
s00147g10413	1,366	0,003	granule-bound starch synthase 1	Granule-bound starch synthase 1 [Morus notabilis]	Over Expressed	D48
s20056g35047	1,358	0,000	receptor-like serine/threonine-protein kinase	putative receptor-like serine/threonine-protein kinase [Morus notabilis] gi|587930118|gb|EXC17247.1|	Over Expressed	D48
s12175g33569	1,353	0,000	magnesium transporter NIPA8	PREDICTED: probable magnesium transporter NIPA8 [Fragaria vesca subsp. vesca]	Over Expressed	D48
s00044g04762	1,350	0,032	xyloglucanase inhibitor 3	xyloglucanase inhibitor 3 [Humulus lupulus]	Over Expressed	D48
s00007g01232	1,349	0,004	O-fucosyltransferase family protein isoform 1	O-fucosyltransferase family protein isoform 1 [Theobroma cacao]	Over Expressed	D48
s00120g09232	1,348	0,014	membrane-associated kinase regulator 1	PREDICTED: probable membrane-associated kinase regulator 1 [Populus euphratica]	Over Expressed	D48
s00282g14764	1,346	0,041	-	PREDICTED: dentin sialophosphoprotein [Malus domestica]	Over Expressed	D48
s00151g10558	1,341	0,000	receptor-like protein 12	Receptor-like protein 12 [Morus notabilis]	Over Expressed	D48
s00100g08254	1,340	0,000	phosphoglucomutase / glucose phosphomutase	Phosphoglucomutase / glucose phosphomutase, putative [Theobroma cacao] gi|508709124|gb|EOY01021.1|	Over Expressed	D48
s00022g02952	1,331	0,028	phosphoenolpyruvate carboxylase kinase 1	Phosphoenolpyruvate carboxylase kinase 1 [Morus notabilis]	Over Expressed	D48
s00913g25228	1,331	0,031	9-cis-epoxycarotenoid dioxygenase	9-cis-epoxycarotenoid dioxygenase [Morus alba var. multicaulis]	Over Expressed	D48
s00622g21724	1,330	0,013	F-box/FBD/LRR-repeat protein	F-box/FBD/LRR-repeat protein [Morus notabilis]	Over Expressed	D48

s00038g04309	1,329	0,000	enolase	PREDICTED: enolase [Pyrus x bretschneideri]	Over Expressed	D48
s00089g07654	1,327	0,000	heavy metal-associated isoprenylated plant protein 26-like	PREDICTED: heavy metal-associated isoprenylated plant protein 26-like [Populus euphratica]	Over Expressed	D48
s16614g34429	1,325	0,000	E3 ubiquitin-protein ligase RHA2B	putative E3 ubiquitin-protein ligase RHA2B [Morus notabilis]	Over Expressed	D48
s00003g00517	1,324	0,049	-	PREDICTED: protein NUCLEAR FUSION DEFECTIVE 4-like isoform X1 [Fragaria vesca subsp. vesca]	Over Expressed	D48
s00140g10067	1,306	0,021	lupeol synthase	Lupeol synthase [Morus notabilis] gi|587869349|gb|EXB58667.1|	Over Expressed	D48
s00176g11521	1,305	0,037	-	### Not characterized ###:PREDICTED: uncharacterized protein LOC104879574 [Vitis vinifera]	Over Expressed	D48
s00244g13724	1,300	0,000	uncharacterized protein L484_006539	### Not characterized ###:Uncharacterized protein L484_006539 [Morus notabilis]	Over Expressed	D48
s00863g24673	1,300	0,000	-	UPF0481 protein [Morus notabilis] gi|587872854|gb|EXB62071.1|	Over Expressed	D48
s00374g17086	1,296	0,033	pentatricopeptide repeat-containing protein At1g43980, mitochondrial-like	PREDICTED: pentatricopeptide repeat-containing protein At1g43980, mitochondrial-like [Pyrus x bretschneideri]	Over Expressed	D48
s00123g09350	1,293	0,000	progesterone 5beta-reductase	putative progesterone 5beta-reductase [Ricinus communis]	Over Expressed	D48
s01180g27397	1,292	0,005	callose synthase 1	Callose synthase 1 [Morus notabilis] gi|587888635|gb|EXB77332.1|	Over Expressed	D48
s17273g34548	1,292	0,000	UDP-glycosyltransferase 85A1	UDP-glycosyltransferase 85A1 [Morus notabilis]	Over Expressed	D48
s00201g12386	1,290	0,001	-	***** No hits found ******	Over Expressed	D48
s04321g31545	1,290	0,007	-	***** No hits found ******	Over Expressed	D48
s00280g14730	1,284	0,023	similar to L484_024389	### Not characterized ###:hypothetical protein L484_024389 [Morus notabilis]	Over Expressed	D48
s00080g07240	1,284	0,003	cytochrome P450 82A3	Cytochrome P450 82A3 [Morus notabilis] gi|587919895|gb|EXC07349.1|	Over Expressed	D48
s01090g26759	1,277	0,000	J domain-containing protein requiredfor chloroplast accumulation response 1 isoform X1	PREDICTED: J domain-containing protein requiredfor chloroplast accumulation response 1 isoform X1 [Prunus mume]	Over Expressed	D48
s00133g09788	1,274	0,025	pheophytinase, chloroplastic	PREDICTED: pheophytinase, chloroplastic [Fragaria vesca subsp. vesca]	Over Expressed	D48
s00014g02014	1,260	0,039	ABC transporter C family member 8	ABC transporter C family member 8 [Morus notabilis]	Over Expressed	D48
s00350g16496	1,247	0,000	scarecrow-like protein 3	PREDICTED: scarecrow-like protein 3 [Jatropha curcas]	Over Expressed	D48
s00341g16287	1,246	0,020	nucleoredoxin 2	PREDICTED: probable nucleoredoxin 2 [Prunus mume]	Over Expressed	D48
s00034g03979	1,243	0,024	CBL-interacting serine/threonine-protein kinase 5	CBL-interacting serine/threonine-protein kinase 5 [Morus notabilis]	Over Expressed	D48
s00507g19814	1,242	0,035	sucrose synthase	Sucrose synthase [Morus notabilis] gi|587926354|gb|EXC13595.1|	Over Expressed	D48

s00022g02900	1,242	0,000	UDP-N-acetylglucosamine--peptide N-acetylglucosaminyltransferase SEC	putative UDP-N-acetylglucosamine--peptide N-acetylglucosaminyltransferase SEC [Morus notabilis]	Over Expressed	D48
s00001g00054	1,241	0,001	uncharacterized protein LOC102627405	### Not characterized ###:PREDICTED: uncharacterized protein LOC102627405 [Citrus sinensis]	Over Expressed	D48
s00281g14755	1,235	0,001	adagio protein 3	Adagio protein 3 [Morus notabilis] gi|587841729|gb|EXB32326.1|	Over Expressed	D48
s00062g06071	1,233	0,016	calcium-binding EF-hand family protein	Calcium-binding EF-hand family protein [Theobroma cacao]	Over Expressed	D48
s00128g09563	1,228	0,004	ABC transporter I family member 17	ABC transporter I family member 17 [Morus notabilis]	Over Expressed	D48
s00014g02078	1,225	0,013	-	PREDICTED: 60S ribosomal protein L18a-like protein [Pyrus x bretschneideri]	Over Expressed	D48
s12592g33664	1,222	0,023	-	***** No hits found ******	Over Expressed	D48
s00247g13789	1,216	0,010	ABC transporter G family member 14	ABC transporter G family member 14 [Morus notabilis]	Over Expressed	D48
s01169g27330	1,214	0,008	serine/threonine-protein kinase samkC	PREDICTED: probable serine/threonine-protein kinase samkC [Pyrus x bretschneideri]	Over Expressed	D48
s00653g22154	1,211	0,012	-	copia-like polyprotein [Arabidopsis thaliana]	Over Expressed	D48
s00009g01544	1,210	0,049	similar to L484_002709	### Not characterized ###:hypothetical protein L484_002709 [Morus notabilis]	Over Expressed	D48
s00363g16834	1,209	0,000	cysteine-rich receptor-like protein kinase 19 isoform X2	PREDICTED: cysteine-rich receptor-like protein kinase 19 isoform X2 [Prunus mume]	Over Expressed	D48
s00031g03740	1,204	0,043	formin-like protein 20	Formin-like protein 20 [Morus notabilis]	Over Expressed	D48
s00014g02016	1,202	0,030	ABC transporter C family member 8	ABC transporter C family member 8 [Morus notabilis]	Over Expressed	D48
s02148g30626	1,198	0,006	pyrophosphate-energized vacuolar membrane proton pump	Pyrophosphate-energized vacuolar membrane proton pump [Morus notabilis] gi|587877596|gb|EXB66631.1|	Over Expressed	D48
s00896g25044	1,194	0,000	ribonuclease J	Ribonuclease J [Morus notabilis] gi|587916461|gb|EXC04124.1|	Over Expressed	D48
s01268g27931	1,194	0,049	auxin-induced protein 5NG4	Auxin-induced protein 5NG4 [Morus notabilis]	Over Expressed	D48
s00062g06053	1,190	0,033	subtilisin-like protease	Subtilisin-like protease [Morus notabilis]	Over Expressed	D48
s00033g03917	1,186	0,002	aspartate, glycine, lysineand serine-rich protein-like	PREDICTED: aspartate, glycine, lysineand serine-rich protein-like [Prunus mume]	Over Expressed	D48
s00051g05309	1,184	0,020	-	PREDICTED: protein DA1-related 2-like [Pyrus x bretschneideri]	Over Expressed	D48
s20785g35159	1,173	0,046	-	***** No hits found ******	Over Expressed	D48
s00250g13895	1,167	0,044	-	PREDICTED: LOW QUALITY PROTEIN: protein TIME FOR COFFEE [Prunus mume]	Over Expressed	D48
s21819g35310	1,166	0,025	-	PREDICTED: putative disease resistance RPP13-like protein 1 [Prunus mume]	Over Expressed	D48

s00055g05607	1,166	0,001	1-aminocyclopropane-1-carboxylate oxidase 3	PREDICTED: 1-aminocyclopropane-1-carboxylate oxidase 3 [Prunus mume]	Over Expressed	D48
s00122g09306	1,165	0,000	-	***** No hits found ******	Over Expressed	D48
s00257g14096	1,163	0,000	niemann-Pick C1 protein	Niemann-Pick C1 protein [Morus notabilis] gi|587866873|gb|EXB56311.1|	Over Expressed	D48
s00042g04620	1,163	0,019	lysine-specific demethylase 8	Lysine-specific demethylase 8 [Morus notabilis]	Over Expressed	D48
s00293g15070	1,163	0,000	senescence-associated family protein	senescence-associated family protein [Populus trichocarpa]	Over Expressed	D48
s00011g01759	1,161	0,026	similar to L484_022107	### Not characterized ###:hypothetical protein L484_022107 [Morus notabilis]	Over Expressed	D48
s00458g18878	1,161	0,011	aquaporin TIP4-1	Aquaporin TIP4-1 [Morus notabilis] gi|587950043|gb|EXC36042.1|	Over Expressed	D48
s00004g00752	1,161	0,003	omega-hydroxypalmitate O-feruloyl transferase	Omega-hydroxypalmitate O-feruloyl transferase [Morus notabilis]	Over Expressed	D48
s00713g22920	1,159	0,037	pentatricopeptide repeat-containing protein At4g21065	PREDICTED: pentatricopeptide repeat-containing protein At4g21065 [Vitis vinifera]	Over Expressed	D48
s00097g08127	1,157	0,005	persulfide dioxygenase ETHE1 homolog, mitochondrial-like	PREDICTED: persulfide dioxygenase ETHE1 homolog, mitochondrial-like [Malus domestica]	Over Expressed	D48
s00127g09536	1,153	0,014	alpha-glucan water dikinase	Alpha-glucan water dikinase [Morus notabilis]	Over Expressed	D48
s00153g10646	1,151	0,030	B3 domain-containing transcription factor VRN1	B3 domain-containing transcription factor VRN1 [Morus notabilis]	Over Expressed	D48
s00858g24618	1,149	0,026	similar to VITISV_025708	### Not characterized ###:hypothetical protein VITISV_025708 [Vitis vinifera]	Over Expressed	D48
s00809g24104	1,149	0,032	hippocampus abundant transcript-like protein 1	Hippocampus abundant transcript-like protein 1 [Morus notabilis]	Over Expressed	D48
s00704g22801	1,147	0,026	gamma-irradiation and mitomycin c induced 1, putative isoform 1	Gamma-irradiation andmitomycinc induced 1, putative isoform 1 [Theobroma cacao] gi|508776308|gb|EOY23564.1|	Over Expressed	D48
s01975g30351	1,146	0,039	1-phosphatidylinositol-3-phosphate 5-kinase	1-phosphatidylinositol-3-phosphate 5-kinase [Morus notabilis]	Over Expressed	D48
s00433g18349	1,140	0,040	filament-like plant protein 7	PREDICTED: filament-like plant protein 7 [Prunus mume]	Over Expressed	D48
s00051g05290	1,136	0,001	sodium-dependent phosphate transport protein 1, chloroplastic isoform X1	PREDICTED: sodium-dependent phosphate transport protein 1, chloroplastic isoform X1 [Jatropha curcas]	Over Expressed	D48
s08065g32585	1,131	0,001	-	Transcription factor RAX2 [Morus notabilis]	Over Expressed	D48
s00616g21633	1,121	0,025	delta-1-pyrroline-5-carboxylate synthase	Delta-1-pyrroline-5-carboxylate synthase [Morus notabilis]	Over Expressed	D48
s00607g21475	1,117	0,027	-	Protein SENSITIVE TO PROTON RHIZOTOXICITY 1 [Morus notabilis]	Over Expressed	D48

s00223g13106	1,116	0,001	receptor-like serine/threonine-protein kinase	putative receptor-like serine/threonine-protein kinase [Morus notabilis] gi|587930118|gb|EXC17247.1|	Over Expressed	D48
s00101g08269	1,116	0,015	vesicle-associated protein 2-1	Vesicle-associated protein 2-1 [Morus notabilis]	Over Expressed	D48
s00248g13812	1,116	0,021	tubulin beta-1 chain	Tubulin beta-1 chain [Morus notabilis] gi|587839844|gb|EXB30492.1|	Over Expressed	D48
s00003g00556	1,113	0,009	arsenical pump-driving ATPase	Putative arsenical pump-driving ATPase [Morus notabilis]	Over Expressed	D48
s00067g06373	1,113	0,017	inactive leucine-rich repeat receptor-like protein kinase	putative inactive leucine-rich repeat receptor-like protein kinase [Morus notabilis] gi|587933566|gb|EXC20529.1|	Over Expressed	D48
s00016g02331	1,108	0,035	ninja-family protein AFP2-like	PREDICTED: ninja-family protein AFP2-like [Populus euphratica]	Over Expressed	D48
s01143g27133	1,108	0,000	similar to L484_003205	### Not characterized ###:hypothetical protein L484_003205 [Morus notabilis]	Over Expressed	D48
s00142g10132	1,104	0,000	U-box domain-containing protein 4	U-box domain-containing protein 4 [Morus notabilis]	Over Expressed	D48
s00541g20418	1,103	0,017	-	PREDICTED: protein CHUP1, chloroplastic [Prunus mume]	Over Expressed	D48
s00089g07695	1,103	0,000	hippocampus abundant transcript 1 protein	Hippocampus abundant transcript 1 protein [Morus notabilis]	Over Expressed	D48
s00020g02774	1,102	0,002	ABSCISIC ACID-INSENSITIVE 5-like protein 5	ABSCISIC ACID-INSENSITIVE 5-like protein 5 [Morus notabilis]	Over Expressed	D48
s00068g06454	1,101	0,000	GATA zinc finger domain-containing protein C1393.08 isoform 1	GATA zinc finger domain-containing protein C1393.08 isoform 1 [Theobroma cacao] gi|508716396|gb|EOY08293.1|	Over Expressed	D48
s00005g00935	1,099	0,006	similar to L484_008475	### Not characterized ###:hypothetical protein L484_008475 [Morus notabilis]	Over Expressed	D48
s00567g20831	1,097	0,006	mitochondrial chaperone bcs1	putative mitochondrial chaperone bcs1 [Morus notabilis]	Over Expressed	D48
s00126g09485	1,089	0,017	gibberellin receptor GID1B	Gibberellin receptor GID1B [Morus notabilis]	Over Expressed	D48
s00882g24871	1,089	0,016	UDP-glucose flavonoid 3-O-glucosyltransferase 6-like	PREDICTED: UDP-glucose flavonoid 3-O-glucosyltransferase 6-like [Prunus mume]	Over Expressed	D48
s00142g10154	1,084	0,041	transporter	putative transporter [Morus notabilis] gi|587907084|gb|EXB95111.1|	Over Expressed	D48
s00036g04142	1,084	0,041	similar to L484_005803	### Not characterized ###:hypothetical protein L484_005803 [Morus notabilis]	Over Expressed	D48

s00133g09761	1,083	0,049	E3 ubiquitin-protein ligase UPL1	E3 ubiquitin-protein ligase UPL1 [Morus notabilis]	Over Expressed	D48
s09585g32975	1,082	0,049	-	***** No hits found ******	Over Expressed	D48
s00188g11951	1,079	0,021	pleckstrin homology (PH) domain-containing protein	pleckstrin homology (PH) domain-containing protein [Arabidopsis thaliana] gi|332658453|gb|AEE83853.1|	Over Expressed	D48
s00129g09593	1,078	0,000	micronuclear linker histone polyprotein-like	PREDICTED: micronuclear linker histone polyprotein-like [Malus domestica]	Over Expressed	D48
s00062g06064	1,078	0,002	thiol protease aleurain-like	Thiol protease aleurain-like [Glycine soja]	Over Expressed	D48
s00034g03980	1,075	0,013	metal transporter Nramp6	Metal transporter Nramp6 [Glycine soja]	Over Expressed	D48
s00698g22740	1,070	0,008	zinc transporter 11	Zinc transporter 11 [Morus notabilis] gi|587874120|gb|EXB63273.1| Zinc transporter 11 [Morus notabilis]	Over Expressed	D48
s01189g27462	1,069	0,001	catalytic	catalytic, putative [Ricinus communis] gi|223536879|gb|EEF38517.1|	Over Expressed	D48
s00519g20026	1,067	0,002	multidrug resistance protein ABC transporter family	Multidrug resistance protein ABC transporter family [Theobroma cacao]	Over Expressed	D48
s00567g20824	1,067	0,001	TIM-barrel signal transduction protein isoform 2	TIM-barrel signal transduction protein isoform 2 [Theobroma cacao]	Over Expressed	D48
s01497g29029	1,066	0,000	transmembrane amino acid transporter family protein isoform 1	Transmembrane amino acid transporter family protein isoform 1 [Theobroma cacao] gi|508786327|gb|EOY33583.1|	Over Expressed	D48
s10596g33210	1,065	0,016	-	Transcription factor TGA7 [Morus notabilis]	Over Expressed	D48
s00010g01566	1,064	0,005	adiponectin receptor protein 2	Adiponectin receptor protein 2 [Morus notabilis]	Over Expressed	D48
s00291g15011	1,063	0,008	homeodomain-like transcriptional regulator isoform 1	Homeodomain-like transcriptional regulator isoform 1 [Theobroma cacao] gi|508701748|gb|EOX93644.1|	Over Expressed	D48
s00926g25360	1,061	0,044	homogentisate phytyltransferase 1	Homogentisate phytyltransferase 1 [Morus notabilis]	Over Expressed	D48
s00104g08410	1,060	0,016	pentatricopeptide repeat superfamily protein	Pentatricopeptide repeat superfamily protein, putative [Theobroma cacao] gi|508725197|gb|EOY17094.1|	Over Expressed	D48
s00103g08368	1,060	0,000	isocitrate dehydrogenase [NADP]	Isocitrate dehydrogenase [NADP] [Morus notabilis]	Over Expressed	D48
s00350g16501	1,058	0,000	xanthoxin dehydrogenase	Xanthoxin dehydrogenase [Morus notabilis]	Over Expressed	D48
s00868g24713	1,057	0,011	methyltransferase	Putative methyltransferase [Morus notabilis]	Over Expressed	D48
s00113g08873	1,055	0,007	-	gag-pol polyprotein [Populus deltoides]	Over Expressed	D48

s00779g23753	1,050	0,013	cytochrome P450 98A2	Cytochrome P450 98A2 [Morus notabilis] gi|587944214|gb|EXC30696.1|	Over Expressed	D48
s00008g01357	1,049	0,013	TSL-kinase interacting protein 1 isoform X2	PREDICTED: TSL-kinase interacting protein 1 isoform X2 [Vitis vinifera]	Over Expressed	D48
s00029g03549	1,049	0,015	triacylglycerol lipase	triacylglycerol lipase, putative [Ricinus communis]	Over Expressed	D48
s00293g15063	1,046	0,001	uncharacterized protein isoform 1	### Not characterized ###:Uncharacterized protein isoform 1 [Theobroma cacao]	Over Expressed	D48
s00028g03465	1,043	0,015	-	PREDICTED: protein ENHANCED DISEASE RESISTANCE 2-like [Prunus mume]	Over Expressed	D48
s00208g12637	1,042	0,046	-	***** No hits found ******	Over Expressed	D48
s00688g22598	1,035	0,009	beige-related and WD-40 repeat-containing protein isoform 1	Beige-related and WD-40 repeat-containing protein isoform 1 [Theobroma cacao] gi|508704266|gb|EOX96162.1|	Over Expressed	D48
s00151g10584	1,035	0,001	D-2-hydroxyglutarate dehydrogenase, mitochondrial	PREDICTED: D-2-hydroxyglutarate dehydrogenase, mitochondrial [Fragaria vesca subsp. vesca]	Over Expressed	D48
s00376g17137	1,034	0,002	plastid-lipid-associated protein 14, chloroplastic	PREDICTED: probable plastid-lipid-associated protein 14, chloroplastic [Jatropha curcas]	Over Expressed	D48
s01143g27135	1,032	0,011	CTL-like protein DDB_G0274487	PREDICTED: CTL-like protein DDB_G0274487 [Populus euphratica]	Over Expressed	D48
s00231g13352	1,031	0,002	similar to L484_010894	### Not characterized ###:hypothetical protein L484_010894 [Morus notabilis]	Over Expressed	D48
s00152g10627	1,031	0,031	1-acyl-sn-glycerol-3-phosphate acyltransferase 2	1-acyl-sn-glycerol-3-phosphate acyltransferase 2 [Morus notabilis]	Over Expressed	D48
s00080g07221	1,031	0,000	E3 ubiquitin-protein ligase ARI4	Putative E3 ubiquitin-protein ligase ARI4 [Aegilops tauschii]	Over Expressed	D48
s00377g17171	1,031	0,043	callose synthase 7	PREDICTED: callose synthase 7 [Prunus mume]	Over Expressed	D48
s00003g00532	1,028	0,012	ABC transporter F family member 1	ABC transporter F family member 1 [Morus notabilis]	Over Expressed	D48
s00982g25866	1,025	0,000	B3 domain-containing protein	B3 domain-containing protein [Morus notabilis]	Over Expressed	D48
s01345g28331	1,019	0,013	TMV resistance protein N	TMV resistance protein N [Morus notabilis]	Over Expressed	D48
s00607g21473	1,018	0,035	pheophorbide aoxygenase	Pheophorbide aoxygenase [Morus notabilis]	Over Expressed	D48
s01357g28395	1,016	0,026	TMV resistance protein N	TMV resistance protein N [Morus notabilis]	Over Expressed	D48
s00030g03616	1,004	0,030	similar to L484_019589	### Not characterized ###:hypothetical protein L484_019589 [Morus notabilis]	Over Expressed	D48

s00512g19903	1,003	0,000	endosomal targeting BRO1-like domain-containing protein isoform 2	Endosomal targeting BRO1-like domain-containing protein isoform 2 [Theobroma cacao] gi|508774320|gb|EOY21576.1|	Over Expressed	D48
s00014g02021	1,003	0,045	PRA1 family protein	PRA1 family protein [Theobroma cacao] gi|508779826|gb|EOY27082.1|	Over Expressed	D48
s00018g02498	1,002	0,006	-	PREDICTED: protein TRANSPARENT TESTA 12-like [Prunus mume]	Over Expressed	D48
s00254g14016	-6,824	0,043	F-box protein	F-box protein [Morus notabilis] gi|587871717|gb|EXB60973.1|	Under Expressed	D48
s00246g13769	-6,585	0,017	dCTP pyrophosphatase 1-like	PREDICTED: dCTP pyrophosphatase 1-like [Prunus mume]	Under Expressed	D48
s00470g19106	-6,342	0,003	-	PREDICTED: probable calcium-binding protein CML10 [Malus domestica]	Under Expressed	D48
s07340g32375	-6,158	0,022	-	***** No hits found ******	Under Expressed	D48
s00138g09991	-6,030	0,000	-	CDP-diacylglycerol--glycerol-3-phosphate 3-phosphatidyltransferase, putative, partial [Theobroma cacao]	Under Expressed	D48
s00004g00683	-6,019	0,009	galactose oxidase	galactose oxidase [Ziziphus jujuba]	Under Expressed	D48
s00469g19088	-5,169	0,001	-	***** No hits found ******	Under Expressed	D48
s00470g19104	-4,992	0,000	-	Calcium-binding EF-hand family protein, putative [Theobroma cacao]	Under Expressed	D48
s00004g00795	-4,986	0,025	dnaJ homolog subfamily C member 2	DnaJ homolog subfamily C member 2 [Morus notabilis]	Under Expressed	D48
s01043g26402	-4,583	0,000	-	### Not characterized ###:hypothetical protein L484_018186 [Morus notabilis]	Under Expressed	D48
s00075g06872	-4,569	0,021	-	***** No hits found ******	Under Expressed	D48
s00356g16647	-4,428	0,048	-	PREDICTED: G-type lectin S-receptor-like serine/threonine-protein kinase At1g34300 [Jatropha curcas]	Under Expressed	D48
s00470g19108	-4,368	0,000	-	Polcalcin Jun o 2 [Morus notabilis] gi|587932722|gb|EXC19749.1|	Under Expressed	D48
s00143g10196	-4,080	0,001	similar to L484_010790	### Not characterized ###:hypothetical protein L484_010790 [Morus notabilis]	Under Expressed	D48
s00375g17122	-3,912	0,000	similar to L484_010721	### Not characterized ###:hypothetical protein L484_010721 [Morus notabilis]	Under Expressed	D48
s00192g12067	-3,849	0,000	aspartic proteinase nepenthesin-1	Aspartic proteinase nepenthesin-1 [Morus notabilis]	Under Expressed	D48
s00300g15254	-3,725	0,016	xyloglucan endotransglucosylase/hydrolase protein 33	putative xyloglucan endotransglucosylase/hydrolase protein 33 [Morus notabilis] gi|587894084|gb|EXB82616.1|	Under Expressed	D48
s00470g19105	-3,551	0,000	-	PREDICTED: probable calcium-binding protein CML10 [Malus domestica]	Under Expressed	D48
s00723g23061	-3,506	0,002	-	Transcription factor WER [Morus notabilis]	Under Expressed	D48

s01278g27975	-3,428	0,000	two-component response regulator	Two-component response regulator [Morus notabilis]	Under Expressed	D48
s00469g19086	-3,341	0,007	-	***** No hits found ******	Under Expressed	D48
s00020g02730	-3,280	0,000	isoaspartyl peptidase/L-asparaginase 2	putative isoaspartyl peptidase/L-asparaginase 2 [Morus notabilis]	Under Expressed	D48
s00063g06158	-3,235	0,025	momilactone A synthase	Momilactone A synthase [Morus notabilis]	Under Expressed	D48
s00266g14365	-3,221	0,003	pectinesterase/pectinesterase inhibitor 6	putative pectinesterase/pectinesterase inhibitor 6 [Morus notabilis] gi|587902662|gb|EXB90901.1|	Under Expressed	D48
s00234g13451	-3,212	0,024	-	PREDICTED: transcription factor bHLH137-like [Prunus mume]	Under Expressed	D48
s06315g32096	-3,190	0,004	(RS)-norcoclaurine 6-O-methyltransferase	(RS)-norcoclaurine 6-O-methyltransferase [Morus notabilis]	Under Expressed	D48
s03907g31418	-3,121	0,014	-	***** No hits found ******	Under Expressed	D48
s00105g08469	-3,106	0,008	homogentisate phytyltransferase 2	Homogentisate phytyltransferase 2 [Morus notabilis]	Under Expressed	D48
s00055g05591	-3,103	0,001	ankyrin repeat-containing protein	Ankyrin repeat-containing protein [Morus notabilis]	Under Expressed	D48
s00458g18864	-2,994	0,007	-	(RS)-norcoclaurine 6-O-methyltransferase [Morus notabilis]	Under Expressed	D48
s00414g17945	-2,985	0,018	similar to L484_008667	### Not characterized ###:hypothetical protein L484_008667 [Morus notabilis]	Under Expressed	D48
s00289g14969	-2,972	0,010	feruloyl CoA ortho-hydroxylase 1-like	PREDICTED: feruloyl CoA ortho-hydroxylase 1-like [Prunus mume]	Under Expressed	D48
s00034g03992	-2,952	0,001	zinc finger protein 3	Zinc finger protein 3 [Morus notabilis] gi|587860504|gb|EXB50403.1|	Under Expressed	D48
s00232g13386	-2,942	0,042	-	### Not characterized ###:hypothetical protein L484_005247 [Morus notabilis]	Under Expressed	D48
s00263g14271	-2,936	0,034	-	***** No hits found ******	Under Expressed	D48
s00004g00779	-2,926	0,006	dnaJ homolog subfamily C member 1	DnaJ homolog subfamily C member 1 [Morus notabilis]	Under Expressed	D48
s00088g07626	-2,918	0,005	nitrate excretion transporter 6	putative nitrate excretion transporter 6 [Morus notabilis]	Under Expressed	D48
s04538g31615	-2,816	0,049	-	polygalacturonase inhibiting protein [Ulmus pumila]	Under Expressed	D48
s00009g01520	-2,638	0,002	subtilisin-like protease	Subtilisin-like protease [Morus notabilis]	Under Expressed	D48
s01007g26080	-2,591	0,036	-	PREDICTED: auxin-induced protein 15A-like [Malus domestica]	Under Expressed	D48
s00085g07457	-2,586	0,013	-	PREDICTED: CASP-like protein 4D1 [Fragaria vesca subsp. vesca]	Under Expressed	D48

s00111g08757	-2,581	0,000	aspartic proteinase nepenthesin-2	Aspartic proteinase nepenthesin-2 [Morus notabilis]	Under Expressed	D48
s00941g25505	-2,430	0,032	-	### Not characterized ###:hypothetical protein L484_020796 [Morus notabilis]	Under Expressed	D48
s00082g07325	-2,421	0,002	RING finger and CHY zinc finger domain-containing protein 1	RING fingerand CHY zinc finger domain-containing protein 1 [Morus notabilis] gi|587887097|gb|EXB75898.1|	Under Expressed	D48
s00040g04463	-2,409	0,002	-	GAST-like gene product family protein [Populus trichocarpa]	Under Expressed	D48
s00007g01254	-2,394	0,031	UDP-glycosyltransferase 91A1-like	PREDICTED: UDP-glycosyltransferase 91A1-like [Prunus mume]	Under Expressed	D48
s00122g09301	-2,334	0,004	-	***** No hits found ******	Under Expressed	D48
s00304g15359	-2,329	0,011	zinc transporter 1	Zinc transporter 1 [Morus notabilis] gi|587938034|gb|EXC24818.1|	Under Expressed	D48
s01452g28826	-2,322	0,032	-	PREDICTED: U-box domain-containing protein 35 [Jatropha curcas]	Under Expressed	D48
s00800g23992	-2,310	0,014	glutamine amidotransferase	Putative Glutamine amidotransferase [Morus notabilis]	Under Expressed	D48
s00003g00585	-2,224	0,000	lysine decarboxylase family protein, putative isoform 2	Lysine decarboxylase family protein, putative isoform 2 [Theobroma cacao] gi|508787142|gb|EOY34398.1|	Under Expressed	D48
s00066g06358	-2,194	0,001	glycine-rich protein 2b	Glycine-rich protein 2b [Morus notabilis]	Under Expressed	D48
s00235g13483	-2,152	0,041	centromere-associated protein E	Centromere-associated protein E [Morus notabilis]	Under Expressed	D48
s09224g32879	-2,139	0,012	lupeol synthase	Lupeol synthase [Morus notabilis] gi|587916353|gb|EXC04031.1|	Under Expressed	D48
s00047g05008	-2,135	0,005	glutaredoxin-C13	PREDICTED: glutaredoxin-C13 [Vitis vinifera]	Under Expressed	D48
s00312g15576	-2,129	0,046	WAT1-related protein At4g08300-like	PREDICTED: WAT1-related protein At4g08300-like [Prunus mume]	Under Expressed	D48
s00116g09014	-2,107	0,037	AP2/ERF and B3 domain-containing transcription factor RAV1	AP2/ERF and B3 domain-containing transcription factor RAV1 [Morus notabilis] gi|587864332|gb|EXB53997.1|	Under Expressed	D48
s25673g35834	-2,103	0,011	-	***** No hits found ******	Under Expressed	D48
s00006g01047	-2,100	0,000	phosphoenolpyruvate/phosphate translocator 2	Phosphoenolpyruvate/phosphate translocator 2 [Morus notabilis]	Under Expressed	D48
s00163g11038	-2,082	0,023	invertase inhibitor	Putative invertase inhibitor [Morus notabilis]	Under Expressed	D48
s00428g18250	-2,070	0,001	PAN domain-containing protein	PAN domain-containing protein [Morus notabilis]	Under Expressed	D48
s00172g11374	-2,028	0,013	40S ribosomal protein S17-4	40S ribosomal protein S17-4 [Morus notabilis]	Under Expressed	D48
s00625g21758	-2,017	0,044	myosin heavy chain, striated muscle	PREDICTED: myosin heavy chain, striated muscle [Prunus mume]	Under Expressed	D48

s00117g09060	-2,008	0,003	tryptophan aminotransferase-related protein 2	Tryptophan aminotransferase-related protein 2 [Morus notabilis]	Under Expressed	D48
s00309g15490	-1,992	0,023	-	CAMP-regulated phosphoprotein 19-related protein isoform 1 [Theobroma cacao] gi|590661689|ref|XP_007035743.1|	Under Expressed	D48
s00125g09454	-1,990	0,000	-	PREDICTED: protein ZINC INDUCED FACILITATOR 1-like isoform X2 [Populus euphratica]	Under Expressed	D48
s00048g05047	-1,981	0,008	CHASE domain containing histidine kinase protein isoform 1	CHASE domain containing histidine kinase protein isoform 1 [Theobroma cacao] gi|508709416|gb|EOY01313.1|	Under Expressed	D48
s25137g35769	-1,980	0,017	zinc finger protein 7	Zinc finger protein 7 [Morus notabilis] gi|587978388|gb|EXC63022.1|	Under Expressed	D48
s00024g03101	-1,973	0,021	homeobox-leucine zipper protein HAT3	Homeobox-leucine zipper protein HAT3 [Morus notabilis]	Under Expressed	D48
s00184g11780	-1,967	0,021	suppressorof disruption of TFIIS	PREDICTED: suppressorof disruption of TFIIS [Eucalyptus grandis]	Under Expressed	D48
s10083g33087	-1,899	0,019	-	Oxidative stress 3 [Theobroma cacao] gi|508783361|gb|EOY30617.1|	Under Expressed	D48
s00028g03486	-1,891	0,018	F-box only protein 13	F-box only protein 13 [Morus notabilis] gi|587886094|gb|EXB74928.1|	Under Expressed	D48
s00173g11386	-1,890	0,028	serine carboxypeptidase-like 35	Serine carboxypeptidase-like 35 [Theobroma cacao]	Under Expressed	D48
s00529g20205	-1,879	0,011	LRR receptor-like serine/threonine-protein kinase	putative LRR receptor-like serine/threonine-protein kinase [Morus notabilis] gi|587870238|gb|EXB59528.1|	Under Expressed	D48
s16786g34460	-1,876	0,006	isoprene synthase	isoprene synthase [Ficus septica]	Under Expressed	D48
s00621g21706	-1,875	0,035	thymidine kinase	Thymidine kinase [Morus notabilis] gi|587948661|gb|EXC34914.1|	Under Expressed	D48
s05800g31963	-1,875	0,000	subtilisin-like protease	Subtilisin-like protease [Morus notabilis]	Under Expressed	D48
s01621g29487	-1,867	0,015	-	PREDICTED: reticuline oxidase-like protein [Vigna radiata var. radiata]	Under Expressed	D48
s00107g08590	-1,862	0,033	-	***** No hits found ******	Under Expressed	D48
s00183g11778	-1,858	0,001	peptide/nitrate transporter	putative peptide/nitrate transporter [Morus notabilis]	Under Expressed	D48
s00072g06717	-1,847	0,039	two-component response regulator	Two-component response regulator [Morus notabilis]	Under Expressed	D48
s00279g14685	-1,836	0,013	similar to L484_010449	### Not characterized ###:hypothetical protein L484_010449 [Morus notabilis]	Under Expressed	D48
s00421g18096	-1,826	0,013	isoprene synthase	isoprene synthase [Ficus virgata]	Under Expressed	D48
s00246g13770	-1,815	0,041	polyubiquitin-like	PREDICTED: polyubiquitin-like [Prunus mume]	Under Expressed	D48
s00456g18833	-1,812	0,009	-	***** No hits found ******	Under Expressed	D48

s00910g25196	-1,810	0,018	transcription factor CPC	Transcription factor CPC [Morus notabilis]	Under Expressed	D48
s00045g04865	-1,805	0,013	cysteine/Histidine-rich C1 domain family protein	Cysteine/Histidine-rich C1 domain family protein [Theobroma cacao]	Under Expressed	D48
s01777g29948	-1,801	0,045	cytochrome P450 76C1	Cytochrome P450 76C1 [Morus notabilis] gi|587906958|gb|EXB94994.1|	Under Expressed	D48
s00109g08687	-1,794	0,000	F-box/kelch-repeat protein	F-box/kelch-repeat protein [Morus notabilis]	Under Expressed	D48
s02508g30923	-1,789	0,047	-	***** No hits found ******	Under Expressed	D48
s00212g12766	-1,788	0,000	transcription factor bHLH93-like	PREDICTED: transcription factor bHLH93-like [Malus domestica]	Under Expressed	D48
s00232g13384	-1,769	0,013	-	### Not characterized ###:hypothetical protein L484_005250 [Morus notabilis]	Under Expressed	D48
s00279g14695	-1,750	0,000	-	### Not characterized ###:hypothetical protein L484_015346 [Morus notabilis]	Under Expressed	D48
s00983g25877	-1,731	0,046	calcium-binding protein CML25	putative calcium-binding protein CML25 [Morus notabilis]	Under Expressed	D48
s00220g13014	-1,715	0,037	pectinesterase/pectinesterase inhibitor 35	putative pectinesterase/pectinesterase inhibitor 35 [Morus notabilis] gi|587909537|gb|EXB97446.1|	Under Expressed	D48
s00012g01860	-1,711	0,031	-	transmembrane protein, putative [Medicago truncatula]	Under Expressed	D48
s00390g17446	-1,701	0,000	similar to L484_021072	### Not characterized ###:hypothetical protein L484_021072 [Morus notabilis]	Under Expressed	D48
s00150g10530	-1,696	0,021	similar to L484_013410	### Not characterized ###:hypothetical protein L484_013410 [Morus notabilis]	Under Expressed	D48
s01389g28557	-1,696	0,007	flowering locus T-like protein	flowering locus T-like protein [Ficus carica]	Under Expressed	D48
s00080g07187	-1,694	0,000	anthocyanidin 3-O-glucosyltransferase 5-like	PREDICTED: anthocyanidin 3-O-glucosyltransferase 5-like [Fragaria vesca subsp. vesca]	Under Expressed	D48
s00033g03925	-1,693	0,005	palmitoyl-monogalactosyldiacylglycerol delta-7 desaturase	Palmitoyl-monogalactosyldiacylglycerol delta-7 desaturase [Morus notabilis] gi|587878284|gb|EXB67291.1|	Under Expressed	D48
s00270g14464	-1,687	0,004	F-box/kelch-repeat protein	F-box/kelch-repeat protein [Morus notabilis]	Under Expressed	D48
s00147g10400	-1,670	0,046	-	PREDICTED: protein BRANCHLESS TRICHOME [Jatropha curcas]	Under Expressed	D48
s00038g04336	-1,665	0,004	alpha-L-fucosidase 1	Alpha-L-fucosidase 1 [Morus notabilis] gi|587933925|gb|EXC20875.1|	Under Expressed	D48
s00056g05649	-1,661	0,000	T-complex protein 1 subunit beta	T-complex protein 1 subunit beta [Morus notabilis]	Under Expressed	D48
s00087g07597	-1,657	0,004	-	***** No hits found ******	Under Expressed	D48
s01144g27147	-1,648	0,044	-	***** No hits found ******	Under Expressed	D48
s00014g02106	-1,627	0,029	auxin-binding protein ABP19a	Auxin-binding protein ABP19a [Morus notabilis]	Under Expressed	D48

s00537g20336	-1,623	0,000	cyclic nucleotide-gated ionchannel 14	putative cyclic nucleotide-gated ionchannel 14 [Morus notabilis]	Under Expressed	D48
s00113g08855	-1,623	0,020	high mobility group B protein 9	High mobility group B protein 9 [Morus notabilis]	Under Expressed	D48
s00186g11907	-1,613	0,004	mitochondrial chaperone bcs1	putative mitochondrial chaperone bcs1 [Morus notabilis]	Under Expressed	D48
s00137g09958	-1,608	0,000	cytochrome P450 90A1	PREDICTED: cytochrome P450 90A1 [Prunus mume]	Under Expressed	D48
s02447g30886	-1,604	0,001	lupeol synthase	Lupeol synthase [Morus notabilis] gi|587852429|gb|EXB42556.1|	Under Expressed	D48
s00070g06634	-1,604	0,015	fatty acid desaturase 4, chloroplastic	PREDICTED: fatty acid desaturase 4, chloroplastic [Fragaria vesca subsp. vesca]	Under Expressed	D48
s00093g07928	-1,597	0,004	-	***** No hits found ******	Under Expressed	D48
s00833g24350	-1,595	0,016	nuc-1 negative regulatory protein preg	Nuc-1 negative regulatory protein preg, putative [Ricinus communis]	Under Expressed	D48
s00313g15588	-1,588	0,000	peptide transporter PTR3-A-like	PREDICTED: peptide transporter PTR3-A-like [Citrus sinensis]	Under Expressed	D48
s02038g30445	-1,587	0,013	-	isoprene synthase [Ficus septica]	Under Expressed	D48
s00603g21413	-1,585	0,000	-	PREDICTED: protein trichome birefringence-like 19 [Pyrus x bretschneideri] gi|694332764|ref|XP_009357004.1|	Under Expressed	D48
s00001g00130	-1,575	0,004	12-oxophytodienoate reductase 11	Putative 12-oxophytodienoate reductase 11 [Morus notabilis]	Under Expressed	D48
s00536g20333	-1,566	0,006	tRNA(adenine(34)) deaminase, chloroplastic	PREDICTED: tRNA(adenine(34)) deaminase, chloroplastic [Fragaria vesca subsp. vesca]	Under Expressed	D48
s00378g17180	-1,565	0,024	LIM and calponin domains-containing protein 1	LIM andcalponin domains-containing protein 1, putative [Theobroma cacao] gi|508720366|gb|EOY12263.1|	Under Expressed	D48
s00098g08156	-1,560	0,000	RING-H2 finger protein ATL1	RING-H2 finger protein ATL1 [Morus notabilis]	Under Expressed	D48
s01056g26517	-1,550	0,036	transcription factor bHLH63	PREDICTED: transcription factor bHLH63 [Prunus mume]	Under Expressed	D48
s26784g35980	-1,549	0,012	-	***** No hits found ******	Under Expressed	D48
s00183g11777	-1,533	0,009	peptide/nitrate transporter	putative peptide/nitrate transporter [Morus notabilis]	Under Expressed	D48
s00020g02719	-1,526	0,028	receptor protein kinase ZmPK1	Putative receptor protein kinase ZmPK1 [Morus notabilis]	Under Expressed	D48
s00079g07172	-1,524	0,007	-	PREDICTED: transcription factor IBH1-like [Prunus mume]	Under Expressed	D48
s00483g19354	-1,521	0,049	-	***** No hits found ******	Under Expressed	D48
s00002g00439	-1,504	0,000	sulfate transporter 3.1	Sulfate transporter 3.1 [Morus notabilis]	Under Expressed	D48
s00102g08339	-1,503	0,007	GDSL esterase/lipase 1	GDSL esterase/lipase 1 [Morus notabilis]	Under Expressed	D48
s00084g07446	-1,491	0,031	cysteine-rich repeat secretory protein 60	PREDICTED: cysteine-rich repeat secretory protein 60 [Populus euphratica]	Under Expressed	D48

s05042g31759	-1,491	0,000	dehydration-responsive element-binding protein 2A	Dehydration-responsive element-binding protein 2A [Morus notabilis]	Under Expressed	D48
s00471g19131	-1,477	0,007	-	heat shock 22 kDa protein, mitochondrial [Glycine max]	Under Expressed	D48
s00053g05460	-1,476	0,004	-	### Not characterized ###:hypothetical protein CICLE_v10012886mg [Citrus clementina]	Under Expressed	D48
s01396g28586	-1,476	0,002	-	### Not characterized ###:hypothetical protein L484_014646 [Morus notabilis]	Under Expressed	D48
s00610g21549	-1,473	0,013	-	Ubiquitin carboxyl-terminal hydrolase 8 [Morus notabilis]	Under Expressed	D48
s00088g07643	-1,472	0,027	pseudo response regulator, putative isoform 4	Pseudo response regulator, putative isoform 4 [Theobroma cacao]	Under Expressed	D48
s00237g13541	-1,456	0,000	granule-bound starch synthase 1, chloroplastic/amyloplastic-like	PREDICTED: granule-bound starch synthase 1, chloroplastic/amyloplastic-like [Pyrus x bretschneideri]	Under Expressed	D48
s00539g20370	-1,438	0,043	octicosapeptide/Phox/Bem1p family protein	Octicosapeptide/Phox/Bem1p family protein [Theobroma cacao]	Under Expressed	D48
s14207g33986	-1,430	0,015	transcription factor UNE10	Transcription factor UNE10 [Morus notabilis]	Under Expressed	D48
s00760g23512	-1,426	0,026	-	PREDICTED: protein LURP-one-related 5-like [Gossypium raimondii]	Under Expressed	D48
s01981g30359	-1,423	0,030	isoprene synthase	isoprene synthase [Ficus septica]	Under Expressed	D48
s00522g20093	-1,420	0,006	U-box domain-containing protein 28	U-box domain-containing protein 28 [Morus notabilis]	Under Expressed	D48
s00332g16075	-1,417	0,000	centrosomal proteinof 135 kDa	PREDICTED: centrosomal proteinof 135 kDa [Malus domestica]	Under Expressed	D48
s00082g07313	-1,412	0,030	ACT domain-containing protein ACR10-like	PREDICTED: ACT domain-containing protein ACR10-like [Glycine max]	Under Expressed	D48
s00361g16776	-1,405	0,004	zinc finger protein CONSTANS-LIKE 11	Putative zinc finger protein CONSTANS-LIKE 11 [Morus notabilis]	Under Expressed	D48
s00109g08670	-1,397	0,005	F-box protein	F-box protein [Morus notabilis] gi|587949568|gb|EXC35687.1| F-box protein [Morus notabilis]	Under Expressed	D48
s00052g05359	-1,397	0,020	phytochrome kinase substrate-related family protein	phytochrome kinase substrate-related family protein [Populus trichocarpa] gi|550322233|gb|ERP52266.1| phytochrome kinase substrate-related family protein [Populus trichocarpa]	Under Expressed	D48
s03945g31435	-1,396	0,027	-	Phosphoethanolamine N-methyltransferase 1 [Morus notabilis]	Under Expressed	D48

s00114g08945	-1,390	0,031	-	F-box [Gossypium arboreum]	Under Expressed	D48
s00162g11005	-1,389	0,001	glycosyltransferase family protein 64 protein C5	PREDICTED: glycosyltransferase family protein 64 protein C5 [Fragaria vesca subsp. vesca]	Under Expressed	D48
s00723g23060	-1,376	0,003	carbonic anhydrase 2-like isoform X1	PREDICTED: carbonic anhydrase 2-like isoform X1 [Populus euphratica] gi|743930576|ref|XP_011009543.1|	Under Expressed	D48
s00172g11375	-1,376	0,004	N-acetyltransferase p20	putative N-acetyltransferase p20 [Morus notabilis]	Under Expressed	D48
s00123g09320	-1,375	0,040	strigolactone esterase DAD2	PREDICTED: probable strigolactone esterase DAD2 [Vitis vinifera]	Under Expressed	D48
s00024g03146	-1,370	0,004	transcription factor UNE10	Transcription factor UNE10 [Morus notabilis]	Under Expressed	D48
s00100g08259	-1,369	0,001	GRAS family transcription factor family protein	GRAS family transcription factor family protein [Theobroma cacao]	Under Expressed	D48
s00864g24681	-1,365	0,010	-	PREDICTED: protein trichome birefringence-like 2 [Malus domestica]	Under Expressed	D48
s25646g35832	-1,364	0,021	-	***** No hits found ******	Under Expressed	D48
s00274g14545	-1,348	0,006	type I inositol-1,4,5-trisphosphate 5-phosphatase 2	Type I inositol-1,4,5-trisphosphate 5-phosphatase 2 [Morus notabilis] gi|587911951|gb|EXB99791.1|	Under Expressed	D48
s00812g24128	-1,347	0,001	-	PREDICTED: protein MIZU-KUSSEI 1 [Malus domestica]	Under Expressed	D48
s13313g33805	-1,345	0,027	CASP-like protein	CASP-like protein [Glycine soja]	Under Expressed	D48
s00013g01905	-1,341	0,001	baculoviral IAP repeat-containing protein 7-A	Baculoviral IAP repeat-containing protein 7-A [Morus notabilis]	Under Expressed	D48
s00166g11141	-1,339	0,015	similar to L484_008807	### Not characterized ###:hypothetical protein L484_008807 [Morus notabilis]	Under Expressed	D48
s00624g21741	-1,336	0,043	-	***** No hits found ******	Under Expressed	D48
s17582g34603	-1,330	0,000	inactive leucine-rich repeat receptor-like protein kinase	putative inactive leucine-rich repeat receptor-like protein kinase [Morus notabilis] gi|587916457|gb|EXC04120.1|	Under Expressed	D48
s01396g28585	-1,325	0,004	ribulose bisphosphate carboxylase small chain, chloroplastic	PREDICTED: ribulose bisphosphate carboxylase small chain, chloroplastic [Cucumis melo]	Under Expressed	D48
s00042g04623	-1,322	0,000	DNA double-strand break repair rad50 ATPase	DNA double-strand break repair rad50 ATPase [Theobroma cacao]	Under Expressed	D48
s00030g03707	-1,317	0,043	disease resistance protein RPP8	Disease resistance protein RPP8 [Morus notabilis]	Under Expressed	D48
s00018g02489	-1,314	0,034	transporter arsB	Putative transporter arsB [Morus notabilis]	Under Expressed	D48
s00002g00301	-1,312	0,000	similar to L484_009806	### Not characterized ###:hypothetical protein L484_009806 [Morus notabilis]	Under Expressed	D48
s00102g08338	-1,311	0,013	GDSL esterase/lipase 1	GDSL esterase/lipase 1 [Morus notabilis]	Under Expressed	D48
s12976g33734	-1,309	0,001	-	***** No hits found ******	Under Expressed	D48

s00015g02168	-1,306	0,026	ankyrin repeatand KH domain-containing protein 1	Ankyrin repeatand KH domain-containing protein 1 [Theobroma cacao]	Under Expressed	D48
s00278g14658	-1,301	0,003	-	### Not characterized ###:PREDICTED: uncharacterized protein LOC104234424 [Nicotiana sylvestris]	Under Expressed	D48
s00640g21976	-1,300	0,026	plasmamembrane intrinsic protein 1	plasmamembrane intrinsic protein 1;3 [Salix purpurea]	Under Expressed	D48
s13069g33751	-1,299	0,000	-	PREDICTED: probable LRR receptor-like serine/threonine-protein kinase At1g53440 [Pyrus x bretschneideri]	Under Expressed	D48
s00256g14079	-1,287	0,000	similar to L484_026093	### Not characterized ###:hypothetical protein L484_026093 [Morus notabilis]	Under Expressed	D48
s00248g13821	-1,286	0,022	18.1 kDa class I heat shock protein-like	PREDICTED: 18.1 kDa class I heat shock protein-like [Eucalyptus grandis]	Under Expressed	D48
s00427g18234	-1,286	0,008	uncharacterized protein L484_005224	### Not characterized ###:Uncharacterized protein L484_005224 [Morus notabilis]	Under Expressed	D48
s00234g13444	-1,280	0,000	similar to L484_013153	### Not characterized ###:hypothetical protein L484_013153 [Morus notabilis]	Under Expressed	D48
s00366g16919	-1,276	0,000	RNA binding-like protein	RNA binding-like protein [Theobroma cacao]	Under Expressed	D48
s01953g30314	-1,272	0,043	photosystem I subunit O-like	PREDICTED: photosystem I subunit O-like [Nicotiana tomentosiformis]	Under Expressed	D48
s00052g05356	-1,271	0,000	transcription factor TCP14	PREDICTED: transcription factor TCP14 [Cucumis melo]	Under Expressed	D48
s10657g33222	-1,271	0,000	carbonic anhydrase	Carbonic anhydrase [Morus notabilis] gi|587894062|gb|EXB82594.1|	Under Expressed	D48
s00054g05478	-1,269	0,001	epoxide hydrolase 3	PREDICTED: epoxide hydrolase 3 [Jatropha curcas]	Under Expressed	D48
s00433g18352	-1,267	0,000	floral homeotic protein APETALA 2	Floral homeotic protein APETALA 2 [Morus notabilis]	Under Expressed	D48
s00299g15204	-1,265	0,002	-	Autophagy-related 18 [Gossypium arboreum]	Under Expressed	D48
s00093g07917	-1,263	0,001	ribulose bisphosphate carboxylase/oxygenase activase, chloroplastic isoform X2	PREDICTED: ribulose bisphosphate carboxylase/oxygenase activase, chloroplastic isoform X2 [Vitis vinifera]	Under Expressed	D48
s00020g02729	-1,262	0,004	-	### Not characterized ###:hypothetical protein L484_006786 [Morus notabilis]	Under Expressed	D48
s00099g08215	-1,262	0,026	E3 ubiquitin-protein ligase COP1	E3 ubiquitin-protein ligase COP1 [Morus notabilis]	Under Expressed	D48
s00606g21472	-1,260	0,000	LRR receptor-like serine/threonine-protein kinase At1g07650	PREDICTED: probable LRR receptor-like serine/threonine-protein kinase At1g07650 [Prunus mume]	Under Expressed	D48
s00583g21099	-1,258	0,028	transcription factor bHLH30-like	PREDICTED: transcription factor bHLH30-like [Pyrus x bretschneideri]	Under Expressed	D48

s01174g27361	-1,250	0,000	subtilisin-like protease	Subtilisin-like protease [Morus notabilis]	Under Expressed	D48
s10019g33074	-1,245	0,040	similar to L484_002090	### Not characterized ###:hypothetical protein L484_002090 [Morus notabilis]	Under Expressed	D48
s11216g33358	-1,242	0,041	-	### Not characterized ###:hypothetical protein POPTR_0008s07760g [Populus trichocarpa]	Under Expressed	D48
s00629g21816	-1,233	0,017	pathogenesis-related thaumatin superfamily protein	Pathogenesis-related thaumatin superfamily protein [Theobroma cacao] gi|508698940|gb|EOX90836.1|	Under Expressed	D48
s00777g23722	-1,228	0,042	-	***** No hits found ******	Under Expressed	D48
s00099g08183	-1,225	0,027	pectate lyase 5	PREDICTED: probable pectate lyase 5 [Prunus mume]	Under Expressed	D48
s00005g00847	-1,225	0,007	cysteine-rich repeat secretory protein 15-like	PREDICTED: cysteine-rich repeat secretory protein 15-like [Prunus mume] gi|645253502|ref|XP_008232604.1|	Under Expressed	D48
s00028g03474	-1,212	0,046	glutamine dumper 4	Glutamine dumper 4, putative [Theobroma cacao]	Under Expressed	D48
s00407g17817	-1,207	0,012	15.4 kDa class V heat shock protein	PREDICTED: 15.4 kDa class V heat shock protein [Vigna radiata var. radiata]	Under Expressed	D48
s00677g22466	-1,205	0,011	psbP-like protein 1	PsbP-like protein 1 [Morus notabilis] gi|587846706|gb|EXB37165.1|	Under Expressed	D48
s00066g06329	-1,200	0,047	calcium-binding protein CML29	putative calcium-binding protein CML29 [Morus notabilis]	Under Expressed	D48
s00124g09397	-1,190	0,005	auxin efflux carrier component 5	Putative auxin efflux carrier component 5 [Morus notabilis]	Under Expressed	D48
s05192g31802	-1,186	0,000	-	PREDICTED: light-inducible protein CPRF2 [Pyrus x bretschneideri]	Under Expressed	D48
s00125g09435	-1,182	0,007	psbS	PsbS [Pisum sativum]	Under Expressed	D48
s00421g18111	-1,180	0,048	-	### Not characterized ###:hypothetical protein L484_009816 [Morus notabilis]	Under Expressed	D48
s00066g06328	-1,177	0,031	nitrate reductase	nitrate reductase [Morus alba] gi|595583466|gb|AHM76764.1|	Under Expressed	D48
s00080g07204	-1,175	0,037	-	***** No hits found ******	Under Expressed	D48
s00603g21412	-1,172	0,001	-	PREDICTED: protein trichome birefringence-like 19 [Populus euphratica]	Under Expressed	D48
s00841g24452	-1,170	0,000	G-type lectin S-receptor-like serine/threonine-protein kinase	G-type lectin S-receptor-like serine/threonine-protein kinase [Morus notabilis] gi|587873316|gb|EXB62507.1|	Under Expressed	D48
s00310g15541	-1,170	0,049	bidirectional sugar transporter SWEET2a	Bidirectional sugar transporter SWEET2a [Morus notabilis]	Under Expressed	D48
s00040g04475	-1,169	0,043	chaperone protein dnaJ 11	Chaperone protein dnaJ 11 [Morus notabilis]	Under Expressed	D48

s00764g23570	-1,166	0,011	DUF630 family protein	DUF630 family protein [Medicago truncatula]	Under Expressed	D48
s00025g03187	-1,163	0,023	mitochondrial carnitine/acylcarnitine carrier-like protein	PREDICTED: mitochondrial carnitine/acylcarnitine carrier-like protein [Nelumbo nucifera]	Under Expressed	D48
s01310g28138	-1,162	0,037	rubredoxin family protein	Rubredoxin family protein [Theobroma cacao]	Under Expressed	D48
s00130g09635	-1,162	0,000	cyclopropane-fatty-acyl-phospholipid synthase isoform 1	Cyclopropane-fatty-acyl-phospholipid synthase isoform 1 [Theobroma cacao] gi|590659737|ref|XP_007035210.1|	Under Expressed	D48
s00144g10239	-1,159	0,000	UDP-glycosyltransferase 74F2	UDP-glycosyltransferase 74F2 [Morus notabilis]	Under Expressed	D48
s00382g17277	-1,147	0,007	sialidase	Sialidase [Gossypium arboreum]	Under Expressed	D48
s00036g04174	-1,146	0,000	similar to L484_003808	### Not characterized ###:hypothetical protein L484_003808 [Morus notabilis]	Under Expressed	D48
s01289g28022	-1,146	0,009	serine/threonine-protein kinase BRI1-like 2-like	PREDICTED: serine/threonine-protein kinase BRI1-like 2-like [Citrus sinensis]	Under Expressed	D48
s00031g03737	-1,140	0,030	receptor-like protein kinase At5g61350	PREDICTED: probable receptor-like protein kinase At5g61350 [Cucumis melo]	Under Expressed	D48
s00141g10109	-1,140	0,019	-	***** No hits found ******	Under Expressed	D48
s10313g33141	-1,135	0,048	-	***** No hits found ******	Under Expressed	D48
s13434g33823	-1,134	0,022	-	PREDICTED: probable LRR receptor-like serine/threonine-protein kinase At1g53440 isoform X1 [Malus domestica]	Under Expressed	D48
s00315g15658	-1,132	0,000	receptor-like protein kinase	putative receptor-like protein kinase [Morus notabilis]	Under Expressed	D48
s00344g16346	-1,127	0,004	similar to L484_028070	### Not characterized ###:hypothetical protein L484_028070 [Morus notabilis]	Under Expressed	D48
s00150g10523	-1,127	0,008	aquaporin PIP2-1	Aquaporin PIP2-1 [Morus notabilis] gi|587896628|gb|EXB85110.1|	Under Expressed	D48
s00767g23610	-1,121	0,020	similar to L484_003463	### Not characterized ###:hypothetical protein L484_003463 [Morus notabilis]	Under Expressed	D48
s00531g20237	-1,121	0,046	BON1-associated protein 1-like	PREDICTED: BON1-associated protein 1-like [Malus domestica]	Under Expressed	D48
s00317g15722	-1,119	0,000	-	PREDICTED: transcription activator GLK1 [Vitis vinifera]	Under Expressed	D48
s00080g07183	-1,116	0,000	J domain-containing protein requiredfor chloroplast accumulation response 1-like	PREDICTED: J domain-containing protein requiredfor chloroplast accumulation response 1-like [Pyrus x bretschneideri]	Under Expressed	D48
s00002g00378	-1,113	0,016	photosystem II reaction center W protein	Photosystem II reaction center W protein [Morus notabilis]	Under Expressed	D48

s00389g17416	-1,104	0,008	psbP-like protein 1	PsbP-like protein 1 [Morus notabilis] gi|587876233|gb|EXB65325.1|	Under Expressed	D48
s01159g27250	-1,103	0,017	similar to L484_009861	### Not characterized ###:hypothetical protein L484_009861 [Morus notabilis]	Under Expressed	D48
s00470g19121	-1,100	0,014	similar to L484_003373	### Not characterized ###:hypothetical protein L484_003373 [Morus notabilis]	Under Expressed	D48
s00014g02038	-1,098	0,001	-	PREDICTED: protein YLS9 [Malus domestica]	Under Expressed	D48
s00102g08318	-1,098	0,007	von Willebrand factor A domain-containing protein	von Willebrand factor A domain-containing protein [Morus notabilis]	Under Expressed	D48
s00365g16874	-1,096	0,000	cinnamoyl-CoA reductase 1-like	PREDICTED: cinnamoyl-CoA reductase 1-like [Vitis vinifera]	Under Expressed	D48
s00339g16236	-1,090	0,045	similar to L484_008662	### Not characterized ###:hypothetical protein L484_008662 [Morus notabilis]	Under Expressed	D48
s00359g16723	-1,088	0,047	-	PREDICTED: protein SSUH2 homolog [Pyrus x bretschneideri]	Under Expressed	D48
s01598g29417	-1,081	0,001	-	PREDICTED: protein MIZU-KUSSEI 1 [Vitis vinifera]	Under Expressed	D48
s00207g12600	-1,079	0,009	uncharacterized protein LOC103327288	### Not characterized ###:PREDICTED: uncharacterized protein LOC103327288 [Prunus mume]	Under Expressed	D48
s01047g26453	-1,076	0,000	OTU domain-containing protein DDB_G0284757-like	PREDICTED: OTU domain-containing protein DDB_G0284757-like [Pyrus x bretschneideri]	Under Expressed	D48
s01080g26691	-1,074	0,000	ADP,ATP carrier protein ER-ANT1	ADP,ATP carrier protein ER-ANT1 [Morus notabilis]	Under Expressed	D48
s00387g17385	-1,073	0,000	zinc finger CCCH domain-containing protein 18	Zinc finger CCCH domain-containing protein 18 [Morus notabilis]	Under Expressed	D48
s18592g34793	-1,067	0,033	-	***** No hits found ******	Under Expressed	D48
s00017g02395	-1,065	0,008	PGR5-like protein 1B, chloroplastic	PREDICTED: PGR5-like protein 1B, chloroplastic [Fragaria vesca subsp. vesca]	Under Expressed	D48
s01603g29434	-1,064	0,044	GDP-mannose 3,5-epimerase 1	GDP-mannose 3,5-epimerase 1 [Morus notabilis]	Under Expressed	D48
s00318g15750	-1,061	0,039	ROP interactive partner 5 isoform 2	ROP interactive partner 5 isoform 2 [Theobroma cacao]	Under Expressed	D48
s00841g24450	-1,061	0,000	glyoxylate reductase/hydroxypyruvate reductase	Glyoxylate reductase/hydroxypyruvate reductase [Morus notabilis]	Under Expressed	D48

s00069g06517	-1,060	0,013	GATA transcription factor 22	Putative GATA transcription factor 22 [Morus notabilis]	Under Expressed	D48
s00649g22100	-1,057	0,007	ca2+-binding protein 1	Ca2+-binding protein 1 [Theobroma cacao]	Under Expressed	D48
s00851g24545	-1,054	0,001	receptor-like protein kinase THESEUS 1	Receptor-like protein kinase THESEUS 1 [Morus notabilis]	Under Expressed	D48
s00536g20334	-1,054	0,002	-	### Not characterized ###:PREDICTED: uncharacterized protein LOC104613531 [Nelumbo nucifera]	Under Expressed	D48
s00247g13785	-1,051	0,004	nudix hydrolase 1	Nudix hydrolase 1 [Morus notabilis] gi|587876479|gb|EXB65566.1|	Under Expressed	D48
s00351g16519	-1,051	0,032	stress enhanced protein 1	Stress enhanced protein 1, putative [Theobroma cacao]	Under Expressed	D48
s00014g02046	-1,048	0,004	hydrolase C777.06c-like isoform X1	PREDICTED: putative hydrolase C777.06c-like isoform X1 [Citrus sinensis]	Under Expressed	D48
s00767g23609	-1,048	0,011	-	Protein ApaG [Morus notabilis] gi|587838935|gb|EXB29618.1|	Under Expressed	D48
s00055g05571	-1,045	0,002	peptide transporter	putative peptide transporter [Morus notabilis]	Under Expressed	D48
s00429g18276	-1,041	0,000	glutamate receptor 3.2	Glutamate receptor 3.2 [Morus notabilis]	Under Expressed	D48
s00311g15565	-1,038	0,017	FK506-binding protein 4-like isoform X1	PREDICTED: FK506-binding protein 4-like isoform X1 [Brassica napus]	Under Expressed	D48
s00031g03721	-1,038	0,011	pentatricopeptide repeat-containing protein At1g62720-like	PREDICTED: pentatricopeptide repeat-containing protein At1g62720-like [Pyrus x bretschneideri]	Under Expressed	D48
s00213g12797	-1,037	0,035	peptidyl-prolyl cis-trans isomerase FKBP18	Peptidyl-prolyl cis-trans isomerase FKBP18 [Morus notabilis]	Under Expressed	D48
s00507g19822	-1,037	0,015	peroxiredoxin Q	Peroxiredoxin Q [Morus notabilis] gi|587847877|gb|EXB38195.1|	Under Expressed	D48
s00186g11873	-1,033	0,022	-	PREDICTED: protein ABIL2 [Prunus mume]	Under Expressed	D48
s00001g00127	-1,031	0,011	12-oxophytodienoate reductase 11	Putative 12-oxophytodienoate reductase 11 [Morus notabilis]	Under Expressed	D48
s00479g19277	-1,028	0,010	purple acid phosphatase 3	Purple acid phosphatase 3 [Morus notabilis]	Under Expressed	D48
s00019g02609	-1,028	0,008	similar to L484_005726	### Not characterized ###:hypothetical protein L484_005726 [Morus notabilis]	Under Expressed	D48
s00069g06527	-1,027	0,011	light-inducible protein CPRF2	PREDICTED: light-inducible protein CPRF2 [Prunus mume]	Under Expressed	D48
s01096g26796	-1,026	0,001	BOI-related E3 ubiquitin-protein ligase 1	PREDICTED: BOI-related E3 ubiquitin-protein ligase 1 [Fragaria vesca subsp. vesca]	Under Expressed	D48

s00186g11875	-1,026	0,021	carboxylesterase np	carboxylesterase np, putative [Ricinus communis]	Under Expressed	D48
s00862g24655	-1,026	0,006	similar to L484_023026	### Not characterized ###:hypothetical protein L484_023026 [Morus notabilis]	Under Expressed	D48
s00221g13048	-1,023	0,003	similar to L484_021121	### Not characterized ###:hypothetical protein L484_021121 [Morus notabilis]	Under Expressed	D48
s00274g14555	-1,021	0,000	ribose-phosphate pyrophosphokinase 1	Ribose-phosphate pyrophosphokinase 1 [Morus notabilis]	Under Expressed	D48
s00972g25788	-1,019	0,011	-	Protein bicaudal C-1-B-like protein [Morus notabilis]	Under Expressed	D48
s00178g11563	-1,015	0,023	E3 ubiquitin-protein ligase mib1	E3 ubiquitin-protein ligase mib1 [Morus notabilis]	Under Expressed	D48
s00057g05753	-1,010	0,016	membrane-associated kinase regulator 1	PREDICTED: probable membrane-associated kinase regulator 1 [Sesamum indicum]	Under Expressed	D48
s00051g05278	-1,007	0,000	UDP-glycosyltransferase 71C3	UDP-glycosyltransferase 71C3 [Morus notabilis]	Under Expressed	D48
s00039g04383	-1,004	0,047	ribose-5-phosphate isomerase	putative ribose-5-phosphate isomerase [Morus notabilis]	Under Expressed	D48
s01358g28405	-1,004	0,004	similar to L484_021354	### Not characterized ###:hypothetical protein L484_021354 [Morus notabilis]	Under Expressed	D48
s00900g25093	-1,003	0,027	similar to L484_023812	### Not characterized ###:hypothetical protein L484_023812 [Morus notabilis]	Under Expressed	D48
s00234g13464	-1,001	0,015	UDP-glycosyltransferase 85A5-like isoform X1	PREDICTED: UDP-glycosyltransferase 85A5-like isoform X1 [Prunus mume]	Under Expressed	D48
s00346g16390	-1,000	0,000	NAC domain-containing protein 94	Putative NAC domain-containing protein 94 [Morus notabilis]	Under Expressed	D48

Table S3. qRT/PCR analysis. The ID code of genes analyzed by qRT-PCR (according to Mori et al., 2017), sequence of Forward (F) and Reverse (R) PCR primers, fold change expression values as ratio between salt treated and control plants and P value of the difference are reported. The three housekeeping reference genes are also reported.

ID: s01287g28013 (aldehyde dehydrogenase)			
F: **GCAGCAGGCATTGTGACAAA**	R: **GCTCGGATTGACCTTGAAACA**		
**Fold change reference**	**Fold change gene**	**P value Anova one-way**	
**1**	**3.415305463**		
**1**	**2.690303073**	**0.000881**	
**1**	**2.828602881**		
ID: s00040g04475 (Chaperone protein)			
F: **ATCCACGAAGCGTATTCCAC**	R: **ATCGGTTTCCCATGTTCTTG**		
**Fold change reference**	**Fold change gene**	**P value Anova one-way**	
**1**	**0.150455062**		
**1**	**0.037764794**	**0.000018**	
**1**	**0.032260695**		
ID: s000212g12766 (Transcription factor bHLH)			24 d
F: **CCAGCTCCTCTTCTTCCATAGACA**	R: **CCCCGACATTTCCCATCA**		
**Fold change reference**	**Fold change gene**	**P value Anova one-way**	
**1**	**0.05178047**		
**1**	**0.110895916**	**0.00001**	
**1**	**0.050968089**		
ID: s00679g22491 (Nuclear Transcription Factor Y)			
F: **ATACCACTCGCTCTTGGGTGAT**	R: **GGTGCTGCCCCGAGAAT**		
**Fold change reference**	**Fold change gene**	**P value Anova one-way**	
**1**	**3.719102942**		
**1**	**2.019555909**	**0.020774**	
**1**	**3.885867461**		
ID: s00139g10024 (Highly ABA induced PP2C)			
F: **GCGAAATGGTCAAAGCATCT**	R: **TGTCGTGTCCGTATTCCGTA**		48 d
**Fold change reference**	**Fold change gene**	**P value Anova one-way**	
**1**	**5.78557063**	**0.080443**	

**1**	**13.86213404**	
**1**	**4.218150485**	
ID: s00679g22491 (NuclearTranscriptionFactorY)		
F: **ATACCACTCGCTCTTGGGTGAT**	R: **GGTGCTGCCCCGAGAAT**	
**Fold change reference**	**Fold change gene**	**P value Anova one-way**
**1**	**2.604361916**	
**1**	**3.057568449**	**0.000246**
**1**	**2.655527536**	

ID: s00085g07448 (actin-encoding gene)		Ref.
F: **CAATCGGTGCTGAGCGTTT**	R: **TCCTATCAAGGATGGCTGGAA**	
ID: s00104g08427 (alpha-tubulin-encoding gene)		
F: **GATGTTCCGCCGTGTGAGT**	R: **CCAATGCAAGAAAGCCTTCCT**	
ID: LN999821 (18S rRNA)		
F: **GGCGGATGTTGCTTTTAGGA**	R: **TTCAGCCTTGCGACCATACTC**	

Table S4. Up (log FC > 1) and down-regulated (log FC < -1) genes between early (24 days) and late (48 days) saline stress. Log FC= $\log_2$ fold change.

ID	D24 LOG FC	D48 LOG FC	DESCRIPTION	REGULATION
S00694G22691	4,87	5,58	Predicted protein	Up
S00785G23824	3,08	3,51	Isoflavone 2'-hydroxylase	Up
S00018G02534	2,81	3,5	late blight resistance R1B-19	Up
S00003G00605	2,24	3,25	Predicted protein	Up
S00001G00053	1,96	2,53	mitochondrial protein AtMg00810-like	Up
S00786G23838	1,06	2,41	Subtilisin protease	Up
S00247G13804	2,21	2,05	E3 ubiquitin-protein RHA2A	Up
S00444G18584	1,21	1,99	Predicted protein	Up
S00331G16057	1,36	1,95	Serine/threonine kinase SAPK3, isoform 2	Up
S00261G14202	1,74	1,88	acyl-protein thioesterase 2	Up
S00017G02349	1,15	1,86	Tropinone reductase	Up
S00679G22491	2	1,83	Nuclear transcription factor Y subunit A	Up
S02020G30426	1,59	1,82	Ty1-copia retrotranspon	Up
S00067G06394	1,71	1,76	Serine/threonine kinase	Up
S00423G18151	1,36	1,73	Predicted protein	Up
S00038G04301	1,43	1,71	Leucine-rich repeat receptor protein kinase EXS	Up
S00319G15780	1,65	1,6	Alpha-1,4 glucan phosphorylase L isozyme	Up
S00041G04572	1,56	1,57	Mitochondrialacylcarnitinecarrier protein(CACL)	Up
S00057G05768	1,05	1,57	Predicted membrane protein	Up
S00114G08941	2,19	1,49	DNA ligase	Up
S00014G02018	1,48	1,46	ABC transporter C family member 8	Up
S00237G13551	1,08	1,4	Two-component response regulator	Up
S00147G10413	1,73	1,37	Granule-bound starch synthase 1	Up
S00100G08254	1,9	1,34	Phosphoglucomutase	Up
S00080G07240	6,92	1,28	Cytochrome P450	Up
S01090G26759	1,5	1,28	J domain-containing protein	Up

			chloroplast isoform X1	
S00014G02014	1,25	1,26	ABC transporter C family member 8	Up
S00001G00054	1,09	1,24	Predicted protein	Up
S00281G14755	1,7	1,24	Adagio protein 3	Up
S00033G03917	1,07	1,19	aspartate, glycine, lysine and serine-rich protein	Up
S00896G25044	1,46	1,19	Ribonuclease J	Up
S00051G05309	1,04	1,18	protein DA1-related 2	Up
S00293G15070	1,12	1,16	senescence-associated family protein	Up
S00153G10646	2,57	1,15	B3 domain-containing transcription factor VRN1	Up
S00704G22801	1,2	1,15	Gamma-irradiation andmitomycinc induced 1 isoform 1	Up
S00248G13812	1,19	1,12	Tubulin beta-1 chain	Up
S00003G00556	1,13	1,11	Putative arsenical pump-driving ATPase	Up
S00698G22740	1,18	1,07	Zinc transporter 11	Up
S10596G33210	1,37	1,06	Transcription factor TGA7	Up
S00470G19104	-5,15	-4,99	Calcium-binding EF-hand family protein	Down
S00458G18864	-4,33	-2,99	(RS)-norcoclaurine 6-O-methyltransferase	Down
S00004G00779	-4,27	-2,93	DnaJ homolog subfamily C	Down
S00192G12067	-4,12	-3,85	Aspartic proteinase nepenthesin	Down
S00040G04463	-4	-2,41	GAST-like gene	Down
S06315G32096	-3,68	-3,19	(RS)-norcoclaurine 6-O-methyltransferase	Down
S00116G09014	-3,55	-2,11	AP2/ERF and B3 domain-containing transcription factor RAV1	Down
S03907G31418	-3,55	-3,12	Predicted protein	Down
S00082G07325	-3,48	-2,42	RING finger and CHY zinc finger domain-containing protein	Down
S00040G04475	-3,27	-1,17	Chaperone protein dnaJ 11	Down
S00234G13451	-3,23	-3,21	transcription factor bHLH137	Down
S00232G13386	-3,14	-2,94	Predicted protein	Down

S00143G10196	-3,1	-4,08	Predicted protein	Down
S00212G12766	-3,07	-1,79	transcription factor bHLH93	Down
S00085G07457	-3,04	-2,59	CASP-like protein 4D1	Down
S00220G13014	-2,91	-1,71	pectinesterase	Down
S00640G21976	-2,76	-1,3	plasmamembraneintrinsicprotein 1	Down
S00470G19108	-2,61	-4,37	Polcalcin Jun o 2	Down
S00270G14464	-2,58	-1,69	F-box/kelch-repeat protein	Down
S00469G19086	-2,53	-3,34	Predicted protein	Down
S00800G23992	-2,44	-2,31	Glutamine amidotransferase	Down
S00483G19354	-2,42	-1,52	Predicted protein	Down
S00278G14658	-2,4	-1,3	Predicted protein	Down
S00088G07643	-1,98	-1,47	Pseudo response regulator, isoform 4	Down
S00539G20370	-1,98	-1,44	Octicosapeptide/Phox/Bem1p	Down
S02447G30886	-1,94	-1,6	Lupeol synthase	Down
S00309G15490	-1,93	-1,99	CAMP-regulated phosphoprotein 19, isoform 1	Down
S00232G13384	-1,89	-1,77	Predicted protein	Down
S00003G00585	-1,74	-2,22	Lysine decarboxylase family protein, isoform 2	Down
S00378G17180	-1,73	-1,56	LIM and calponin domains-containing protein	Down
S00361G16776	-1,68	-1,41	CONSTANS-LIKE 11 (zinc finger)	Down
S00098G08156	-1,52	-1,56	RING-H2 finger protein ATL1	Down
S00006G01047	-1,39	-2,1	Phosphoenolpyruvate/ phosphate translocator	Down
S00102G08339	-1,29	-1,5	GDSL esterase/lipase	Down
S00137G09958	-1,25	-1,61	cytochrome P450	Down
S00311G15565	-1,24	-1,04	FK506-binding protein 4 isoform X1	Down
S00162G11005	-1,22	-1,39	glycosyltransferase family protein	Down
S00537G20336	-1,22	-1,62	cyclic nucleotide-gated ionchannel	Down
S00427G18234	-1,21	-1,29	Predicted protein	Down
S01598G29417	-1,2	-1,08	protein MIZU-KUSSEI 1	Down
S01096G26796	-1,18	-1,03	BOI-related E3 ubiquitin-proteinligase	Down

S00812G24128	-1,16	-1,35	protein MIZU-KUSSEI 1	Down
S00764G23570	-1,12	-1,17	DUF630 family protein	Down
S00013G01905	-1,06	-1,34	IAP repeat-containing protein	Down
S00344G16346	-1,06	-1,13	Predicted protein	Down
S00274G14555	-1,02	-1,02	Ribose-phosphate pyrophosphokinase	Down

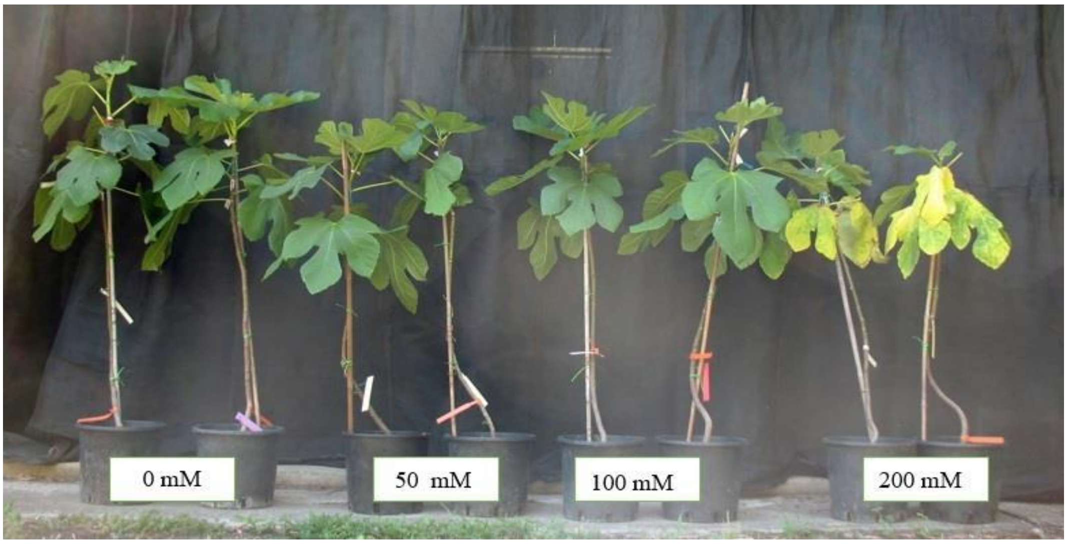

Fig. S1. *Ficus carica* (cv. Dottato) plants after 7 weeks of salinization at different NaCl concentrations. The transcriptome analysis was performed on 0 and 100 mM NaCl-treated plants only.

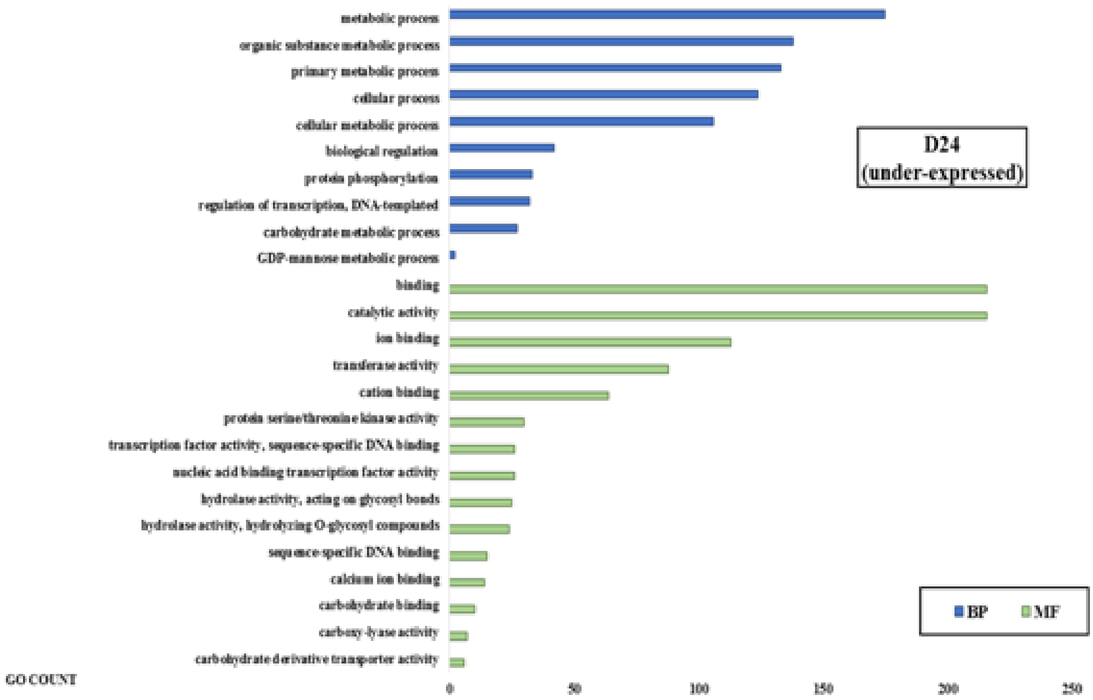

Fig. S2. Enriched GO terms in genes under-expressed in leaves of *F. carica* after salt treatment. BP = Biological processes; MF = Molecular function.

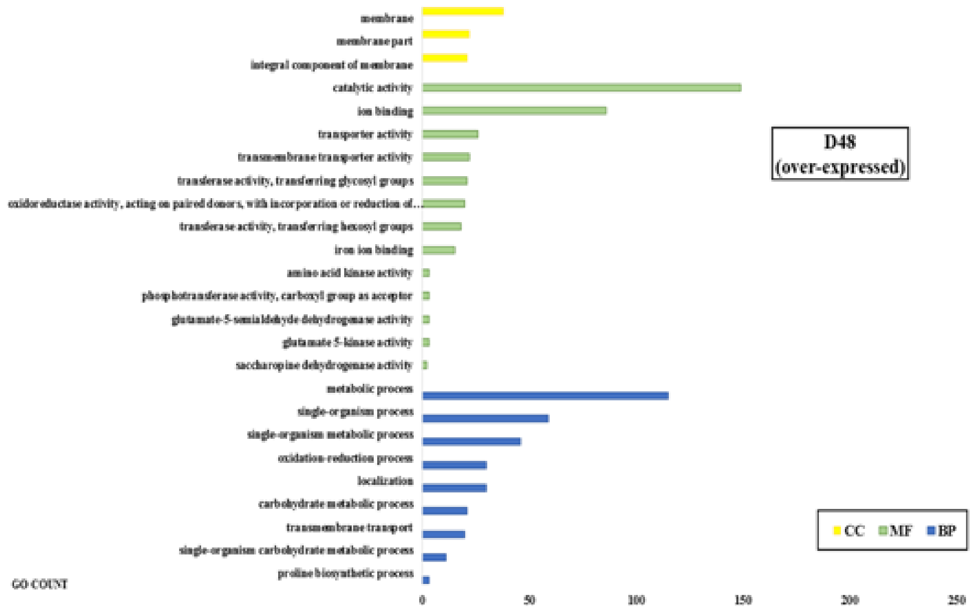

Fig. S3. Enriched GO terms in genes over-expressed in leaves of *F. carica* after salt treatment. CC = Cellular components; MF = Molecular function; BP = Biological process.

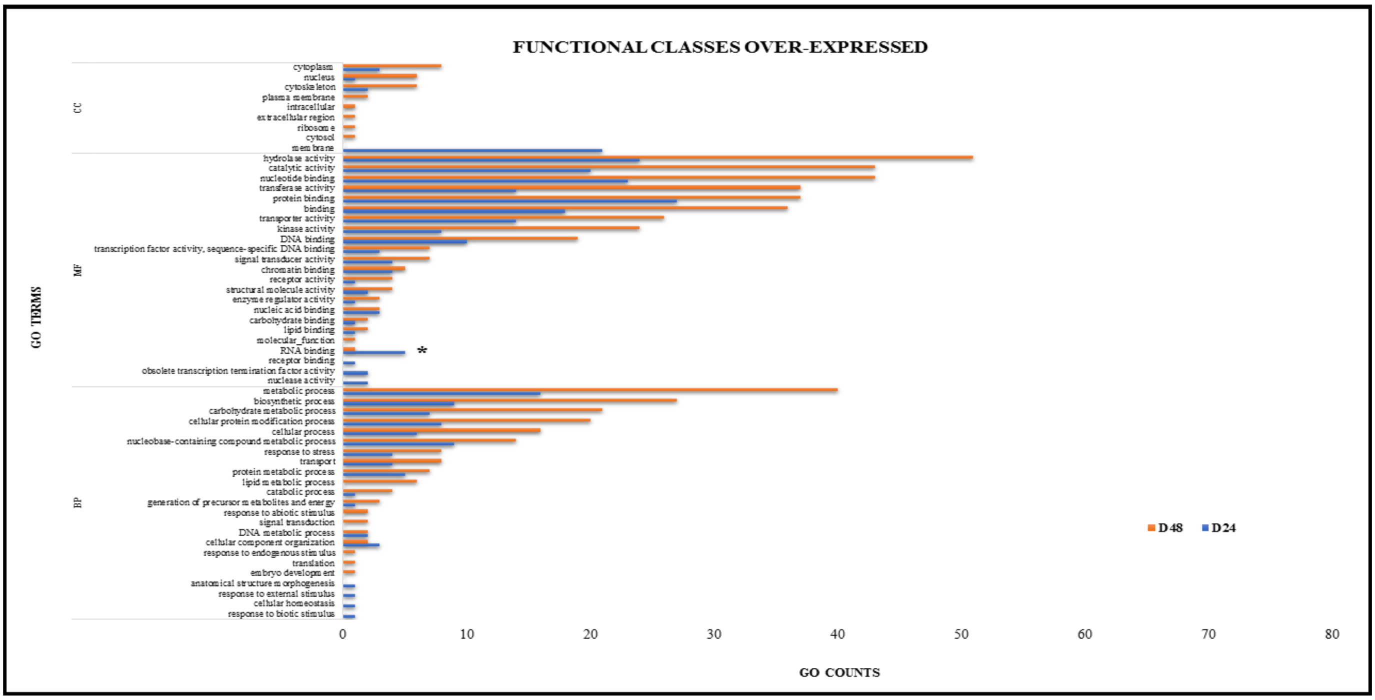

Fig. S4. GO terms of D24 and D48 over-expressed genes. Blue and orange bars show respectively distribution of GO-slim at D24 (blue) and D48 (red). BP = Biological processes; MF = Molecular functions; CC = Cellular components. Asterisks indicate that difference is significant at p<0.05.

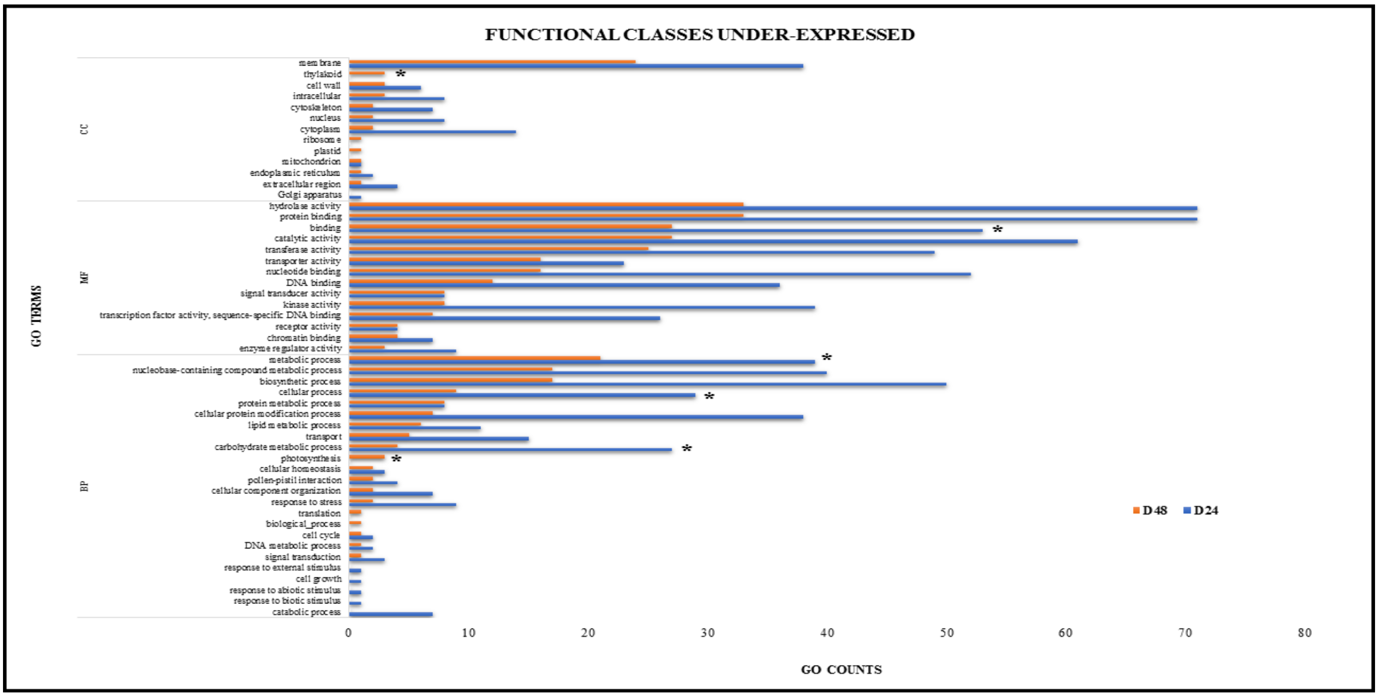

Fig. S5. GO terms of D24 and D48 under-expressed genes. Blue and orange bars show respectively distribution of GO-slim at D24 (blue) and D48 (red). BP = Biological processes; MF = Molecular functions; CC = Cellular components. Asterisks indicate that difference is significant at p<0.05

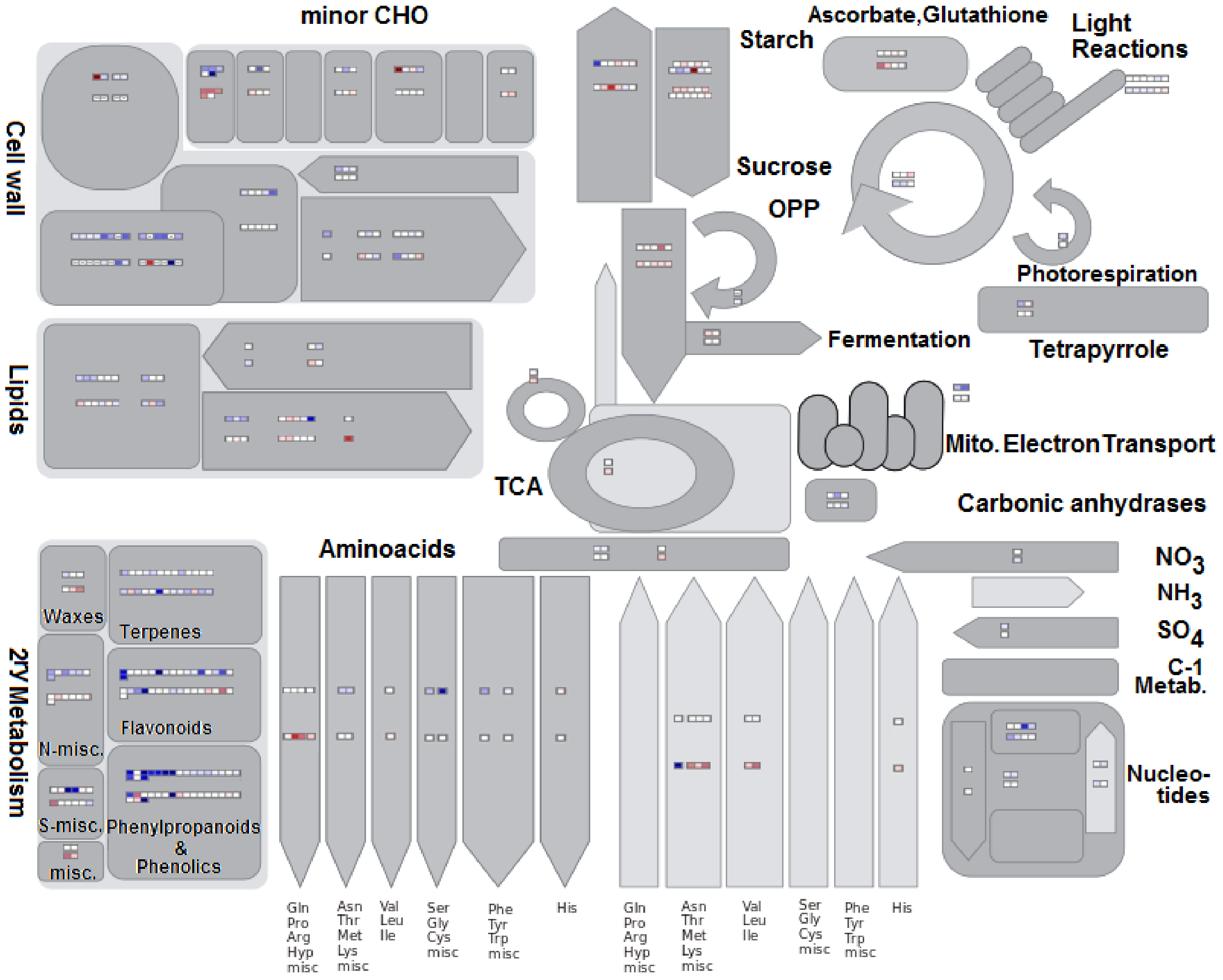

Fig. S6. MAPMAN sketch of metabolic pathways comparing up- and down-regulated genes (small squares) under salt treatments at D24 (above) and D48 (below). The differential expression scale ranges from dark blue (log FC < -4.5) to dark red (log FC > 4.5).
